# Recent Advances in Electrochemical Immunosensors

**DOI:** 10.3390/s17040794

**Published:** 2017-04-07

**Authors:** Benoît Piro, Steeve Reisberg

**Affiliations:** University Paris Diderot, Sorbonne Paris Cité, ITODYS, UMR 7086 CNRS, 15 rue J-A de Baïf, 75205 Paris CEDEX 13, France; steeve.reisberg@univ-paris-diderot.fr

**Keywords:** immunosensors, electrochemical sensors, nanoparticules, carbon nanotubes, graphene, enzyme, redox probe, antibodies, hapten, dendrimers, magnetic nanoparticles, ionic liquids, ELISA, sandwich-type immunosensors, competitive immunosensor

## Abstract

Immunosensors have experienced a very significant growth in recent years, driven by the need for fast, sensitive, portable and easy-to-use devices to detect biomarkers for clinical diagnosis or to monitor organic pollutants in natural or industrial environments. Advances in the field of signal amplification using enzymatic reactions, nanomaterials such as carbon nanotubes, graphene and graphene derivatives, metallic nanoparticles (gold, silver, various oxides or metal complexes), or magnetic beads show how it is possible to improve collection, binding or transduction performances and reach the requirements for realistic clinical diagnostic or environmental control. This review presents these most recent advances; it focuses first on classical electrode substrates, then moves to carbon-based nanostructured ones including carbon nanotubes, graphene and other carbon materials, metal or metal-oxide nanoparticles, magnetic nanoparticles, dendrimers and, to finish, explore the use of ionic liquids. Analytical performances are systematically covered and compared, depending on the detection principle, but also from a chronological perspective, from 2012 to 2016 and early 2017.

## 1. Introduction

The field of electrochemical immunosensors is very rich and dynamic. To give a general but concise overview of the current state-of-the-art, we have focused this review only on the major publications of the last five years (2012–2017), i.e., those which could justify of several tens of citations to date for the oldest (2012–2014), and at least some for the most recent ones.

Of course, several reviews are already available on this topic, but these do not cover the period covered by this review, or are less general. A relatively brief review published by Ricci et al. in 2012 [1], presented a guide to all researchers interested in entering the electrochemical immunosensor domain, by reviewing the literature over the 2008–2012 period and focusing particularly on practical aspects. Another review, published by Yang et al., also in 2012 [2], focused on new trends in signal amplification in enzyme-based immunosensors (combination of enzymatic reactions, multienzyme labels, use of magnetic beads...). The other reviews available since 2012 are more specialized, focusing on materials, transductions or applications. Concerning materials and transductions, Hasanzadeh et al. [3] dealt in 2013 with mesoporous silica materials for use in electrochemical immunosensing. Pei et al. [4] published also in 2013 a review dealing specially with sandwich-type immunosensors exploiting nanostructured materials. More recently, in 2016, Arduini et al. [5] reviewed more particularly screen-printed electrodes modified by nanomaterials such as carbon nanotubes, graphene, metallic nanoparticles (gold, silver and magnetic nanoparticles) coupled with enzymes or antibodies and showed how it could improve performances. They gave, as perspective, some recent examples of paper-based, wearable or smartphone-driven devices. Concerning reviews which focus on precise applications rather than on materials, Wan et al. [6] dealt in 2013 with generalities on point-of-care diagnostics for early detection of diseases. They also reviewed attempts to propose integrated systems, several being commercially available today. Bahadır et al. [7] did the same in 2015 for early clinical diagnostics of cancer and cardiac diseases. More focused on one given application, or specific for a class of biomarkers or pollutants, Chikkaveeraiah et al. [8] reviewed in 2012 the most recent advances at that time in electrochemical immunosensors for detection of cancer protein biomarkers, with strategies to increase densities of capture molecules and sensitivities. On their side, in 2013, Vidal et al. [9] reviewed electrochemical affinity biosensors for detection of mycotoxins in food. This review focused on affinity probes in general but antibodies, including recombinant antibodies, are addressed. Also in 2013, Diaconu et al. [10] reviewed electrochemical immunosensors for precise applications in breast and ovarian cancer. Finally, Campuzano et al. [11] reviewed very recently (2017) electrochemical bioaffinity sensors for salivary biomarkers.

This review presents the most recent advances in electrochemical immunodetection using enzymes and redox reactions for transduction and amplification, or enzyme-less strategies using innovative inorganic catalylists. We will focus first on classical electrode substrates, then move to carbon-based nanostructured ones including carbon nanotubes, graphene and other carbon materials. We will follow with the use of metal or metal-oxide nanoparticles, magnetic nanoparticles, dendrimers and, finally, we cite a few works using ionic liquids.

## 2. Glossary of Acronyms

Because a lot of different structures or molecules are cited here, sometimes repeatedly, we used most of the time acronyms instead of the full names; for the sake of clarity, a glossary of acronyms is provided below (Table 1).

## 3. Discussion

Works were sorted depending on whether they rely on enzymes for transduction (enzyme-based), or not (enzyme-less) and their analytical performances are given systematically. In each section or sub-section, articles are cited by type of detected target, then by chronological order from 2012 to 2017 to evidence progressive evolutions.

### 3.1. Conventionnal Electrode Substrates

Most of the works reviewed here report the use of nanomaterials; however, conventional electrodes and materials were also investigated during the considered period.

#### 3.1.1. Enzyme-Based Immunosensors

Enzymes are generally used not for detection itself but for transduction and amplification, taking profit of the enzyme turn-over (for one event of biorecognition, one antibody or antigen captured, several molecules of enzyme product are produced at the vicinity of the electrode surface and electrochemically detected). HRP, horse radish peroxidase, is probably the most used enzyme label in immunosensors because it is commercially available and catalyzes the oxidation of numerous chromogenic substrates using H_2_O_2_ as co-substrate. However, some other enzymes are also encountered such as alkaline phosphatase (AlkP) and glucose oxidase (GOx).

• Detection of antibodies

The term “immunosensor” could mean that the target could be either an antigen (Ag) or an antibody (Ab). Even if the most popular approach remains targeting Ag using antibodies, some works report Ab detection, as for example in the framework of serological diagnosis, i.e., the research and the determination of specific antibodies linked to a pathogenic infection.

**Figure 1:**
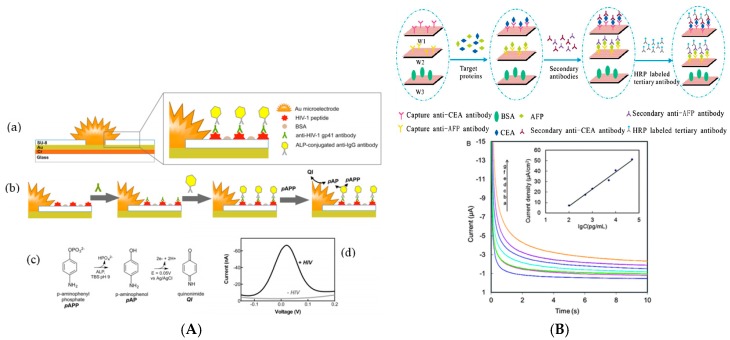
(**A**) (**a**) Au microelectrodes obtained by electroreduction of HAuCl_4_ in 5 μm apertures; (**b**) Immunorecognition. HIV-1 antigens are covalently immobilized on a SU-8 layer, then target antibodies are bound, followed by secondary AlkP-labelled anti-IgG binding. p-Aminophenyl phosphate (pAPP) is added as substrate; (**c**) Transduction: (**c**) AlkP converts pAPP to p-aminophenol which is electrooxidized at the electrode and produces a current proportional to the amount of target antibody bound to the sensor; (**d**) Resulting DPV. Reprinted with permission from [12]. Copyright 2013 American Chemical Society; (**B**) *Above*. Detection principle of the screen-printed bi-analyte array, including a BSA (reference) electrode. *Below*. Change in current for different concentrations of AFP, for blank (lowest current) to 50 ng mL^−1^ (highest current); calibration curve in inset. Adapted from [15] with permission from The Royal Society of Chemistry.

In 2013, Bhimji et al. [12] described an interesting route to detect human immunodeficiency virus (HIV) antibodies by immobilization of antigenic peptides derived from a complex transmembrane protein, HIV-1 gp41 or HIV-2 gp36, covalently attached to a SU-8 substrate (a negative epoxy photoresist) close to microelectrodes. The detection of HIV antibodies was achieved using an alkaline phosphatase (AlkP)-conjugated secondary antibody (Figure 1A). The linear detection range was reported between 1 ng mL^−1^ and 1 μg mL^−1^, with a limit of detection (LoD) of 1 ng mL^−1^ (6.7 pM). More recently in 2016, Montes et al. [13] used a composite graphite-epoxy substrate into which an HRP-labelled antibody was incorporated, for detection of IgG. Transduction was classical, with a competitive assay using H_2_O_2_ and hydroquinone (HQ) in solution. Amperometric measurements (reduction of benzoquinone BQ into HQ at the electrode) led to a high LoD of 1.4 μg mL^−1^ and a relatively reduced linear range up to 2.8 μg mL^−1^. There is no other recent cited work reporting enzyme-based immunosensor for detection of antibodies, the literature being now more focused on nanostructured electrodes rather than on classical substrates.

• Detection of antigens

Detection of antigens on such substrates is still popular. For example, in 2012, Ojeda et al. [14] described an electrochemical immunosensor for estradiol sensing, based on carbon screen-printed electrodes (SPE) sequentially modified with *p*-aminobenzoic acid, streptavidin and biotinylated anti-estradiol. Transduction was performed by applying a competitive immunoassay between peroxidase-labeled estradiol (HRP–estradiol) and estradiol for the binding sites of the immobilized antibodies. The reaction between estradiol and biotinylated anti-estradiol was amperometrically detected by addition of H_2_O_2_ in the presence of HQ. The linear range was between 1 and 250 pg mL^−1^ and the LoD was 0.77 pg mL^−1^. Also in 2012, Qi et al. [15] reported an array of carbon SPE for simultaneous detection of several tumor biomarkers such as carcinoembryonic antigen (CEA) and α-fetoprotein (AFP, a tumor markers used in the early diagnosis of cancer). Electrodes were modified by grafting *p*-phenylenediamine via the diazonium route, followed by crosslinking the primary capture antibody using a Schiff base reaction. Transduction was made by using a sandwich assay with HRP-labelled secondary antibodies. (Figure 1B). The detection range was from 0.10 to 50 ng mL^−1^ and the LoD of ca. 40 pg mL^−1^. 

**Figure 2:**
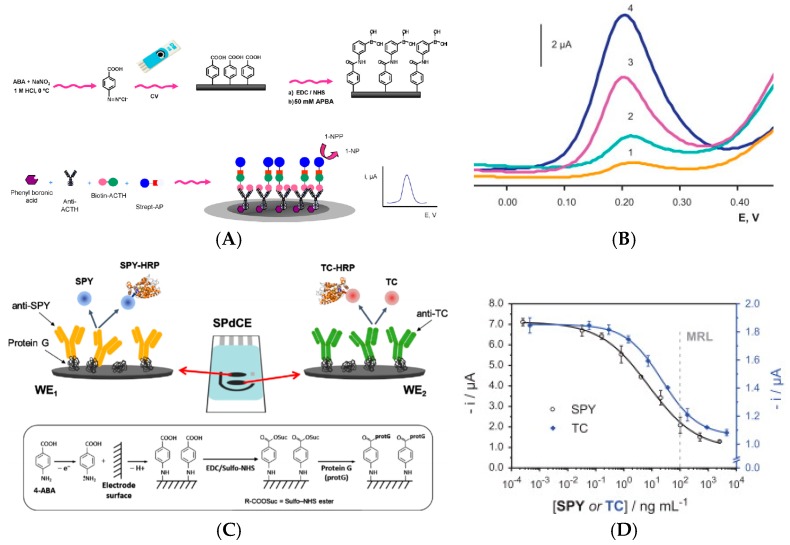
(**A**) Reactions involved in the ACTH immunosensor using SPEs modified with phenylboronic acid; (**B**) DPV obtained with the Strept-AlkP–Biotin-ACTH/anti-ACTH/SPCE electrode for 0 (1), 0.05 (2), 0.50 (3), and 1.00 (4) pg mL^−1^ ACTH. Reprinted from [16], Copyright 2012, with permission from Elsevier; (**C**) Above, scheme of the immunosensor principle for SA and TC antibiotics. Below, surface chemistry involved for covalent binding of Protein G; (**D**) Calibration curves obtained with the SPY/TC immunosensor for SPY and TC in a 1:1 PBS:milk mixture. Reprinted from [17], Copyright 2013, with permission from Elsevier.

Still in 2012, Moreno-Guzmán et al. [16] described a competitive electrochemical immunosensor for adrenocorticotropin hormone (ACTH) using disposable phenylboronic-modified carbon SPE used to efficiently immobilize ACTH antibodies. Transduction was designed using competition equilibrium for the binding sites of the immobilized antibody, between the target ACTH and a biotinylated ACTH (Figure 2A,B). The electroanalytical response was generated by using an AlkP-labelled streptavidin and 1-naphtyl phosphate as enzyme substrate. Differential pulse voltammetry (DPV) was used to monitor the enzyme activity (instead of classical CV, to suppress the capacitive component). A very low LoD of 18 fg mL^−1^ was obtained.

A competitive immunosensing approach was also followed by Conzuelo et al. [17] in 2013, for determination of sulfonamide (SA or SPY) and tetracycline (TC), two antibiotics which could be present in milk. The originality was to use Protein G coupled to 4-aminobenzoic acid electrografted on the electrode, as anchoring point for oriented immobilization of anti-SA and anti-TC (Figure 2C,D). Using HRP-labelled TC, they obtained a LoD of ca. 1 nM (ca. 200–500 pg mL^−1^) for both SA and TC. As for the previous ACTH competitive detection, no linear range was given, probably because it is relatively difficult to obtain such linear response with competitive transduction. For detection of cancer antigen 125 (CA-125), Singh et al. [18] described a Ru(NH_3_)_6_^3+^-mediated glucose oxidase (GOx) labelling instead of routinely used enzymes such as HRP or AlkP. The LoD, for an incubation period of 5 min, was slightly lower 0.1 U mL^−1^ for CA-125, comparable to the other reported electrochemical immunosensors. However, the authors claimed a shorter incubation time compared to HRP or AlkP amplifications.

More generally, for enzyme-based amplification routes, the turnover of the enzyme (more precisely the K_cat_/K_M_ ratio, which should be as high as possible) is a crucial parameter for a good amplification. Jiang et al. [19] described an electrochemical immunosensor for detection of tumor necrosis factor α (TNF-α) with a strategy to avoid non-specific adsorption. For this purpose, they used an original layer of phenylphosphoryl choline (PPC) and phenylbutyric acid (PBA). The capture antibody was grafted on the working ITO electrode along with this anti-adsorption layer and, in a sandwich configuration, the signaling (labelled) antibody was coupled to HRP. H_2_O_2_ was added in solution as well as ferrocene (Fc) to recycle HRP. The immunosensor was shown to detect TNF-α with a LoD of 10 pg mL^−1^ with a wide linear range between 0.01 ng mL^−1^ to 500 ng mL^−1^. More recently, Serafín et al. [20] reported in 2017 a tyrosine kinase immunosensor involving a sandwich architecture with a capture antibody covalently immobilized on poly(pyrrolepropionic acid)-modified electrodes and a HRP-labeled secondary antibody. The LoD was 337 pg mL^−1^.

Due to the relative instability, limited robustness and severe limitation of the operating conditions required for most enzymes, a strategy to get rid of enzymes is to design non-amplified sensors. However, for the sake of sensitivity, enzyme-free catalytically-amplified immunosensors should be considered as one of the most promising perspectives. These two approaches are reviewed below.

#### 3.1.2. Enzyme-Free Immunosensors

**Figure 3:**
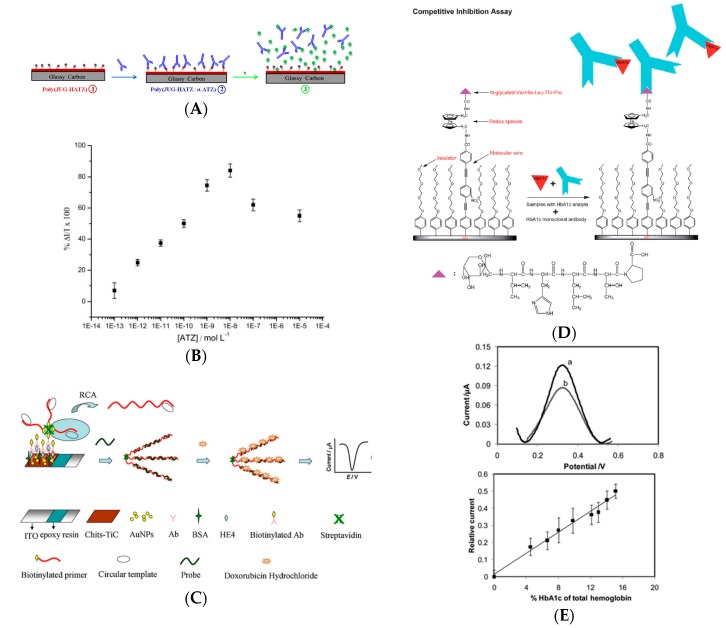
(**A**) Strategy for the electrochemical detection of atrazine based on the change in electroactivity of a polymer film poly(juglone-ATZ); (1) polymer/hapten-modified electrode; (2) after complexation with anti-ATZ; (3) after addition of ATZ in solution; (**B**) Calibration curve measured from SWV at −450 mV/SCE, after addition of ATZ from 1 pM up to 10 μM. Adapted from [22], Copyright 2012, with permission from Elsevier; (**C**) RCA-based immunosensor for HE4 detection. Reprinted from [26], Copyright 2012, with permission from Elsevier; (**D**) Scheme of the competitive inhibition assay for detecting HbA1c (anti-HbA1c: blue Y; HbA1c: red triangle; GPP: pink triangle, surface bound); (**E**) *Above*, a representative SWV for a FDMA-modified electrodes after (a) grafting of GPP and (b) incubation in 2 μg mL^−1^ anti-HbA1c and 13.5% HbA1c. *Below,* corresponding calibration curve. Reprinted from [23] with permission from The Royal Society of Chemistry.

Ciani et al. [21] developed gold SPE electrodes modified with specific thiolated antibodies for direct detection of infection biomarkers, using electrochemical impedance spectroscopy (EIS), more particularly measuring the charge transfer resistance of FeCN_6_^3−/4−^ on the gold electrode depending on the presence or not of the targeted antigen (TREM-1, MMP-9 and HSL). Limits of detection were between the pM and the nM range, depending on the target. Also with an enzyme-free transduction procedure, Tran et al. [22] described a label-free electrochemical competitive immunosensor based on an electroactive conducting polymer coupled with a molecule close to atrazine (a common pesticide) (Figure 3A,B). This quinone-based polymer presented a current decrease following anti-atrazine antibody complexation, and a current increase after atrazine addition in solution, with a very low detection limit of 1 pM, i.e., 0.2 pg mL^−1^ estimated by square wave voltammetry (SWV). One originality relies on the fact that the redox probe is not diffusing in solution but immobilized on the electrode surface, and another originality is the competitive equilibrium between an immobilized mimic of the target (so-called hapten) and the diffusing target to detect. Compared to many other examples in the literature, it should be noted that this non-amplified method gave an electroactivity increase upon recognition of the target.

The same year (2012), a similar idea was developed by Liu et al. [23] They reported an electrochemical immunosensor for detecting glycosylated hemoglobin (HbA1c) based on glassy carbon (GC) electrodes modified with a mixed layer of oligo(phenylethynylene) and oligo(ethyleneglycol), obtained by electrografting of the corresponding aryldiazonium salts.

1,1′-Di(aminomethyl)ferrocene and an epitope a pentapeptide, glycosylated-VHLTP (GPP) were covalently attached to oligo(phenylethynylene) (GPP is a peptide mimetic to HbA1c, to which an anti-HbA1c antibody could bind). As for Tran et al. HbA1c was detected by a competitive assay based on the competition for binding to anti-HbA1c between the analyte in solution, HbA1c, and the surface bound GPP peptide. However, exposure of the GPP-modified interface to the mixture of anti-HbA1c IgG antibody and HbA1c resulted in the attenuation of Fc electroactivity due to steric hindrance generated by the antibody bound to the surface (Figure 3D,E), and not to an increase in electroactivity as reported by Tran et al. The authors found that HbA1c could be detected from 4.5% to 15.1% of total hemoglobin in serum. The same authors, the same year, adapted this method to AuNPs-modified surfaces (reference cited later in the text). Still in order to avoid addition of a diffusing redox probe in solution, Wang et al. [24] reported later a similar approach, based on an electroactive polymer onto which an antibody was coupled, to detect bisphenol A (BPA) by competitive binding assay with a detection limit of 2 pg mL^−1^ using SWV. A current decrease was obtained upon anti-BPA binding and an opposite current increase upon BPA addition in solution. The same authors described a similar approach for detection of acetaminophen. [25] The detection limit was ca. 10 pM (1.5 pg mL^−1^). These approaches present the great advantage to use a simple design, with few reactants, all immobilized on the sensing electrode.

However, other more complicated design could also perform well. One of them, using DNA, was reported by Lu et al. [26]. They described detection of human epididymis-specific protein 4 (HE4) with a chitosan–titanium carbide-modified ITO electrode (Chi-TiC/ITO) onto which AuNPs were deposited. The capture antibody was adsorbed onto the Au and TiC NPs. For transduction and amplification, secondary antibodies were labelled with DNA strands, followed by rolling circle amplification (RCA). Using doxorubicin as DNA intercalator and DPV for detection, the redox current responded to HE4 linearly in the concentration range of 3–300 pM, with a LoD of 0.06 pM (respectively 3–300 ng L^−1^ and 0.06 pg mL^−1^) (Figure 3C). This is a good example of enzyme-free amplification where the authors tried to increase the surface density of the redox probe by multiple intercalation within the DNA strands. The high surface density of doxorubicin achieved by this strategy provided high currents, so high sensitivity.

These approaches could appear very complicated. For this reason, Electrochemical Impedance Spectroscopy (EIS) combined with a diffusing redox probe stayed popular. Hayat et al. [27] described the immobilization of anti-okadaic acid antibody on 4-carboxyphenyl film. The Ab/Ag binding was transduced simply using electrochemical impedance spectroscopy with FeCN_6_^3−/4−^ as diffusing redox probe. The increase in electron transfer resistance was linearly proportional to the okadaic acid concentration in the range 0.195–12.5 μg L^−1^, with a LoD of 0.3 ng mL^−1^. In 2013, Vasudev et al. [28] described a similar procedure for epidermal growth factor receptor (EGFR) detection, by immobilizing anti-EGFR antibody on dithiobissuccinimidyl propionate (DTSP) SAM on Au electrodes. EIS measures with Fe(CN)_6_^3−/4−^ exhibited a linear range from 1 pg mL^−1^ to 100 ng mL^−1^ and a LoD of 1 pg mL^−1^. Vasudev et al. [29] also presented the same procedure but replaced conventional Au electrode by microfabricated interdigitated ones. As a proof-of-concept, cortisol antibodies were immobilized using the same SAM as previously described. Cortisol (MW 362 g mol^−1^) was detected using CV over a linear range of 10 pM to 100 nM (3.6 pg mL^−1^–36 ng mL^−1^).

This example is probably the occasion to recall here that the relative size of the target molecule compared to that of an antibody should determine the choice of the transduction architecture of an electrochemical immunosensor. Indeed, the most popular Ab reported in biosensors are immunoglobulins G (IgG), with a typical molecular weight of 150 kDa, i.e., a volume of between 300 and 700 nm^3^) or a projected area of ca. 60 nm^2^. This should be compared to the molecular weight of the target antigen. If this one is a for example a small protein of 30 kDa, it corresponds to a projected area of 22 nm^2^, i.e., ca 30% of the antibody’s. But for a molecule (such as pesticide, industrial or pharmaceutical pollutant) of 200 g mol^−1^, this ratio falls to 1% of the antibody’s projected surface, which is negligible and cannot play a significant role in changing the steric hindrance at the solution/electrode interface; for such situations, other transduction schemes should be considered.

It is possible to go beyond this steric hindrance limitation and play on electrostatic repulsions rather than on the size of the target. For detection of a bulky protein (porcine serum albumin), Lim et al. [30] reported a carbon nanofiber-modified SPE electrofunctionalized with a 4-carboxyphenyl diazonium salt onto which antibodies were covalently bound. Taking profit of the strong affinity of serum albumins towards anions, an anionic redox probe was used in solution. An increase in cathodic peak current was measured after immunocomplex formation between antibodies and proteins. The linear range was from 0.5 to 500 pg mL^−1^ and the LoD of 0.5 pg mL^−1^.

**Figure 4:**
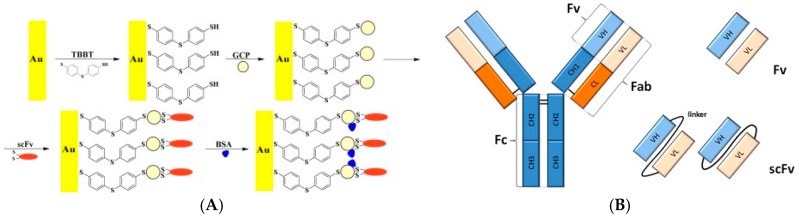
(**A**) Hemagglutinin H5 immunosensor based on a SAM of 4,4′-thiobisbenzenethiol carrying single chain variable fragments (scFv) of antibodies as probes. Reprinted from [31], Copyright 2016, with permission from Elsevier; (**B**) Schematic representation of a scFv fragment. A scFv fragment makes 25 kDa and corresponds to the VH + VL domains; it is the smallest fragment that holds a complete binding site of an antibody and therefore provides its specificity. From Antibody Design Laboratories (http://www.abdesignlabs.com/technical-resources/scfv-cloning/); access 29 January 2017.

Reducing the size of the capture antibody by using only Ab fragments or an analog of protein (a peptidomimetic) is also a solution. Using Fe(CN)_6_^3−/4−^ as an electroactive diffusing probe, Jarocka et al. [31] reported detection of hemagglutinin from avian influenza virus H5N1. Gold electrodes were modified with a SAM of 4,4′-thiobisbenzenethiol (TBBT), itself modified by gold nanoparticles (AuNPs) and single chain variable fragments of antibodies (scFv) against hemagglutinin H5 (Figure 4A). Interactions between the fragment of antibodies and hemagglutinin were sensed by EIS, giving a LoD of 0.6 pg mL^−1^ and a linear range from 4.0 to 20.0 pg mL^−1^. This fragment makes 25 kDa and corresponds to the variable domains; it is the smallest fragment that holds a complete binding site of an antibody and therefore keeps its specificity (Figure 4B). Figures of merit discussed in this section are summarized in Table 2.

### 3.2. Nanostructured Carbon Substrates

As shown above, one of the best way to increase sensitivity is to increase the specific area of the probe-modified surface, which increases contacts with the analyte in solution. For this purpose, nanomaterials have been widely investigated these last years.

#### 3.2.1. Carbon Nanotubes

• Enzyme-Based Immunosensors

**Figure 5:**
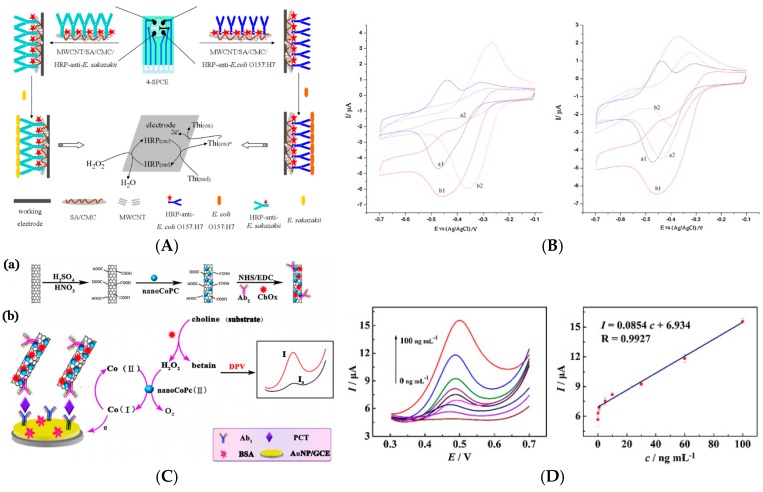
(**A**) Preparation of the immunoelectrode, and reactions occurring at the electrode surface for determination of E. sakazakii and E. coli O157:H7. Thi(ox) and Thi(red) are the oxidized and reduced forms of thionine, respectively; (**B**) CVs after incubation with *E. sakazakii* (10^10^ cfu mL^−1^) (left) and *E. coli* O157:H7 (right). Curves a1 and b1 were obtained before incubation; curves a2 and b2 were obtained after incubation. Reprinted from [32], Copyright 2013, with permission from Elsevier; (**C**) (**a**) Preparation procedure of the ChOx/Ab_2_/CoPc-MWCNTs bioconjugates; (**b**) Transduction and amplification mechanisms; (**D**) DPV responses after incubation with PCT (from 0.01 to 100 ng mL^−1^), and corresponding calibration curve of the anodic peak current. Reprinted from [38], Copyright 2016, with permission from Elsevier.

In 2013, Dou et al. [32] described an electrochemical immunosensor for enterobacterial detection using a carbon SPE modified by multi-walled carbon nanotubes (MWCNTs)/alginate/chitosan composite onto which HRP-labeled capture antibodies were immobilized. Detection of the Ag/Ab binding was transduced using CV with thionine (Thi) and H_2_O_2_ diffusing in solution, without the use of any secondary antibody (Figure 5A,B). They found a linear range from 10^4^ to 10^10^ CFU mL^−1^ and a LoD of 5 × 10^3^ CFU mL^−1^.

Gomes-Filho et al. [33] reported the use of conventional oxidized CNTs immobilized on polyethyleneimine to bind anti-cTnT (cardiac troponin T, a diagnostic biomarker for myocardial infarction or heart muscle cell death) capture antibodies. After a conventional sandwich assay with a HRP-labelled anti-cTnT and upon addition of H_2_O_2_ in solution, they measured the cathodic peak current of Fe(CN)_6_^3−/4−^ also added in solution and achieved a LoD of 0.033 ng mL^−1^ and a linear range between 0.1 and 10 ng mL^−1^. In 2016, still using a diffusing redox probe and not an immobilized one, Zhang et al. [34] described an electrochemical immunosensor for detection of a mycotoxin (aflatoxin B_1_) based on SWCNT/chitosan electrodes, using a conventional indirect competitive binding with an AlkP-labelled secondary IgG antibody. AlkP was used to catalyze the hydrolysis of α-naphthyl phosphate (added in solution), which in turn produced an electrochemical signal at the electrode. Using DPV, they found a linear response between 0.01 and 100 ng mL^−1^, with a LoD of 3.5 pg mL^−1^ for aflatoxin B_1_. At last for immunoassays using diffusing redox probes, Sánchez-Tirado et al. [35] reported in 2016 an electrochemical immunosensor for the determination of Transforming Growth Factor β1 cytokine using MWCNT-modified SPE. MWCNTs were functionalized by azide–alkyne click chemistry for covalent coupling of alkyne-functionalized anti-TGF. The target was detected through sandwich immunoassay with HRP-labeled anti-TGF. The affinity reaction was monitored amperometrically at −0.20 V using the HQ/H_2_O_2_ system. Linearity was obtained between 5 and 200 pg mL^−1^ and the LoD was 1.3 pg mL^−1^.

It could be more pertinent not to add diffusing redox probes in solution, for the sake of simplicity, reproducibility, or simply to make the sensors compatible with in-situ measurements for which redox mediators could not be added to the analyzed medium. This is what Salimi et al. [36] reported using thionine as redox probe integrated on the electrode substrate. They developed a sensor for detection of prostate specific antigen (PSA) based on immobilization of a PSA antibody using a composite of MWCNTs and an ionic liquid (1-butyl-methylpyrrolidinium bis(trifluromethyl- sulfonyl) imide, [C_4_mpyr][NTf_2_]). Anti-PSA was immobilized in this composite as well as thionine (Thi), and HRP-labeled anti-PSA was used in a sandwich type immunoassay with H_2_O_2_ as substrate. Using DPV to oxidize Thi at the electrode, the linear range was between 1 and 40 ng mL^−1^, with a LoD of 20 pg mL^−1^. More recently, Wang et al. [37] reported a poly-L-lysine/SWCNT-modified electrode coated with Prussian blue (BP) for α-fetoprotein detection. Poly-L-lysine is a positively charged synthetic polymer of L-lysine containing amino groups able to bind bioactive materials onto an electrode surface, or to link some active groups such as epoxy groups, hydroxyl groups, or carboxyl groups present on the SWCNTs surface. All these components were crosslinked with glutaraldehyde to stabilize the interface. Immunosensing was measured based on the catalytic activity of the HRP with H_2_O_2_ added in solution and BP acting as mediator immobilized on the electrode surface. Using DPV to recycle Prussian Blue, the authors found that peak current was linearly related to α-fetoprotein in the range 0.05–10.0 ng mL^−1^, with a LoD of 10 pg mL^−1^. In the same spirit, Yang et al. [38] described cobalt phthalocyanine (CoPc)-functionalized MWCNTs as label for signaling antibodies (Ab_2_) in a sandwich-type immunosensor, for detection of procalcitonin (a peptide biomarker of severe sepsis). The originality and pertinence of this approach was that the electrochemical signal directly originates from the CoPc without the addition or any redox mediator in solution or any label, which makes such kind of tranuduction scheme more efficient for a real application. However, to enhance sensitivity, choline oxidase (ChOx) was added on the electrode. H_2_O_2_ produced by this enzyme was catalytically oxidized by CoPc, resulting in a signal amplification (Figure 5C,D). Using DPV, they reported linearity from 0.01 to 100 ng mL^−1^ and a LoD of 1 pg mL^−1^, but choline had to be added in solution, which did not make this system reactant-free.

• Enzyme-Free Immunosensors

**Figure 6:**
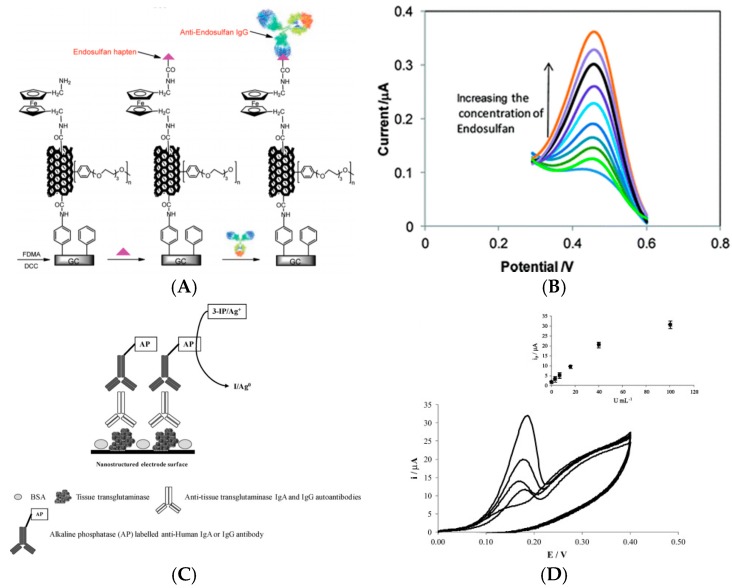
(**A**) SWNT-modified GC electrodes for detection of endosulfan; (**B**) SWVs for GC-Ph-NH_2_/SWNT/PEG/FDMA/endosulfan/anti-endosulfan electrode after incubation in with 0, 0.02, 0.05, 0.1, 0.2, 0.4, 0.8, 1, 2, and 4 ppb endosulfan, respectively. Adapted with permission from [40]. Copyright 2012 American Chemical Society; (**C**) Immunosensing architecture used for tissue transglutaminase. 3-IP: 3-indoxyl phosphate disodium salt; (**D**) CVs obtained for anti-tTG at various concentrations. *Inset*: relationship between the peak current and the anti-tTG concentration. Reprinted from [42], Copyright 2012, with permission from Elsevier.

For enzyme-free detection of aflatoxin-B_1_, Singh et al. [39] reported a method of functionalization of an ITO electrode by electrophoretic deposition of MWCNTs (lying flat on the electrode surface) onto which aflatoxin antibodies were grafted. Using Fe(CN)_6_^3−/4−^ as redox probe diffusing in solution and CV as electrochemical technique, their immunosensor was sensitive to the association of the aflatoxin-B1 on the immobilized Ab, showing a LoD of 0.08 ng mL^−1^ and a linear detection range between 0.25 and 1.4 ng mL^−1^. The most interesting example, however, was given by Liu et al. [40] in 2012. They reported a sensor for detection of endosulfan (an organochlorine insecticide) by electrografting of a mixed layer of 4-aminophenyl and phenyl with the aryldiazonium route, for covalent grafting of SWCNTs on conventional GC electrodes. They also used the diazonium electrografting route for functionalization of these SWCNTs with anti-adsorption chains such as poly(ethylene glycol) (PEG). Ferrocenedimethylamine was attached to the upper end of SWNTs through amide bonding followed by the attachment of the endosulfan hapten to which an antibody bind (Figure 6A,B). Association/dissociation of the antibody on the sensing interface causes a modulation of the ferrocene electroactivity. SWV was used to sense this electroactivity change, with a linear detection range of ca. 0.01–20 pg mL^−1^. It must be emphasized that, through this competitive assay, without the use of any diffusing redox probe in solution nor additional reactant, they achieved a excellent LoD of 0.01 pg mL^−1^; this approach certainly paved the way for enzyme-free, reactant-free, diffusion-free immunosensors.

#### 3.2.2. Nanoparticles Combined with Carbon Nanotubes

• Enzyme-Based Immunosensors

Because CNTs are difficult to functionalize, or poorly conducting after some oxidative treatments, and also to bring more efficient and more specific catalytic properties to the electrode, some works were reported where CNTs were modified with metal NPs. For example, Lu et al. [41] reported an electrochemical immunosensor based on MWCNTs modified with Au nanoparticles (AuNPs) for detection of human chorionic gonadotrophin (hCG), widely used as a marker in some pregnancy test. AuNPs were used to increase further the surface area of the electrode to track down a large amount of capture antibodies as well as to lower the electronic transfer resistance of generated by poorly conductive CNTs. HRP-labeled secondary anti-hCG antibodies were used for the sandwich immunoassay. Linearity was obtained from 5 μIU mL^−1^ to 500 mIU mL^−1^ with a LoD of 3 μIU mL^−1^. A similar approach was described by Neves et al. [42] for detection of IgA- and IgG-type anti-tissue transglutaminase (anti-tTG) autoantibodies in real samples from patients suffering with celiac disease, using MWCNT/AuNPs as substrate and AlkP-labeled secondary anti-IgG antibodies for amplification. The analytical signal was based on the anodic redissolution of the enzymatically generated silver from Ag^+^ added in solution (Figure 6C). No quantification and even no detection limit were claimed, but positive (+) or negative (−) results, for real diluted serum samples. The fact that silver must be added in solution impede the use of such sensor for real applications, however.

• Enzyme-Free Immunosensors

Only one example of enzyme-free immunosensor could be found during this period, very recently published by Liu et al. in 2017. [43] They described a label-free amperometric immunosensor for the direct determination of zearalenone (a mycotoxin). GC electrodes were modified with polyethyleneimine (PEI)-functionalized MWCNTs, then AuPtNPs were electro-deposited. They demonstrated that AuPtNPs increased the surface concentration of antibodies and enhanced sensitivity. Capture monoclonal antibodies were immobilized on these NPs. Using CV with Fe(CN)_6_^3−/4−^ as redox probe (diffusing in solution), they probed the target capture over a wide linear range from 5 pg mL^−1^ to 50 ng mL^−1^, with a LoD of 1.5 pg mL^−1^.

#### 3.2.3. Other Carbon Materials

CNTs are not the only carbon-based nanoparticles. For example, Xu et al. [44] reported an immunosensor using carbon nanospheres and AuNPs, which were used for labeling secondary antibodies in a sandwich-type immunoassay format. For transduction, these AuNPs were electro-oxidized to produce AuCl_4_^−^ at the electrode surface, detected by DPV. The high-loading capability of AuNPs on carbon nanospheres led to obvious signal amplification. Using human immunoglobulin G (IgG) as model target, they obtained a linear dependence on the logarithm of target concentration ranging from 10 pg mL^−1^ to 10 ng mL^−1^, with a LoD of ca. 10 pg mL^−1^. No external reactants were necessary.

**Figure 7:**
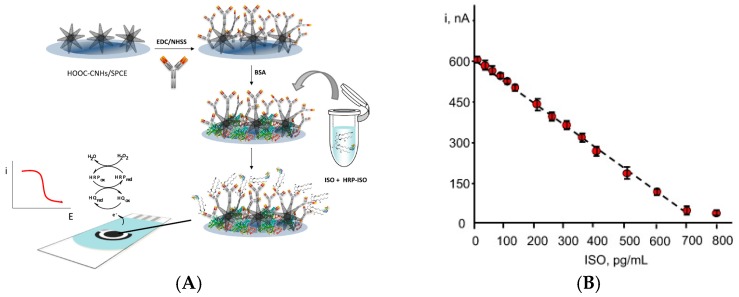

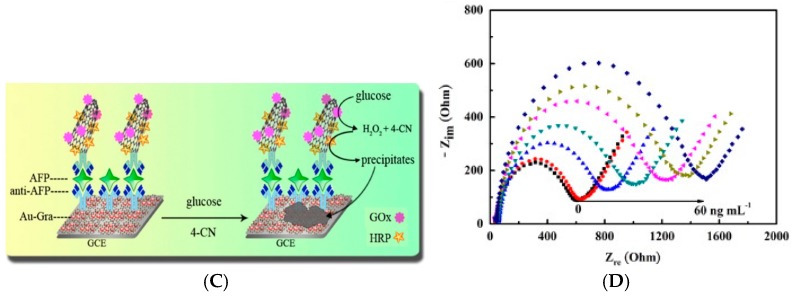
(**A**) Functioning of the 8-isoprostane immunosensor on a SPE; (**B**) Corresponding calibration plot for 8-isoprostane. Adapted from [45], Copyright 2016, with permission from Elsevier; (**C**) Schematic representation of electrode modification by the nanohorns–bienzyme–Ab_2_ complex, for AFP detection; (**D**) Nyquist diagrams of bienzyme/Ab_2_/SWCNHs bioconjugates, for various AFP concentrations. Adapted from [46], Copyright 2014, with permission from Elsevier.

Using carbon nanohorns instead of nanospheres, Sanchez-Tirado et al. [45] reported a sensor for determination of 8-isoprostane, a biomarkers of lipid peroxidation in the human body, derived of essential fatty acids (Figure 7A,B). A competitive immunoassay involving HRP-labeled 8-isoprostane was designed and detection was based on the steric hindrance generated by bound 8-isoprostane on the electrode surface, expected to impede diffusion of the redox species (i.e., H_2_O and BQ). A linear response was obtained up to 700 pg mL^−1^ with a LoD of 12 pg mL^−1^.

**Figure 8:**
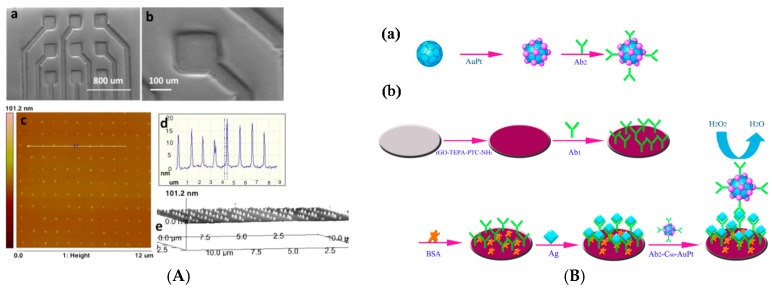
(**A**) Biosensor chip for CRP, cTnT and myoglobin; (**a**) SEM image of an array of 9 electrodes, and (**b**) of an individual electrode; (**c**) AFM image of an array carbon nanofibers (bright dots); (**d**) AFM profile of the carbon nanofibers height; (**e**) 3D AFM micrograph after antibody immobilization. Reprinted from [47], Copyright 2016, with permission from Elsevier; (**B**) (**a**) Ab_2_–C_60_–AuPtNPs and (**b**) scheme of the Vangl1 immunosensor using these C_60_ templates. Reprinted from [48], Copyright 2016, with permission from Elsevier.

Another procedure, which guaranties diffusion hindering (then efficient transduction) independently of the size of the targets and probes, was interestingly reported by Yang et al. [46] who described detection of α-fetoprotein (AFP) with the use of single-walled carbon nanohorns. Based on a sandwich-type immunoreaction, a bienzymatic (HRP and GOx) cascade was used for transduction and amplification (Figure 7C,D). 4-Chloro-1-naphthol, used a redox cosubstrate for HRP, was catalytically oxidized by H_2_O_2_ to yield to an insoluble product on the electrode surface, which was probed with Fe(CN)_6_^4−/3−^ using CV and EIS. The sensor showed a wide linear range from 1 pg mL^−1^ to 60 ng mL^−1^ and a LoD of 0.33 pg mL^−1^. Recently (2016), Gupta et al. [47] showed that such nanocarbon structured could be featured into integrated devices. Indeed, they described a multiplexed electrochemical immunosensor for label-free detection of three cardiac markers (C-reactive protein, cardiac troponin-I and myoglobin) using carbon nanofibers as electrodes. Carbon nanofibers were grown vertically using plasma enhanced chemical vapor deposition (PE-CVD), onto which capture antibodies were coupled by conventional carbodiimide chemistry. The complexation of the cardiac markers on the corresponding antibodies were characterized using Fe(CN)_6_^3−/4−^ as redox probe (Figure 8A). No detection limits were given but this device finds its originality in the nanofibers displayed in a relatively dense array (however not electrochemically addressable individually), providing a very high surface area for Ab immobilization.

At last, Chen et al. [48] reported C_60_-templated AuPtNPs for human Vang-like protein (Vangl1) detection (a biomarker for dysontogenic diagnostic). These C_60_/AuPtNPs were used for signal amplification and immobilization of the signaling antibodies, whereas reduced graphene oxide (RGO) was used to immobilized capture antibodies on the working electrode. An electrochemical signal was derived from the catalytic reduction of H_2_O_2_ by C_60_–AuPt (Figure 8B). The authors observed a linear range from 0.1 pg mL^−1^ to 450 pg mL^−1^ and a LoD of 0.03 pg mL^−1^.

As shown, it is generally assumed that transduction should be based whether on the steric effect of antibodies or on the use of labeled secondary antibodies; most architectures needed addition of a reactant in solution, which could be satisfactory for laboratory proof-of-concept but seem unusable in real conditions. Figures of merit discussed in this section are summarized in Table 3.

### 3.3. Graphene and Graphene Derivatives

Graphene offers, among other features, large surface area and high electrical conductivity. It has been widely used in electrochemical sensor devices in which it participates to increase current densities and contact area capture probe and target molecules. The advantage that graphene (but not graphene oide) could have compared to CNTs or related carbon NPs is its intrinsic conductivity as well as, if correctly prepared (that is, in a well-dispersed form and not as bundles) a higher surface-to-volume ratio.

#### 3.3.1. Enzyme-Free Immunosensors

As for classical substrates, most of the graphene-based immunosensors concern detection of antigens; however, some immunosensors were described for antibody detection. All of them proposed transduction architecture strictly similar to those used to detect antigens. In 2012, Loo et al. [49] reported a conventional label-free electrochemical impedimetric immunosensor for detection of IgG antibodies as model, based on chemically modified graphene serving as electrode substrate. Anti-IgG were immobilized on the electrodes and EIS used with FeCN_6_^3−/4−^ as diffusing redox molecule for probing Ab/Ag interaction on the electrode surface. The linear range of detection was from 0.3 μg mL^−1^ to 7 μg mL^−1^ (LoD of 0.1 μg mL^−1^). In 2013, Wang et al. [50] presented a sandwich electrochemical immunosensing strategy with AuNP-functionalized graphene, also used as immobilization substrate for probe antibodies, and 1,1′-ferrocenedicarboxylic acid as label on the signaling antibodies. Human IgG was detected using DPV with a LoD of 0.4 ng mL^−1^ and a linear dynamic range from 1 to 300 ng mL^−1^, that is much lower values than those obtained from the previous example, with a sensor that does not need a redox label to be added in solution. More recently, Zhang et al. [51] reported an IgG immunosensor based on AuNPs/polydopamine-functionalized RGO as substrate for probe antibodies, and AgNPs/carbon dots (C-dots) as signal probe and catalytic material onto which signaling antibodies were immobilized. The presence of the IgG target was detected by monitoring the electroreduction current of BQ coming from the reaction of H_2_O_2_ and HQ (both added in solution) on the AgNPs/C-dots. The current responses were linear between 0.01 and 100 ng mL^−1^, with a LoD of 1 pg mL^−1^. There are more examples of immunosensors for antigen detection. For this reason, we sorted them between sensors needing diffusing redox probes and the ones, more interesting, which use immobilized redox probes without needing added reactant.

• Diffusing Redox Probes

**Figure 9:**
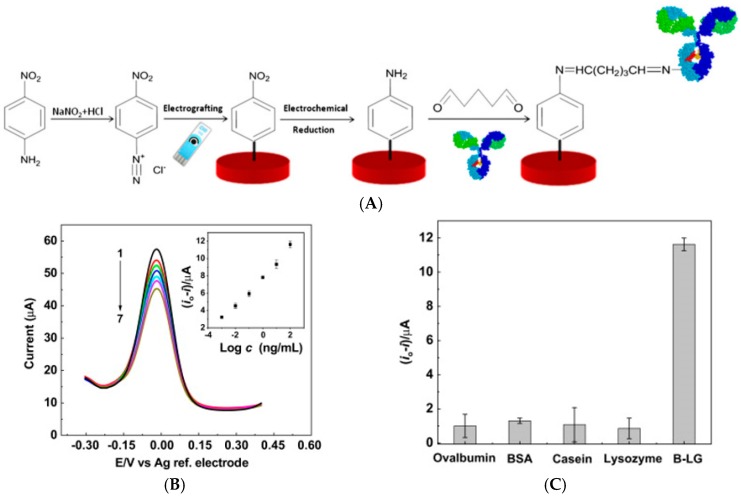
(**A**) Electroreduction of a diazonium salt on a SPE, for covalent immobilization of β-lactoglobulin antibodies; (**B**) DPVs of the immunosensor incubated with different concentrations of β-lactoglobulin (inset: calibration curve); (**C**) Comparison of the DPV response of the graphene-modified electrodes to 1000 ng mL^−1^ ovalbumin, BSA, casein, lysozyme, and 100 ng mL^−1^ β-lactoglobulin. Reprinted from [52], Copyright 2012, with permission from Elsevier.

Eissa et al. [52] described in 2012 an immunosensor for β-lactoglobulin (a milk antigen) using graphene-modified SPE. They demonstrated electrografting of an aryl diazonium salt on graphene as substrate, for covalent grafting of the β-lactoglobulin capture antibodies through a Schiff base reaction (Figure 9). CV and DPV were carried out using Fe(CN)_6_^3−/4−^ added in solution to characterize Ab/Ag association. Currents decreased linearly with increasing the concentration of β-lactoglobulin due to the steric hindrance generated by the antibody–antigen complex on the modified electrode surface. With this classical transduction scheme, the LoD was 0.85 pg mL^−1^ and the dynamic range was from 1 pg mL^−1^ to 100 ng mL^−1^.

In 2013, Zhao et al. [53] reported an enzyme-free immunosensor for detection of α-fetoprotein (AFP), using a sandwich-like format with catalytic AuPdNPs-labeled antibodies and *N*-doped graphene for grafting of the capture antibody on the electrode. AuPdNPs were used for their catalytic properties towards H_2_O_2_ added in solution. The LoD was 5 pg mL^−1^ and the linear range between 0.05 and 30 ng mL^−1^. Still in 2013, Huang et al. [54] also described a label-free amperometric immunosensor for AFP detection, based on a TiO_2_/graphene/chitosan/AuNPs film deposited on a GC electrode. Antibodies were immobilized by adsorption on the AuNPs. The Ab/Ag interaction at the electrode interface generated a steric hindrance, resulting in the decrease of DPV signals when Fe(CN)_6_^3 −/4 −^ was added in solution. The detection range was between 0.1 and 300 ng mL^−1^ and the LoD of 0.03 ng mL^−1^. Again, for AFP detection, Lin et al. [55] inserted anti-AFP and SWCNTs inside the channels of mesoporous silica (MPS) and immobilized this MPS/SWCNTs/anti-AFP assembly on graphene using a layer-by-layer approach. Capture of AFP was probed by DPV using ferrocenecarboxylic acid diffusing in solution. The authors reported detection of AFP within a linear range from 0.1 to 100 ng mL^−1^ and a LoD of 0.06 ng mL^−1^. In 2015, Liu et al. [56] described a GR/SnO_2_/Au nanocomposite used to immobilize anti-AFP. Using Ru(NH_3_)_6_^3+^ as redox probe added in solution, the authors demonstrated that the DPV peak currents decreased due to the interaction between Ab and Ag on the electrode. AFP was quantified between 0.02 and 50 ng mL^−1^, with a LoD of 0.01 ng mL^−1^. In 2016, last example for AFP detection, Wei et al. [57] presented a sandwich-type electrochemical immunosensor where AuNPs were electrodeposited on GCE to bind capture anti-AFP. Graphene oxide functionalized with CeO_2_NPs and PdNPs was utilized as labels for labelling secondary anti-AFP. PdNPs played the role of catalyst towards H_2_O_2_ reduction. The immunosensor exhibited a linear range from 0.1 pg mL^−1^ to 50 ng mL^−1^ with a LoD of 0.033 pg mL^−1^.

**Figure 10:**
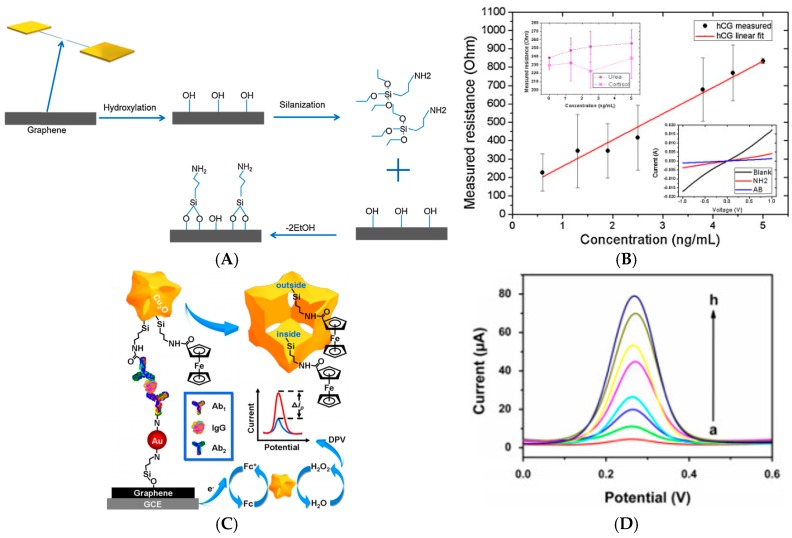
(**A**) Covalent attachment of 3-aminopropyltriethoxysilane (APTES) to a hydroxyl-terminated graphene surface; (**B**) Resistance across a 100 μm × 4 mm graphene channel as a function of the hCG concentration. Resistance across the channel as a function of the urea and cortisol concentration (inset, top left). I–V curves are plotted (inset, bottom right) for the unmodified device (black curve), amine-terminated (red curve), and Ab-modified (blue curve) graphene surface. Reprinted from [59], Copyright 2014, with permission from Elsevier. (**C**) Sandwich-type electrochemical immunosensor based of Fc-modified Cu_2_ONPs, for PSA detection. (**D**) SWVs for detection of 0.05 pg mL^−1^ (a), 0.1 pg mL^−1^ (b), 0.5 pg mL^−1^ (c), 1 pg mL^−1^ (d), 5 pg mL^−1^ (e), 10 pg mL^−1^ (f), 50 pg mL^−1^ (g) and 100 pg mL^−1^ (h) PSA. Reprinted from [62], Copyright 2016, with permission from Elsevier.

Back in 2013, Li et al. [58] reported label-free detection of carbohydrate antigen 15-3 (CA 15-3), a biomarker of breast cancer. Capture antibodies were immobilized on N-doped graphene-modified electrodes. Upon recognition of the carbohydrate antigen and using DPV to probe the electroactivity of Fe(CN_6_)^3−/4−^ added in solution, a LoD of 12 mU mL^−1^ was achieved, and a linear range between 0.1 and 20 U mL^−1^. In 2014, Teixeira et al. [59] reported an immunosensor for human chorionic gonadotropin (hCG), a key hormone for pregnancy diagnostic. Multi-layer epitaxial graphene (MEG) was grown on SiC substrates, then patterned using electron beam lithography to make channels. These short channels were then functionalized with 3-aminopropyltriethoxysilane (APTES) to covalently bind the anti-hCG antibody (Figure 10A,B). Detection was made by monitoring the redox current of diffusing Fe(CN_6_)^3−/4−^ on the graphene sheet, or by monitoring changes in the channel resistance upon exposure to hCG. The LoD was 0.62 ng mL^−1^ and the linear response was in the range 0.6–6 ng mL^−1^.

**Figure 11:**
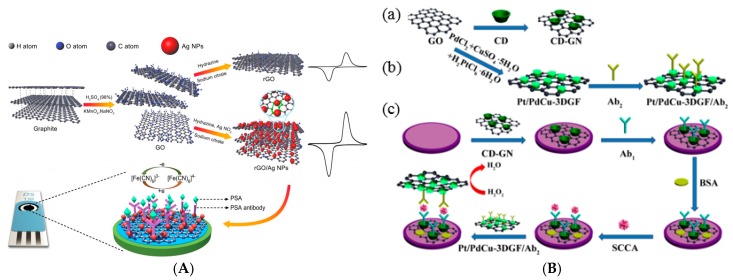
(**A**) RGO and RGO/AgNPs for PSA detection, with Fe(CN)_6_^3−/4−^ as diffusing redox probe. Reprinted from [63], Copyright 2017, with permission from Elsevier; (**B**) Cyclodextrin-modified graphene (**a**); functionalization by Pt/PdCuNPs and Ab_2_ (**b**); and (**c**) global immunosensing scheme. Reprinted from [65], Copyright 2016, with permission from Elsevier.

In 2015, Jang et al. [60] used AuNPs-modified graphene to bind PSA antibodies. CV was used with Fe(CN_6_)^3−/4−^ as diffusing redox probe to quantify PSA binding on immobilized anti-PSA. The LoD was 0.6 ng mL^−1^ and the linear range was obtained for up to 10 ng mL^−1^. Also in 2015, Sun et al. [61] described an original paper substrate modified by reduced graphene oxide (RGO), ZnO nanorods and capture antibodies. They used a sandwich assay where the signaling antibodies were coupled to graphene sheets and AgNPs. With H_2_O_2_ as redox probe and CV for measuring H_2_O_2_ electroreduction, the authors demonstrated a linear response between 1 pg mL^−1^ and 110 ng mL^−1^. This sensor was also employed for human chorionic gonadotropin (linearity between 2 μIU mL^−1^ and 120 mIU mL^−1^) and to carcinoembryonic antigen (CEA) (linearity between 1 pg mL^−1^ and 100 ng mL^−1^). In 2016, Ma et al. [62] also reported PSA detection but for a sandwich-type device. AuNPs-decorated APTES-functionalized graphene sheets (Au/APTES/GR) were employed as substrate, and Cu_2_O NPs decorated with ferrocene were employed as label. Cu_2_ONPs presented a good electrocatalytic activity towards Fc and hydrogen peroxide (added in solution) electrooxidation and electroreduction, respectively (Figure 10C,D). The linear range was between 0.05 and 100 pg mL^−1^ and the LoD of 0.05 pg mL^−1^. Last example of PSA detection, Han et al. [63] reported in 2017 AgNPs-functionalized RGO for immobilization of anti-PSA antibodies. Using CV with FeCN_6_^3−/4−^ as diffusing redox probe (Figure 11A), the authors demonstrated peak current changes which varied linearly with PSA concentration between 1 and 1000 ng mL^−1^, with a LoD of 0.01 ng mL^−1^.

Also using Fe(CN_6_)^3−/4−^ as redox probe, Pandey et al. [64] reported RGO sheets decorated by cysteine and CuONPs deposited on Au electrode, for detection of *Escherichia coli* O157: H7. With EIS to measure changes in electron transfer resistance, they found a detection range from 10 CFU mL^−1^ to 10^8^ CFU mL^−1^ and a LoD of 3.8 CFU mL^−1^.

Liu et al. [65] described a sandwich-type electrochemical immunosensor for detection of squamous cell carcinoma antigen (SCCA). Graphene nanosheets were immobilized on the electrode surface and functionalized with β-cyclodextrin, used to immobilize capture antibodies. Signaling antibodies were modified with Pt/PdCu nanocubes anchored on graphene sheets. Pt/PdCu exhibited high electrocatalytic activity toward the reduction of H_2_O_2_ added in solution (Figure 11B). The results showed linearity between 0.1 pg mL^−1^ and 1 ng mL^−1^, with a LoD of 25 fg mL^−1^.

**Figure 12:**
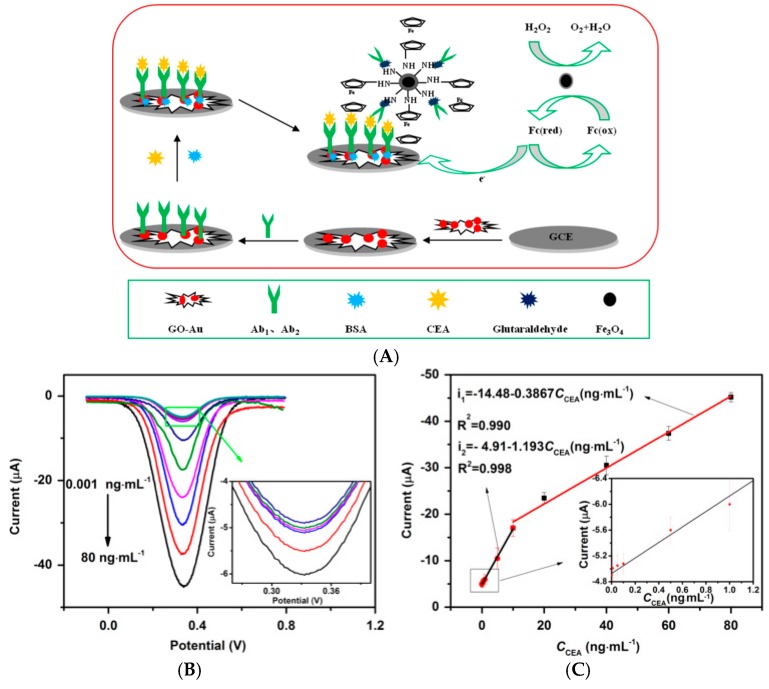
(**A**) Fe_3_O_4_@SiO_2_–Fc–Ab_2_/HRP particles for enzyme-less catalytic immunodetection of CEA. (**B**) DPV curves of the above-described sensor for various CEA concentrations in PBS + 4 mM H_2_O_2_; (**C**) *Calibration plots.* Adapted from [67], Copyright 2016, with permission from Elsevier.

Pd/V_2_O_5_ also presents a catalytic activity towards H_2_O_2_ reduction, demonstrated by Han et al. [66], in 2016, for a non-enzymatic sandwich-type immunosensor for carcinoembryonic antigen (CEA) detection. Pd/V_2_O_5_ was immobilized on MWCNT-labelled secondary antibodies. A nanocomposite of SnO_2_/RGO was used as high specific surface area substrate for immobilizing AuNPs coupled to capture antibodies. With this architecture, the authors obtained a linear range between 0.5 and 25 ng mL^−1^ and a LoD of 0.17 pg mL^−1^. Still with a sandwich-type architecture, Feng et al. [67] described a CEA immunosensor based on signal amplification using Fc-functionalized Fe_3_O/SiO_2_ as labels and AuNP/GO as substrate. Anti-CEA were immobilized on both the substrate and the signaling NPs, the latter having catalytic properties towards H_2_O_2_ oxidation (Figure 12). DPV was used to reoxidize Fc. The linear range was found between 1 pg mL^−1^ and 80 ng mL^−1^, with a LoD of 0.2 pg mL^−1^.

Very recently (2017), Miao et al. [68] also reported a sandwich-type assay for CEA, based on IrNPs acting as electrochemical signal amplifier. Polydopamine-reduced graphene oxide (PDA-RGO) was employed to immobilize anti-CEA capture antibodies, and IrNPs for coupling on signaling antibodies. The large surface area of PDA-RGO and the electrocatalytic properties of IrNPs for H_2_O_2_ reduction allowed a working potential of −0.6 V (vs. SCE) without the use of HRP. The sensor presented a linear range from 0.5 pg mL^−1^ to 5 ng mL−1 and a LoD of 0.2 pg mL^−1^. Finally, still for its catalytic property towards H_2_O_2_, AgPtNPs-functionalized GR was reported by Dai et al. [69]. The detection range was from 0.5 ng mL^−1^ to 140 ng mL^−1^ with a LoD of 0.2 ng mL^−1^.

These works demonstrated that that novel metallic nanoparticles sch ag AgPt, AuPt or Ir NPs could efficiently replace an enzyme such as HRP for the catalytic oxidation of a redox probe through H_2_O_2_ reduction, which ensures a more robust sensor (HRP, as any other enzymes, is fragile and works properly only in given conditions such at mild temperature, mild pH, etc.). The other way to get rid of enzymes is to use classical diffusing redox probes but, without amplification procedure, sensitivities are generally lower. Figures of merit discussed in this section are summarized in Table 4.

• Immobilized Redox Probes

**Figure 13:**
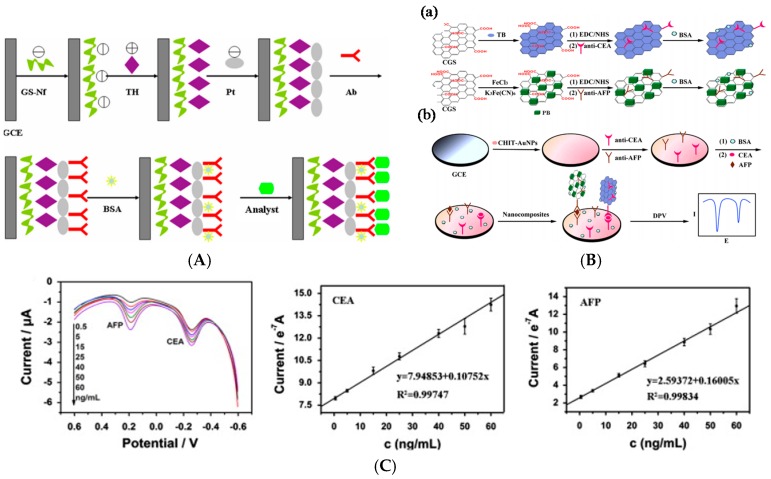
(**A**) Ab/PtNPs/Thi/Nafion/GR/GC electrode for detection of kanamycin. Reprinted from [71], Copyright 2012, with permission from Elsevier; (**B**) Antibody-modified and Prussian blue-modified GO (**a**) and multiplexed electrochemical immunoassay protocol for CEA and AFP detection (**b**); (**C**) *Left:* DPV responses of the immunosensor after incubation with CEA and AFP; *center* and *right:* corresponding calibration curves. Reprinted from [77], Copyright 2013, with permission from Elsevier.

To avoid some drawbacks generated by the use of diffusing redox probes, Among those who used immobilized redox probes, Mao et al. [70] described in 2012 an immunosensor for PSA using GR/MB/Chi as electrode material, onto which capture antibodies were immobilized. GR provided high specific area for increasing both the redox probe and the capture probe surface concentration. Using current changes of the CV peaks, induced by specific Ag-Ab interactions, the signal decreased linearly with PSA concentration (0.05–5.00 ng mL^−1^), with a LoD of 13 pg mL^−1^. Wei et al. [71] described a similar idea where the redox probe (here Thi) was immobilized on graphene/PtNPs, for detection of kanamycin, an antibiotic used to treat severe bacterial infections. The anti-kanamycin Ab was immobilized onto the modified electrode through electrostatic adsorption (Figure 13A). 

The authors claimed that Thi electroactivity was improved not only by graphene but also by PtNPs (due to an increase in surface area and conductivity). They reported a LoD of 6 pg mL^−1^ and a linear range from 0.01 to 12 ng mL^−1^. In 2013, Yu et al. [72] also described a kanamycin sensor with capture antibodies labelled with Ag/Fe_3_O_4_NPs and immobilized on a thionine/graphene-modified electrode. CV and SWV were used to characterize the recognition of kanamycin through change in Thi electroreduction currents, which decreased upon increase of kanamycin concentration. The LoD was 15 pg mL^−1^, and the linear range was from 0.050 and 16 ng mL^−1^. Cai et al. [73] described in 2012 a nanotubular mesoporous PdCu alloy for a label-free electrochemical immunosensor for CEA. It operated through physisorption of anti-CEA on NM-PdCu sulfonated graphene sheets modified with Thi as immobilized redox probe. From CV peak currents, a linear response was observed between 0.01 and 12 ng mL^−1^ with a LoD of 5 pg mL^−1^. In 2014, Wu et al. [74] also reported an immunoassay for CEA and squamous cell carcinoma antigen (SCCA) for the diagnosis of cervical cancer. Tetraethylene pentaamine-modified RGO was used to immobilize the primary antibody, and Au@mesoporous carbon CMK-3 were used to couple secondary antibodies (Ab_2_) with Neutral Red or Thi as redox probes. A conventional sandwich assay followed by DPV measurements showed that the immunosensor presented a linear range up to 20 ng mL^−1^ and a LoD of 10 pg mL^−1^. Han et al. [75] also reported a CEA sensor, but based on graphene decorated with AuNPs and Thi as redox probe; anti-CEA were immobilized on the AuNPs. The immunosensor showed a very low LoD of 0.05 fg mL^−1^ and a linear response from 0.1 fg mL^−1^ to 1 μg mL^−1^.

Lin et al. [76] described a AuNP-functionalized mesoporous carbon foam (MCF) coupled with carbon–Au synergetic silver enhancement for immunosensing of CEA. Antibodies were grafted on RGO/Chi-modified electrodes. Through a sandwich-type immunoreaction, Au/MCF tags were captured on the immunoconjugate to induce a silver deposition process. Using anodic stripping voltammetry (ASV) for silver redissolution, the authors found a linear range from 0.05 pg mL^−1^ to 1 ng mL^−1^ and a LoD of 0.024 pg mL^−1^. This technique of deposition/redissolution of metallic NPs is particularly efficient because it provides a very large amplification compared to other approaches. The other approaches which were published later did not feature such strategy and showed less satisfactory limits of detection.

Chen et al. [77] also reported CEA detection, but with a sandwich-format, using graphene sheets modified by toluidine blue for labeling anti-CEA, and Prussian Blue (PB) for labeling anti-AFP. The capture antibodies were immobilized onto chitosan-AuNPs immobilized on the electrode (Figure 13B). Using the electroactivity of the two different immobilized redox probes, a linear range of 0.5–60 ng mL^−1^ was obtained for both targets, and LoDs were of 0.1 ng mL^−1^ for CEA and 0.05 ng mL^−1^ for AFP. Wang et al. [78] described a CEA immunosensor using AuNPs-decorated mesoporous silica KIT-6. AuNPs were used to immobilize both the secondary antibodies and toluidine blue. For the immobilization of primary antibodies (Ab_1_), APTES-functionalized graphene sheets decorated with AuNPs were used as substrate on glassy carbon electrodes (GCE). Using DPV to sense toluidine blue electroactivity, this immunosensor exhibited a LoD of 3 fg mL^−1^ and a linear range from 10^−5^ ng mL^−1^ to 10^2^ ng mL^−1^. More recently, in 2016, Peng et al. [79] reported an electrochemical immunosensor for CEA, based on GR/Chi/Fc used to immobilize the primary antibodies. Fe_3_O_4_/AuNPs were also functionalized with antibodies, for signaling through a sandwich pathway. *p*-Aminophenol was used as redox probe, and Fc as catalyst of p-aminophenol electrooxidation. This immunosensor offered a linear signal between 1 pg mL^−1^ and 30 ng mL^−1^, with a LoD of 0.4 pg mL^−1^. Last example for CEA detection, Gao et al. [80] described a Nile blue–modified GO which was electrochemically reduce in the presence of AuCl_4_^−^, to led to a AuNPs/NB/RGO-modified electrode. CEA antibodies were immobilized on this nanocomposite. Upon formation of the Ab/Ag immunocomplex, Nile Blue electroactivity decreased proportionally to the CEA concentration. A linear response was obtained between 1 pg mL^−1^ and 40 ng mL^−1^, with a LoD of 0.5 pg mL^−1^.

No more for CEA but for AFP detection, Qi et al. [81] reported a PdNPs-functionalized RGO used as substrate for anti-AFP immobilization. H_2_O_2_ was used as redox probe added in solution. Upon formation of the Ab/Ag immunocomplex, the amperometric current of H_2_O_2_ reduction was proportional to the concentrations of AFP. The LoD was 5 pg mL^−1^ and the response linear from 0.01 to 12 ng mL^−1^. Very recently (2017), and for AFP detection, Wang et al. [82] used Cu_2_O-decorated GO as substrate for immobilization of AFP-antibodies. Toluidine Blue (TB) was used as redox probe, adsorbed on the GO. Detection was performed using SWV to monitor the electroactivity of TB upon immunoreaction. The linear range was between 1 fg mL^−1^ and 100 ng mL^−1^, with a LoD of 0.1 fg mL^−1^.

**Figure 14:**
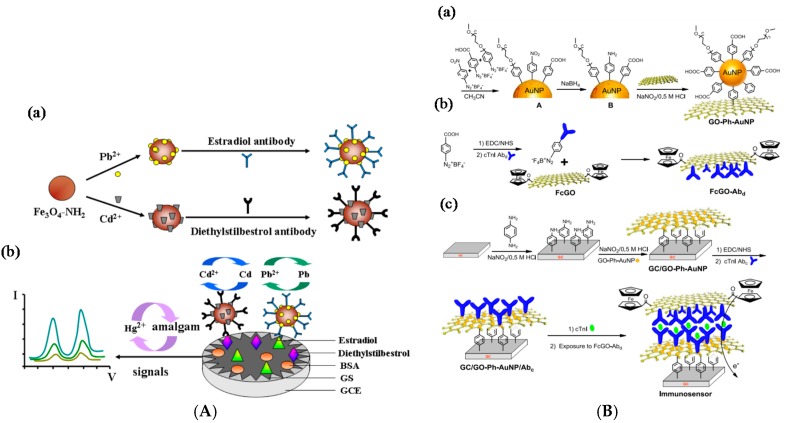
(**A**) (**a**) Fe_3_O_4_/M^2+^/Ab labels and (**b**) immunosensing and transduction for estradiol and diethylstilbestrol detection. Reprinted from [86], Copyright 2014, with permission from Elsevier. (**B**) (**a**) GO/AuNP; (**b**) Fc-modified and Ab-modified GO; (**c**) Assembly of these functionalized GO sheets for cTnI detection. Drawings are not at scale. Reprinted from [87], Copyright 2016, with permission from Elsevier.

Other antigens were also reported. For example, Wang et al. [83] described a sandwich electrochemical immunoassay for avian leukosis virus, using Fe_3_O_4_ NPs modified with graphene quantum dots (GRD), apoferritin-encapsulated CuNPs and signaling antibodies (Ab_2_). After a sandwich-type assembly with capture antibodies immobilized on the working electrode, CuNPs were released from the apoferritin cavity under anodic stripping voltammetry (ASV). Detection was achieved between 120 and 30,000 TCID_50_ mL^−1^ (50% Tissue Culture Infective Dose) with a detection limit of 115 TCID_50_ mL^−1^. The same year, Wu et al. [84] reported a dual signal amplification strategy for tumor cells detection. Graphene was used to increase the electrode area, covalently immobilize capture antibodies and accelerate electron transfer, and ZnSe or CdTe-coated SiO_2_NPs were used to label signaling antibodies. A classical sandwich-type immunoreaction was followed by dissolution of the tracing NPs into HNO_3_; this solution was then analyzed by SWV, which gave signals proportional to the quantity of capture tags, therefore proportional to the quantity of detected cell, from 10 to 10^6^ cells mL^−1^. Shi et al. [85] reported an electrochemical immunoassay for interleukin-6 (IL-6) and matrix metallopeptidase-9 (MMP-9) using polystyrene/polydopamine/silverNPs (PS/PDA/AgNPs) and graphene nanoribbon (GNR), in a sandwich assay format. Capture antibodies were immobilized on graphene and PS@PDA/AgNPs were used to label signaling antibodies. The detection range was reported between 10^−5^ and 10^3^ ng mL^−1^ and the LoD was 5 fg mL^−1^ for IL-6 and 0.1 pg mL^−1^ for MMP-9. Zhang et al. [86] described an immunosensor for simultaneous detection of estradiol and diethylstilbestrol. Amino-functionalized mesoporous Fe_3_O_4_ NPs was loaded with Pb^2+^ or Cd^2+^, and then incubated with anti-estradiol and anti-diethylstilbestrol antibodies, respectively. Estradiol and diethylstilbestrol antigens were adsorbed on graphene sheets deposited on GC electrodes. Using SWASV, two well-separated peaks were generated by the redox reaction of Pb^2+^ or Cd^2+^ on the electrode, making the simultaneous detection of the two antigens possible (Figure 14A). Peak currents were proportional to the concentrations of estradiol and diethylstilbestrol in the range from 0.050 pg mL^−1^ to 100 ng mL^−1^ and 1.0 pg mL^−1^ to 100 ng mL^−1^, respectively. The detection limits were of 0.015 pg mL^−1^ and 0.38 pg mL^−1^, respectively.

Again, one can see form these works that strategies using the electroactivity of metallic NPs generally provide lower detection limits than more classical approaches.

**Figure 15:**
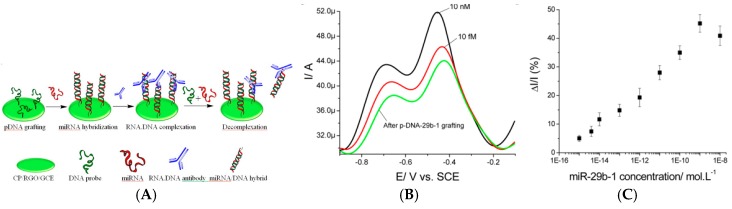
(**A**) Description of miRNA immunosensing; (**B**) SWVs after pDNA-29b-1 probe grafting, after hybridization with 10 fM miR-29b-1 and with 10 nM miR-29b-1; (**C**) Calibration curve: Δ*I*/*I* (%) vs. miR-29b-1 concentration. Medium: PBS. Relative current changes were calculated as follows: *I* = *Ibefore hybridization* and Δ*I* = *I*_after hybridization_ − *I*_before hybridization_. Adapted with permission from [89]. Copyright 2013 American Chemical Society.

Liu et al. [87] reported an amperometric immunosensor based on AuNPs and GO for detection of cardiac troponin-I. GO and AuNPs were anchored on the electrode surface by covalent bonding using aryldiazonium coupling. Antibodies were immobilized on GO along with Fc as signal reporter (Figure 14B). Using SWV, they demonstrated a LoD of 0.05 ng mL^−1^. Tuteja et al. [88] described a label-free immunosensor based on EIS for detection of myoglobin. Graphene quantum dots (GQDs) have been used as an immobilized template on SPE. GQDs were conjugated with anti-myoglobin antibodies and charge transfer resistance was used as sensing parameter, with a linear variation between 0.01 and 100 ng mL^−1^ and a LoD of 0.01 ng mL^−1^. At last, Tran et al. [89] reported an original label-free immunosensor for detection of microRNAs (miRNA) based on a conducting polymer/reduced graphene oxide-modified electrode. SWV was used to record the redox signal of the conducting polymer. Current increases upon hybridization (signal on) from 1 fM to 1 nM of target miRNA. The limit of quantification was 5 fM. To double-check its selectivity, two specific RNA–DNA antibodies recognizing miRNA–DNA heteroduplexes were used, anti-poly(A)–poly(dT) and anti-S9.6 (Figure 15). Complexation of the antibody with the hybrid led to a current decrease that confirmed the presence of miRNA, down to a concentration of 8 fM. Last, when the RNA/DNA hybrid was added in solution, the authors observed a specific re-increase of the SWV current, attributed to the competitive decomplexation of the antibodies. This last example shows, as it was the case for [75], the superiority of immobilized redox probes over diffusing ones in terms of limit of detection. Figures of merit discussed in this section are summarized in Table 5.

#### 3.3.2. Enzyme-Based Immunosensors

The previously cited immunosensors were designed to transduce Ab-Ag complexation without amplification, or with a non-enzymatic catalytic amplification process. However, despite their limitations in terms of stability, enzyme-based immunosensors are also widely reported.

There are again few examples of immunosensors for antibody detection, and most works report human IgG detection not for practical application but as a model analyte. Liu et al. [90] reported in 2012 an IgG sensor based on layer-by-layer assembly of MWCTs and RGO, using poly(diallyldimethylammonium) (PDDA) as binding polymer between layers. In a sandwich-type immunoassay, human IgG was uFsed as the target antigen, HRP-conjugated IgG as the probing antibody and hydroquinone (HQ) as the electron mediator. The LoD was 0.2 ng mL^−1^ and the linearity was observed from 1 ng mL^−1^ to 500 ng mL^−1^. In 2014, Lai et al. [91] also described an immunoassay for IgG as a model analyte. The working electrode was modified with RGO/AuNPs and capture antibodies, whereas signaling antibodies were coupled to AuNPs-labeled HRP. 

**Figure 16:**
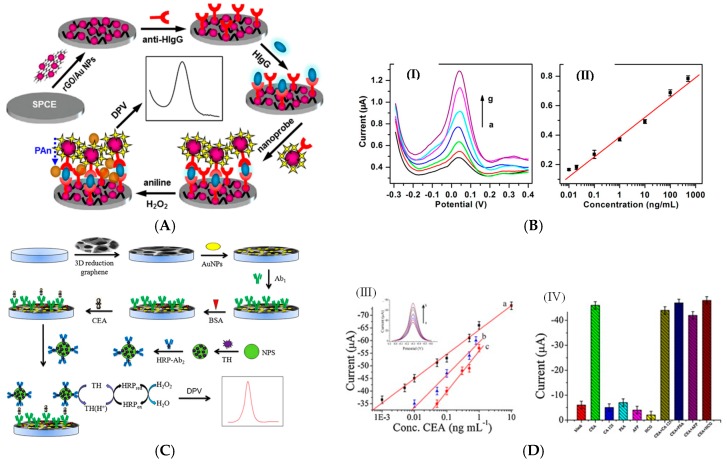
(**A**) Sandwich immunoassay for IgG detection using HRP-AuNP for oxidation of aniline into polyaniline; PANi is detected by DPV. Adapted with permission from [91]. Copyright 2014 American Chemical Society; (**B**) DPV responses of the immunosensor toward different concentrations of IgG (I) and (II) corresponding calibration curve. Curves a–g correspond to IgG concentrations from 0.01 to 500 ng mL^−1^ (**C**) Fabrication process of HRP-Ab_2_/Thi/AuNPs and electrochemical transduction; (**D**) (III) Calibration curves for CEA in pH 7.0 PBS + 2 mmol L^−1^ H_2_O_2_ for different electrode materials: (a) 3D-AuNPs/GR (inset: DPV curves), (b) GR, (c) AuNPs. (IV) Interferents and associated currents. Reprinted from [93], Copyright 2013, with permission from Elsevier.

After performing a sandwich immunoreaction, HRP catalyzed the oxidation of aniline (added in solution along with H_2_O_2_) to produce electroactive polyaniline on the electrode surface (Figure 16A). Polyaniline (PANi) electroactivity was probed by DPV. The LoD was 10 pg mL^−1^ and the linear range was reported from 100 pg mL^−1^ up to 1 μg mL^−1^.

There are much more examples of enzymatic immunosensors for antigen detection. In 2012, Yan et al. [92] described a PSA sandwich-type immunosensor using graphene nanosheets and HRP-labeled signaling antibody coupled to AuNPs. AuNPs provided a large surface area for the immobilization of HRP-Ab but the authors claimed that they are used also for their participation in the electroreduction of H_2_O_2_. HQ was used as redox mediator, and DPV as the electrochemical technique. This immunosensor showed a linear range between 2 pg mL^−1^ (the LoD) and a few μg mL^−1^. Sun et al. [93] described in 2013 an electrochemical immunoassay for CEA based on 3D AuNPs/GR as substrate to immobilize anti-CEA capture antibodies, and nanoporous AgNPs onto which Thi, HRP and signaling antibodies were immobilized (Figure 16C). Using DPV, the authors found a detection range from 1 pg mL^−1^ to 10 ng mL^−1^ and a LoD of 0.35 pg mL^−1^. Liu et al. [94] also reported a CEA immunosensor, based on a GR macroporous foam as substrate for non-covalent immobilization of the capture antibody. A lectin (concanavalin A) monolayer was immobilized on the GR electrode using polydopamine as linker. HRP-labeled capture anti-CEA were immobilized on the lectin through the specific sugar-protein interaction (specific affinity between concanavalin A and sugar chains located on the surface of HRP). With Fe(CN)_6_^3−/4−^ as redox probe and DPV for measurements, CEA was detected within a linear range of 0.1–750 ng mL^−1^ (LoD of ca. 90 pg mL^−1^). This approach must attract our attention because, conversely to conventional grafting of antibodies on a solid substrate, which cannot be well controlled in terms of Ab orientation on the surface, this immobilization by affinity insures that all probe antibodies are well oriented on the surface and are available for further target (antigen) recognition.

Still for CEA detection, Yang et al. [95] described a streptavidin-functionalized nitrogen-doped graphene where biotinylated antibodies were immobilized. HRP-labelled antibodies were used with a sandwich immunoassay, with Thi and H_2_O_2_ as redox probes. With DPV, the authors demonstrated a linear detection range from 0.02 to 12 ng mL^−1^ and a LoD of 0.01 ng mL^−1^.

Graphene-based immunosensors were also designed for detection of hormones. For example, Li et al. [96] described in 2013 a 17β-estradiol immunosensor based on a GR/PANi composite as electrode substrate, and HRP-labelled signaling antibodies coupled to GO sheets. They used a competitive immunoassay with HQ as redox mediator. With DPV, they obtained a linear response to estradiol in the range 0.04–7.00 ng mL^−1^ and a LoD of 0.02 ng mL^−1^. Cincotto et al. [97] also reported ethynylestradiol (EE2) detection using a AgNPs/SiO_2_/GO composite grafted on a GC electrode through the diazonium route, onto which anti-EE2 was also covalently bound. HRP-EE2 was used in a competitive assay, with HQ as substrate. The current was monitored at a constant potential of −200 mV and led to a LoD of 65 pg mL^−1^ and a linear range between 0.1 and 50 ng mL^−1^. Sun et al. [98] reported very recently (2017) detection of cortisol using AuNPs/GO. Cortisol, was first coupled to AuNPs/GO immobilized on a GCE by a Nafion membrane. Upon addition of HRP-labeled anti-cortisol antibodies, a competition occurred, which was quantified by measuring the HRP activity by DPV (o-phenylenediamine and H_2_O_2_ as substrate and co-substrate, respectively, diffusing in solution). This immunosensor displayed a detection range from 0.1 to 1000 ng mL^−1^ and a LoD of 0.05 ng mL^−1^.

**Figure 17:**
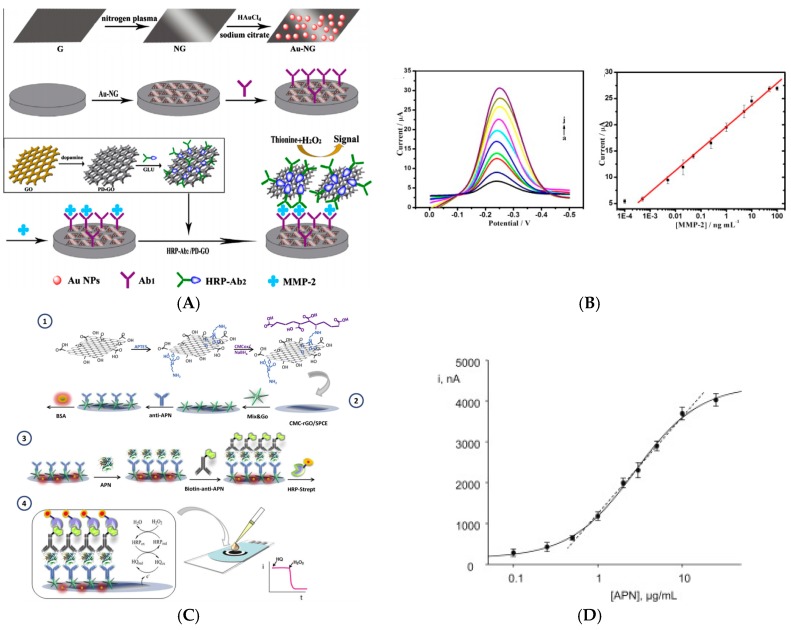
(**A**) Preparation and detection procedures for the MMP-2 immunosensor. Reprinted from [99], Copyright 2013, with permission from Elsevier; (**B**) Typical DPVs corresponding to MMP-2 concentrations from a to j (0, 0.0005, 0.005, 0.02, 0.05, 0.25, 1.0, 5.0, 10.0 and 50.0 ng mL^−1^) in PBS + 4 mM H_2_O_2_, and corresponding calibration curve; (**C**) The different steps involved in the construction of the adiponectin (APN) immunosensor using a metallocomplex polymer (CMC) and RGO on a screen-printed carbon electrode; (**D**) Corresponding calibration plot. Reprinted from [101], Copyright 2016, with permission from Elsevier.

Detection of protein biomarkers was also described. Yang et al. [99] reported detection of matrix metalloproteinase-2 (MMP-2) by immobilizing AuNPs on N-doped graphene sheets, onto which capture antibodies were immobilized. Signaling antibodies were coupled to GO sheets decorated by HRP-labelled signaling antibodies (Figure 17A). Upon addition of Thi and H_2_O_2_ in solution, DPV was used to measure the HRP activity through Thi reduction. They found a linear range from 0.5 pg mL^−1^ to 50 ng mL^−1^, with a LoD of 0.1 pg mL^−1^.

These works show that two approaches could be followed to improve the sensors signal. The first approach consists in using a surface area which is increased by using a nanostructured substrate where probes are immobilized at a high surface density, then a classical labelling of secondary antibodies brings amplification. A second approach, different, tries to increase the surface concentration of the labels by immobilizing them on NPs or other nanostructures such as nanosheet; it is not the surface density of probes which is increased, but that of the redox label.

In 2015, Fang et al. [100] reported the detection of HIV-p24 protein (the protein envelope of HIV) using MWCNTs-silica as a matrix for HRP and capture antibodies, and GO as carrier of Thi and HRP-modified signaling antibodies. In a sandwich immunoreaction, HRP/MWCNTs/SiO_2_ and HRP/Thi/GO captured onto the electrode surface produced an amplified electrocatalytic response by electroreduction of the Thi_Ox_ produced by HRP. The increase of current was proportional to the HIV-p24 concentration between 0.5 pg mL^−1^ and 8.5 ng mL^−1^, with a LoD of 0.15 pg mL^−1^. At last, Arenas, Sánchez-Tirado et al. [101] presented the detection of adiponectin (APN), an hormone involved in glucose and lipid metabolism with a central role in the regulation of insulinoresistance, with a sandwich-type assay involving a metal complexes-based polymer (Mix & Go™) for immobilization of the anti-APN capture antibody, and HRP-anti-APN as signaling antibody (Figure 17C). Using amperometry at −200 mV and the H_2_O_2_/HQ redox system in solution, they obtained a linear calibration within 0.5–10.0 μg mL^−1^; the LoD was 60 ng mL^−1^.

There is one example of carbohydrate immunosensor. Yang et al. [102] reported a sandwich-type sensor for carbohydrate antigen (CA19-9), using AuNPs-functionalized GO as substrate where capture antibodies were immobilized, and Au@PdNPs-functionalized GR as signal enhancer, bearing thionine (Thi) and HRP-labelled signaling antibodies. Upon addition of H_2_O_2_ and using DPV for Thi_ox_ reduction, the electrochemical immunosensor exhibited linearity between 0.015 and 150 U mL^−1^ and a LoD of 6 mU mL^−1^.

**Figure 18:**
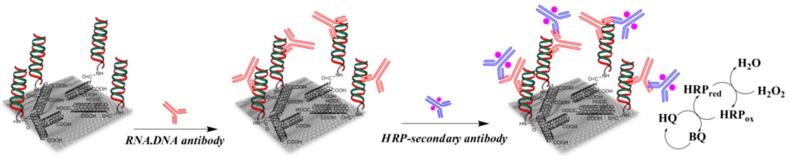
Electrochemical ELISA-like immunosensor for micro-RNA detection. Reprinted from [103], Copyright 2014, with permission from Elsevier.

At last, there is one example of miRNA immunodetection. Piro et al. [103] reported a composite of RGO and MWCNTs as substrate for immobilization of single-stranded oligonucleotide probes. Upon hybridization of the target miRNA on this probe, HRP-labelled anti-DNA/RNA hybrids were added. Upon addition of HQ and H_2_O_2_ in solution, HQ was oxidized into BQ, which was then re-reduced into HQ at the electrode (Figure 18). Using SWV, the authors reported a LoD of 10 fM miRNA and a linear range between 10 fM and 0.1 nM. Figures of merit discussed in this section are summarized in Table 6.

### 3.4. Metal or Metal Oxide Nanoparticles

In the previous section dealing with graphene-based devices, metal nanoparticles (e.g., AuNPs) were already implemented in addition to graphene. In the following section are more generally reviewed electrochemical immunosensors featuring metal or metal-oxide NPs, used to increase the surface area of the sensing layer but also and mainly to provide a catalytic activity in order to replace an enzymatically-catalyzed reaction (in enzyme-less immunosensors) or even to catalyze enzymatic products (in enzyme-based immunosensors).

#### 3.4.1. Enzyme-Based Immunosensors

There is only one example of antibody sensor, published in 2013. Gao et al. [104] described a signal-amplified sensor for IgG using TiO_2_ nanotube array, for their large surface area, high pore volume and good electrochemical conductivity. AuNPs were functionalized with HRP-tagged antibodies and immobilized on the nanotubes. This architecture allowed for a higher surface concentration of HRP-labelled Ab compared to flat electrodes, which led to higher amplified electrochemical signals from the catalytic reaction of HRP relative to hydrogen peroxide (H_2_O_2_) and its electroreduction of the AuNPs. It exhibited a detection range from 0.1 to 10^5^ ng mL^−1^ with a LoD of 0.01 ng mL^−1^.

**Figure 19:**
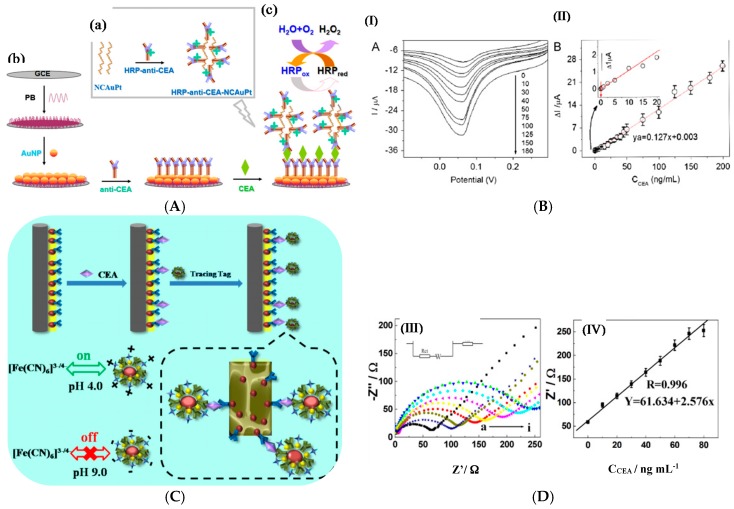
(**A**) (**a**) Preparation of the HRP-anti-CEA-AuPt nanochains as label; (**b**) sandwich-type recognition; (**c**) transduction. (**B**) Corresponding LSV (I) and calibration (II) curves for CEA at different concentrations in PBS + 0.8 mM H_2_O_2_. Reprinted from [105], Copyright 2013, with permission from Elsevier. (**C**) Detection of CEA with AuNPs-Ab_2_-GOx as label. (**D**) EIS (III) for CEA at different concentrations: (a) 5, (b) 10, (c) 20, (d) 30, (e) 40, (f) 50, (g) 60, (h) 70 and (i) 80 ng mL^−1^ in 0.1 M KCl + 5.0 mM Fe(CN)_6_^3−/4−^; (IV) corresponding calibration curve. Reprinted from [106], Copyright 2016, with permission from Elsevier.

All other works described immunosensors for antigens, mostly proteins. In 2013, Cao et al. [105] described bimetallic AuPt nanochains (NC) onto which HRP-conjugated anti-CEA signaling antibodies were attached, giving “hairy” enzyme-labeled nanoparticles (HRP-anti-CEA-NCAuPt). Detection of CEA was performed in a sandwich-type immunoassay format, with H_2_O_2_ as enzyme substrate and Prussian Blue (PB) as mediator, both added in solution. PB was immobilized under a AuNPs layer on the electrode (Figure 19A). The linear detection range was 0.01–200 ng mL^−1^ and the LoD 0.11 pg mL^−1^.

**Figure 20:**
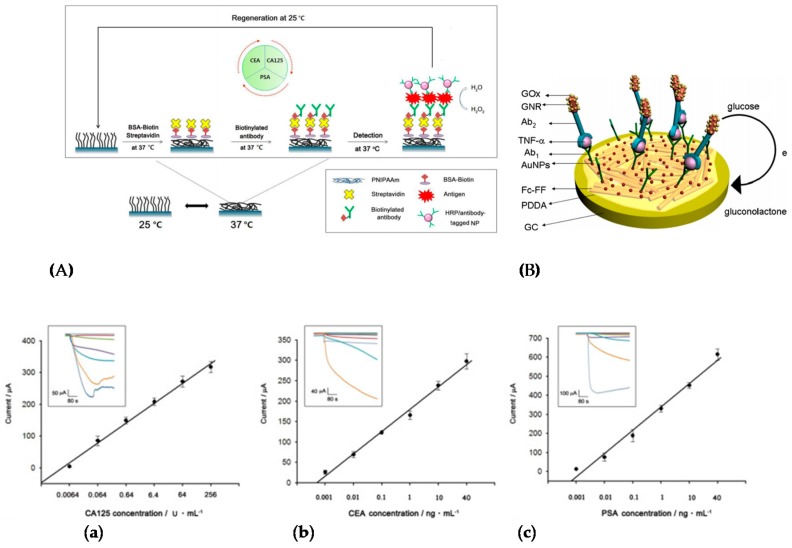
(**A**) PNIPAAm-based immunosensor for CA125, CEA and PSA detection; (**a**–**c**) Electrochemical response of this sensor to CA125, CEA, and PSA, respectively. Chronoamperometric results were obtained at −0.2 V. After incubation with the target proteins, HRP/antibody-tagged PPy NPs were bound to the primary antibody on the PNIPAAm-Au, and 0.1 M hydroquinone and 0.5 mM H_2_O_2_ were addede in PBS buffer. Reprinted from [107], Copyright 2016, with permission from Elsevier; (**B**) GOx-modified gold nanorods for TNF-α detection. Reprinted from [111], Copyright 2013, with permission from Elsevier.

Song et al. [106] described more recently a CEA immunosensor, based on an original transduction scheme based on a sandwich architecture. Signaling antibodies were modified with AuNPs, GOx and concanavalin A and macroporous carbon modified with capture antibodies as signal collector (substrate) (Figure 19C). Concanavalin A was used because its surface charge varies significantly with pH. Therefore, when Fe(CN)_6_^3−/4−^ was used as redox mediator in solution, the GOx activity, generating local acidification, changed the neat charge of the NPs and repulsed the redox probe from the NPs surface, therefore lowered the current. Using EIS, the charge transfer resistance changed proportionally toward CEA concentration from 4 pg mL^−1^ to 50 ng mL^−1^ with a LoD of 1.3 pg mL^−1^. Hong et al. [107] presented also in 2016 an electrochemical immunosensor for detection of three tumor markers: CA125, CEA, and PSA. They used as substrate a temperature-responsive polymer (poly(N-isopropylacrylamide—PNIPAAm) for temperature-induced regeneration of the electrode/electrolyte interface (Figure 20A). HRP-labeled antibodies were coupled to polypyrrole NPs for signaling. A linear range from the pg mL^−1^ to 100 ng mL^−1^ was reported, with LoDs in the pg mL^−1^ range depending on the target. Li et al. [108] also reported a sensor for PSA. The paper-based device was fabricated by sequentially growing AuNPs on cellulose fibers, then MnO_2_ nanowires, to form a 3D network with a large surface area. GOx was used as enzyme label and 3,3′,5,5′-tetramethylbenzidine (TMB) as a redox mediator. The linear detection range, obtained by amperometry at −0.2 V, was between 5 pg mL^−1^ and 100 ng mL^−1^ with a LoD of 1 pg mL^−1^.

Detection of AFP was also reported. For example, Zhang et al. [109] reported a microfluidic bead-based immunosensor that used multienzyme-nanoparticle amplification and quantum dots labels. Microbeads were functionalized with capture antibodies and AuNPs were functionalized with HRP-labelled signaling antibodies. Upon a sandwich immunoreaction, the activity of HRP was monitored and used as quantification signal. This immunosensor presented a LoD of 0.2 pg mL^−1^ AFP and showed a 500-fold increase in detection limit compared to the off-chip test, which demonstrated the pertinence to use flow cells instead of conventional ones. More recently (2016), Huo et al. [110] also reported a device for AFP detection, using TiO_2_ nanotubes functionalized by HRP, PANi and AuNPs. Protein G was crosslinked on the AuNPs for oriented immobilization of the capture anti-PSA antibody. Upon a sandwich-type immunoreaction, the HRP-labelled signaling antibody was captured on the surface and using DPV with HQ and H_2_O_2_ as a redox probe in solution, the authors obtained a linear range between 0.01 and 350 ng mL^−1^ and a LoD of 1.5 pg mL^−1^. This work, along with other works dealing with concanavalin A, underlines the great importance to take care of the orientation of the probe Ag for reaching the lowest limits of detection.

Applied to another kind of protein, Sun et al. [111] described detection of TNF-α using peptide-based self-assembled nanomaterials and GOx-modified gold nanorods (GNR). Anti-TNF-α antibodies were immobilized on Fc-peptide nanowires on the electrode surface, the Fc moiety acting as redox mediator for GOx. Secondary anti-TNF-α antibodies were labelled with GNR and GOx in a sandwich-type assay (Figure 20B). The SWV response towards glucose oxidation was used as signal to quantify TNF-α, with a wide linear range from 0.005 to 10 ng mL^−1^.

Still using metal or metal-oxide NPs for their catalytic properties, Zhang et al. [112] reported an electrochemical immunosensor for interferon-γ detection based on PDDA and AuNPs to provide a high-area interface, and HRP-labeled antibody-conjugated AuNPs (HRP-Ab_2_-AuNP) as signal tag. The oxidation of HQ by H_2_O_2_ was monitored by re-reducion of BQ into HQ at the electrode. The linear range was observed from 0.1 to 10,000 pg mL^−1^, with a LoD of 0.05 pg mL^−1^.

**Figure 21:**
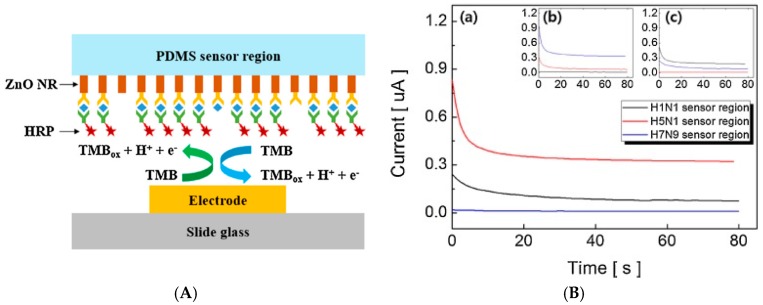
(**A**) Schematic representation of the structure of the ZnO nanorods microfluidic immunosensor for influenza virus detection; (**B**) Chronoamperometric curves measured from each electrode of the arrayed sensor, for an antigen mixture of (**a**) 1 pg mL^−1^ H1N1 and 100 pg mL^−1^ H5N1; (**b**) 10 pg mL^−1^ H5N1 and 100 pg mL^−1^ H7N9; and (**c**) 10 pg mL^−1^ H1N1 and 1 pg mL^−1^ H7N9, respectively. Reprinted from [113], Copyright 2016, with permission from Elsevier.

Metal NPs can also simply be used for their high surface-to-volume ratio. For example, Han et al. [113] described ZnO nanorods grown on PDMS for sensing H1N1, H5N1, and H7N9 influenza retrovirus simultaneously, using an original array architecture (Figure 21). They have shown that ZnO nanorods, in addition to their high specific area, have an isoelectric point of ca. 9.5 which allowed to interact electrostatically with capture antibodies which have a lower isoelectric point. The originality is that the substrate used for the immunoreaction is not directly the electrode but the PDMS wall of the microfluidic cell, in front of a working non-modified gold electrode. Using HRP-labelled signaling antibodies and TMB as substrate, they achieved a LoD of 1 pg mL^−1^ for each virus, with a linear detection range (measured through current-time curves) of 1–10 ng mL^−1^.

Very recently, Bravo et al. [114] reported polyvinyl alcohol-functionalized AgNPs (AgNPs-PVA) used in a microfluidic immunosensor for quantification of epithelial cell adhesion molecule (EpCAM), with the use of HRP-conjugated anti-EpCAM. With 4-tertbutylcatechol and H_2_O_2_ as substrate and cosubstrate added in solution, they obtained a LoD of 0.8 pg L^−1^.

At last, another way to use metals in electrochemical immunosensors is to generated production of M^0^ at the electrode vicinity, afterwards electrochemically dissolved using ASV or SWASV, as it has been reviewed in the first section of this article. Alves et al. [115] described AuNPs-modified carbon SPE for Ara-h1 (a peanut allergen) detection, based on AlkP-labelled signaling antibodies, able to produce Ag^0^ form Ag^+^ added in solution. Capture antibodies were grafted on AuNPs immobilized on the working electrode. The produced Ag^0^ was detected by ASV around 0.2 V. Ara-h1 was quantified between 13 and 2000 ng mL^−1^, with a LoD of 4 ng mL^−1^, which could be considered as a relatively high detection limit considering other reported works featuring ASV. Figures of merit discussed in this section are summarized in Table 7.

#### 3.4.2. Enzyme-Free Immunosensors

There are even more examples of enzyme-less immunosensors using metal of metal-oxide nanoparticles except for antibody detection, for which only one recent work has been reported, where Tabrizi et al. [116] IgG detection using AuNP-modified polypyrrole (PPy). Simply using Fe(CN)_6_^3−/4−^ as redox probe, IgG was detected, by measuring changes in charge transfer resistance with EIS, between 0.5 and 125 ng mL^−1^ with a LoD of 0.02 ng mL^−1^.

**Figure 22:**
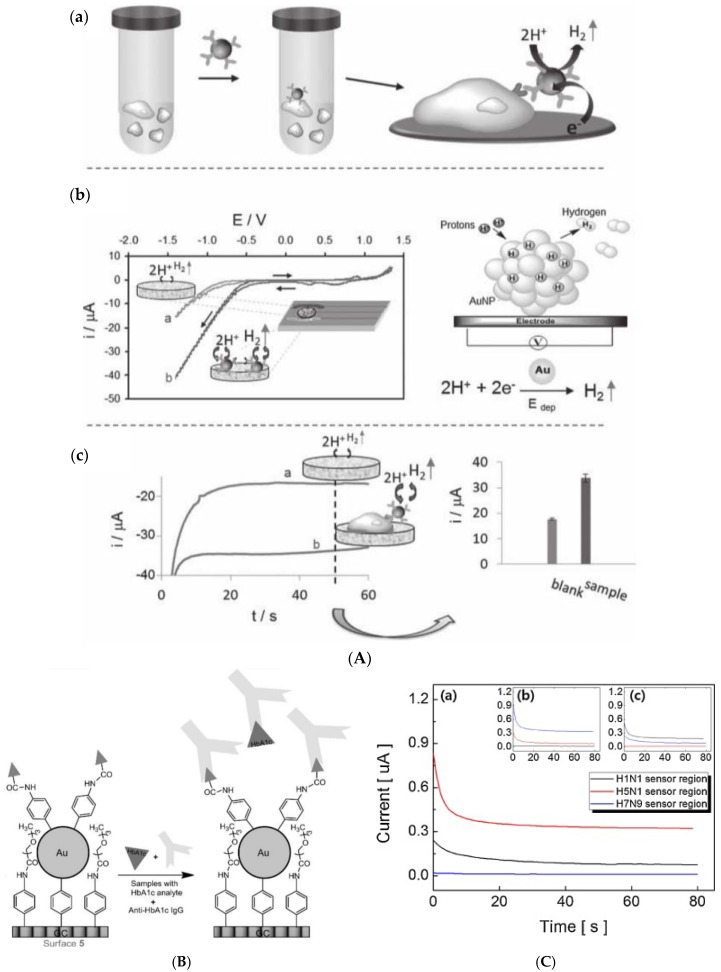

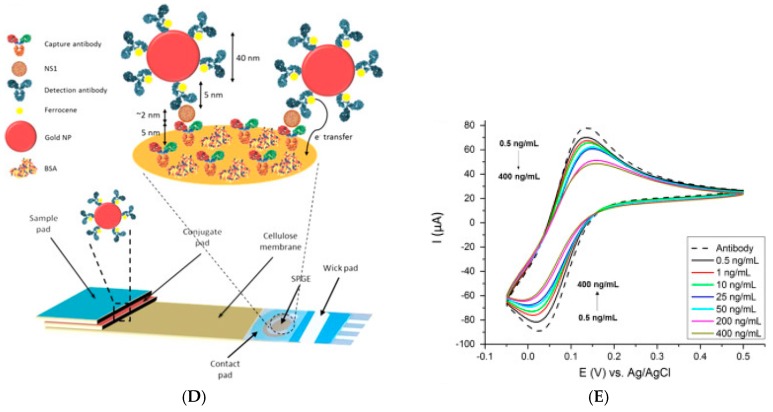
(**A**) (**a**) Scheme of the cells biorecognition with AuNPs-conjugated antibodies and detection through the electrocatalyzed HER; (**b**) CVs performed from +1.35 to −1.40 V at a scan rate of 50 mV/s in 1 M HCl in the absence (a curve) and in the presence (b curve) of AuNPs-conjugated antibodies; (**c**) *Left:* Chronoamperograms registered in 1 M HCl, during the HER applying a constant voltage of −1.0 V, for AuNPs-labeled cells (a: control with BSA; b: 3.5 × 10^4^ cells). *Right:* Comparison of the corresponding analytical signals for the blank and the positive sample. Reprinted from [117], Copyright^©^ 2012 WILEY-VCH Verlag GmbH & Co; (**B**) EIS immunosensor based on AuNPs for detection of HbA1c; (**C**) Impedance change with the concentration of target anti-HbA1c IgG (triangle dot), anti-biotin antibody (square dot), and anti-pig IgG (circle dot). Adapted from [118] with permissions. Copyright^©^ 2012 WILEY-VCH Verlag GmbH & Co; (**D**) Scheme of the electrochemical lateral flow immunosensor for dengue detection, with AuNPs/Fc-labeled antibodies; (**E**) CVs of NS1 protein detection over the lateral flow immunosensor, for concentrations from 0.5 ng mL^−1^ to 400 ng mL^−1^ of NS1 protein. Reprinted from [119], Copyright 2016, with permission from Elsevier.

For cell detection, Maltez-da Costa et al. described in 2012 a quantification assay based on a transduction by electrocatalysis of hydrogen evolution (HER) on AuNPs. The system, where cells are labeled by AuNPs through a specific immunoreaction, is described on Figure 22A; it can detect 4 × 10^3^ cancer cells in suspension [117]. It must be underlined that this amplification approach does need a reactant to be added in solution, conversely to most of the other amplified methodologies. Indeed, the HER reaction only used H_2_O, which is of course freely available in biological media such as those used for biosensing.

For antigen detection, Liu et al. [118] described in 2012 a label-free immunosensor for detection of HbA1c, based on AuNP-modified glycosylated pentapeptides (GPP) being analogs to HbA1c. Exposure of this interface to anti-HbA1c IgG resulted in a change in charge transfer resistance. Transduction was achieved using EIS and Ru(NH_3_)_6_^2+/3+^ as redox probe in solution, in a competitive inhibition assay where the surface bound GPP and HbA1c in solution competed for the anti-HbA1c IgG antibodies (Figure 22B). The LoD was of a few tens of ng mL^−1^.

More recently, Sinawang et al. [119] described an electrochemical lateral flow immunosensor for detection of dengue NS1 protein. A conventional lateral flow architecture was adapted to a sandwich-type electrochemical transduction provided by a AuNP-labeled NS1 antibody, with Fc grafted on the AuNPs. From EIS measurements (and more particularly the total resistance of the electrode), a linear calibration range was shown between 1 and 25 ng mL^−1^, with a LoD of 0.5 ng mL^−1^ (Figure 22D).

**Figure 23:**
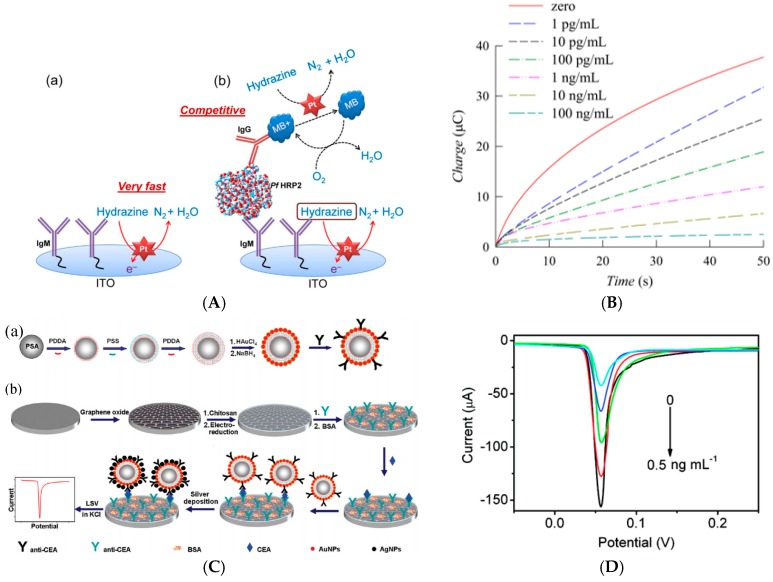
(**A**) *Pf*HRP2 immunosensor in the absence (**a**) and presence (**b**) of the target antigen with the MB-labelled detection antibody; (**B**) Chronocoulograms of PBS spiked with *Pf*HRP2 recorded at 0.05 V (vs. Ag/AgCl). Reprinted from [122], Copyright 2016, with permission from Elsevier; (**C**) Scheme of (**a**) construction of signaling AuNPs by layer-by-layer alternate assembly of PDDA and PSS, and (**b**) sandwich immunoassay with Ag deposition and AgNPs redissolution, for CEA detection, on a chitosan/RGO-modified GC electrode; (**D**) Linear sweep stripping voltammetric curves of AgNPs for detection of CEA from 0 to 0.5 ng mL^−1^. Reprinted with permission from [127]. Copyright 2012 American Chemical Society.

Lu et al. [120] reported an electrochemical immunosensor for detection of two mycotoxins, fumonisin B1 and deoxynivalenol. A SPE was modified by AuNPs, PPy and RGO. Capture antibodies were immobilized on the AuNPs and free-diffusing Fe(CN)_6_^3−/4−^ was used as redox probe. The LoD was of a few ng L^−1^ for both targets and the detection range between 50 ng L^−1^ and 1 μg L^−1^. Very recently, Carneiro et al. [121] described an immunosensor for detection of amyloid beta 1–42 protein Aβ(1–42). A gold electrode was modified with a monolayer of mercaptopropionic acid, electrodeposited AuNPs and thiolated capture antibodies. Using Fe(CN)_6_^3−/4−^ as redox diffusing probe, Aβ(1–42) was detected within a linear range of 10 to 1000 pg mL^−1^ and a LoD of 5 pg mL^−1^. Also very recently, Dutta et al. [122] reported an enzyme-free electrochemical immunosensor based on a competitive detection scheme using MB, hydrazine and PtNPs in an immunosandwich format (Figure 23A). In the presence of the target antigen, surface-immobilized MB consumes interfacial hydrazine thereby diminishing the electro-oxidation of hydrazine on PtNPs. This sensor was used to detect *Plasmodium falciparum* histidine-rich protein 2 (*Pf*HRP2). Chronocoulometric measurements allowed a LoD in the pM range (ca. 70 ng L^−1^).

In the same spirit, Viswanathan et al. [123] proposed a multiplexed detection of *Escherichia coli*, campylobacter and salmonella. A mixture of anti-*E. coli*, anti-campylobacter and anti-salmonella antibodies were immobilized on a MWCNT-polyallylamine-modified carbon SPE, then incubated in a bacteria suspension. The sandwich immunoassay was performed with CdS, PbS or CuS-conjugated signaling antibodies, used to release the corresponding metal ions upon SWASV. Calibration curves were obtained in the range 10^3^–5.10^5^ cells mL^−1^, with a LoD of 400 cells mL^−1^. Direct detection of carbohydrate antigens (CA72-4) was also reported by Fan et al. [124] using nanoporous gold as substrate and polyaniline-AuNPs (PANi-AuPt) as label. The signaling antibody (Ab_2_) was adsorbed onto the PANi-AuNPs and served as catalyst for H_2_O_2_ oxidation (added in solution). The sensor exhibited a linear range from 2 to 200 U mL^−1^, with a LoD of 0.10 U mL^−1^.

At last, detection of small organic molecules was also reported. Liu et al. [125] described a label-free electrochemical immunosensor for atrazine (ATZ) detection using AuNPs-labeled antibodies. Fe(CN)_6_^3−/4−^ was used as electrochemical redox indicator in solution. Binding of anti-ATZ and ATZ was monitored with DPV. A LoD of 0.02 ng mL^−1^ was obtained, with a linear range between 0.05 ng mL^−1^ and 0.5 ng mL^−1^. Similarly, Vabbina et al. [126] reported cortisol detection based on immobilized antibodies on ZnO nanorods or ZnO nanoflakes. Fe(CN)_6_^3−/4−^ was added in solution for probing the presence of the Ab/Ag complex. The use of ZnO nanoparticles were justified not for their catalytic properties but for an increase in surface area. Using CV and EIS, the found a LoD of 1 pM (ca. 0.4 ng L^−1^).

**Figure 24:**
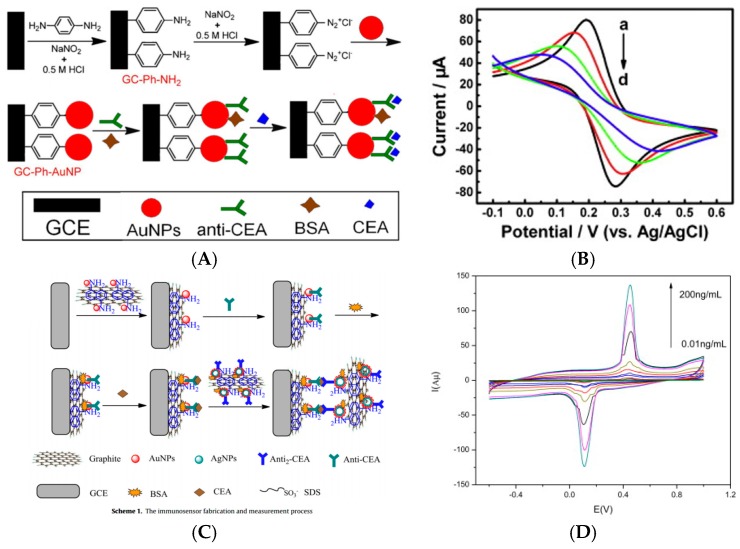
(**A**) GC/Ph/AuNP competitive immunosensor for CEA detection; (**B**) CVs of the modified electrode in 10 mM [Fe(CN)_6_]^4−/3−^ + 0.1 M KCl, for (a) a bare electrode; (b) NH_2_-Ph-NH_2_-modified electrode; (c) NH_2_-Ph-NH_2_ + AuNPs and (d) NH_2_-Ph-NH_2_ + AuNPs + Anti-CEA. Reprinted from [130]. Copyright 2012, with permission from Elsevier; (**C**) Ag/AuNPs coated on GR as CEA immunosensor; (**D**) CVs obtained in PBS form a Ag/AuNPs/GR electrode, showing the oxidation/reduction of the Ag^0^/Ag_2_O couple for different concentrations of CEA. Reprinted from [139], Copyright 2015, with permission from Elsevier.

Detection of more classical antigens were also reported with metal or metal oxide-based immunosensors, such as CEA, AFP or PSA. For example, in 2012, Lin et al. [127] described a triple signal amplification strategy for immunosensing of CEA using AuNPs-decorated poly(styrene-co-acrylic acid) microbeads, onto which signaling antibodies were immobilized. On the other side, the working electrode, a chitosan/RGO-modified GC electrode, was functionalized by the capture antibody. After sandwich-type immunoreaction, the anchored AuNPs further induced the chemical deposition of silver for electrochemical stripping analysis of target antigen (Figure 23B). This method allowed a linear range from 0.5 pg mL^−1^ to 0.5 ng mL^−1^ and a LoD of 0.1 pg mL^−1^. Taleat et al. [128] also described two CEA immunosensors using silver enhancement on AuNPs. The first proposed approach was a label-free impedimetric immunosensor based on polyanthranilic acid-modified graphite SPE where anti-CA 125 were immobilized. Using DPV, without amplification, a LoD of 8 U mL^−1^ was reported. The second approach was based on a sandwich format where a secondary anti-CA 125 antibody, labeled with AuNPs, was added to induced the Ag deposition from a silver solution. The quantity of AgNPs was measured by ASV, which gave a LoD of 2 U mL^−1^. Liu et al. [129] reported a more classical approach with the use of a poly(2-aminothiophenol)-modified GC electrode to immobilize AuNPs onto which anti-CEA was immobilized. With Fe(CN)_6_^3−/4−^ as redox probe diffusing in solution, DPV was used to characterize the recognition of CEA on its antibody. The immunosensor displayed a linear response with CEA from 1 fg mL^−1^ to 10 ng mL^−1^ and a LoD of 0.015 fg mL^−1^. Still using Fe(CN)_6_^3−/4−^ as diffusing redox probe, Sun et al. [130] described an immunosensor for CEA where AuNPs were grafted on a GC electrode by the “double” diazonium route already proposed by Liu et al. the same year, as illustrated on Figure 24A. Anti-CEA was directly adsorbed on these AuNPs. Using CV, the linear detection range was from 10 fg to 100 ng mL^−1^ with a LoD of 3 fg mL^−1^.

These two latter works demonstrated very low detection limits, significantly lower than that reported for more evolved detection schemes. In particular, a LoD of 0.014 fg mL^−1^ for CEA (of around 200 kD) is equivalent to 7 × 10^−23^ mol mL^−1^. At this level of concentration, one has 40 molecules per mL, which impose a particular attention to the experimental conditions.

Still for CEA detection, Wang et al. [131] reported porous PtNPs able to absorb metal ions used for their electrochemical signals. PtNPs were capped with 2-aminoethanethiol then complexed with Cd^2+^ and Cu^2+^ to form PtNPs-Cd^2+^ and PtNPs-Cu^2+^ hybrids. Anti-CEA and anti-AFP were labeled with these hybrids and used in a sandwich immunoassay. Using DPV for reduction of the metal ions present on the electrode, the authors could identify and quantify antibodies. A linear response was measured from 0.05 ng mL^−1^ to 200 ng mL^−1^ for both CEA and AFP, with LoDs of 2 pg mL^−1^ for CEA and 50 pg mL^−1^ for AFP. Also for detection of AFP, Wang et al. [132] described Pd nanoplates onto which AFP antibodies were immobilized, this assembly itself immobilized on a GC electrode. Using Fe(CN)_6_^3−/4−^ in solution as redox probe, AFP was detected from 0.01 to 75.0 ng mL^−1^ with a LoD of 4 pg mL^−1^. Rong et al. [133] reported an immunosensor based on Chi–poly(acrylic acid) nanospheres doped with various metal ions such as copper, cadmium, lead and zinc ions. In the same spirit as reported above with porous PtNPs, those nanospheres were used to graft antibodies and to produce an electrochemical signal by reduction of ions into the corresponding metals at the working electrode, using SWV. With a sandwich-type immunosensor, four tumor markers of pancreatic cancer (CEA, CA199, CA125 and CA242) were detected. The reported linear response ranges were from 0.1 to 100 ng mL^−1^ for CEA and 1 to 150 U mL^−1^ for CA199, CA125 and CA242. LoDs were of 0.02 ng mL^−1^, 0.4 U mL^−1^, 0.3 U mL^−1^ and 0.4 U mL^−1^, respectively (1 enzyme unit (U) = 1 μmol min^−1^). Also for measuring CEA, Lu et al. [134] reported a thionine/infinite coordination polymer functionalized by AgNPs used to increase the overall area as well as the electron transfer rate. Using DPV and Fe(CN)_6_^3−/4−^ in solution, the authors found a linear range between 50 fg mL^−1^ and 100 ng mL^−1^ and a LoD of 0.5 fg mL^−1^. These good results underline the interest of infinite coordination polymer particles. They are of great interest because these structures present a high level of structural tailorability, which allows to tune microporosity and catalytic activity.

Still for CEA detection and using Fe(CN)_6_^3−/4−^ as redox probe in solution, Zhou et al. [135] described a AuNPs-modified Au electrode onto which protein A was adsorbed for oriented immobilization of anti-CEA antibodies. Using EIS and CV, they obtained a linear response to CEA between 1 pg mL^−1^ and 100 ng mL^−1^, and a LoD of 0.1 pg mL^−1^.

Wang et al. [136] reported an enzyme-free paper-based electrochemical immunosensor for detection of CEA. Amino-functionalized graphene/thionine/AuNPs were synthesized and deposited on the electrode along with CEA antibodies. The decreased CV current of Thi was proportional to the concentrations of corresponding antigens within the range 50 pg mL^−1^–500 ng mL^−1^, with a LoD of 10 pg mL^−1^. Li et al. [137] used impedance on interdigitated electrodes (IDEs) to detect CEA with PANi-AuNPs as labels. In a first step, adamantyl-modified capture antibodies were immobilized on IDEs functionalized with carboxymethyl-β-cyclodextrin. After immunoreaction between the capture antibodies and CEA, a PANi-AuNPs-labeled signaling antibodies were added. This recognition sequence led to a linear resistance decrease from 0.1 ng mL^−1^ to 1000 ng mL^−1^ and a LoD of 7 pg mL^−1^. Su et al. [138] reported the use of AuNPs on thionine-modified MoS_2_ nanosheets for electrochemical label-free immunosensing of CEA, where Thi was used not only as a redox indicator but also as a reducing agent for synthesis of the AuNPs. Several shapes of AuNPs were investigated from spherical, triangle, clover-like to flower-like shapes. This system could detect CEA with a LoD of 0.5 pg mL^−1^. At last, also for CEA detection, Huang et al. [139] demonstrated Ag/AuNPs/GR used as both substrate and label of signaling antibodies. Using a sandwich-type immunoassay and CV to measure oxidation/reduction of the Ag^0^/Ag_2_O couple (Figure 24C), the authors reported a linear calibration range from 10 pg mL^−1^ to 1.2 × 10^5^ pg mL^−1^, and a LoD of 8 pg mL^−1^.

**Figure 25:**
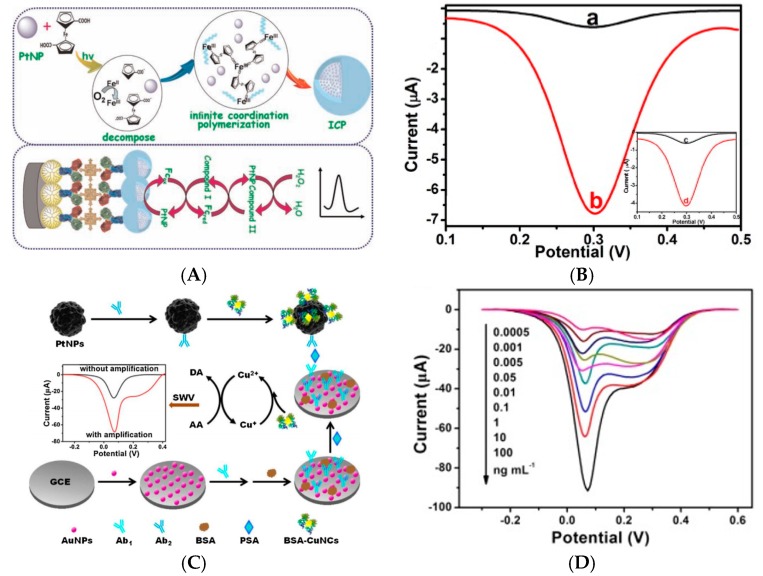
(**A**) (**a**) Formation of ‘all-in-one’ redox-active PtNP@ICP catalyst; and (**b**) coupling this ‘all-in-one’ catalyst on Ab_2_ and transduction for PSA detection; (**B**) DPV of PtNP@ICP-Ab_2_/PSA/Ab_1_-PAMAM-GCE for various PSA concentration (a) 0 ng mL^−1^; and (b) 5 ng mL^−1^ [inset: DPV of ICP-Ab_2_/ PSA/Ab_1_-PAMAM-GCE toward different PSA concentration (c) 0 ng mL^−1^; and (d) 5 ng mL^−1^. Reprinted from [142], Copyright 2015, with permission from Elsevier; (**C**) PtNPs and BSA-copper nanoclusters for detection of PSA; transduction comes from the catalytic oxidation of ascorbic acid; (**D**) SWV responses of the immunosensor for different concentrations of PSA, from 0.0005 ng mL^−1^ to 100 ng mL^−1^. Reprinted from [145], Copyright 2017, with permission from Elsevier.

Detection of other classical proteins were reported, such as HSA (a model protein) and PSA. Omidfar et al. [140] proposed in 2012 a HSA electrochemical immunosensor using HSA-conjugated AuNPs as electrochemical label and a hexagonal mesoporous silica (MCM-41)-PVA composite as immobilization substrate. DPV was used to electrooxidize AuNPs. HSA was detected with a LoD of 1 ng mL^−1^, with a linear range between 0.5 and 200 μg mL^−1^. Wang et al. [141] described an immunosensor to detect PSA using capture antibodies immobilized on mesoporous SiO_2_NPs and AgNPs. HQ added in solution was used as redox probe and its electroactivity measured by CV after Ab-Ag interaction. The peak current was reported to vary linearly with PSA concentration from 0.05 to 50.0 ng mL^−1^, with a LoD of 15 pg mL^−1^. Zhang et al. [142] proposed a PSA sensor using a redox and catalytically active infinite coordination polymer made of Fc-modified PtNP deposited on polyamidoamine dendrimers (Figure 25A). Using a sandwich-type immunoassay format and H_2_O_2_ added in solution, they showed that the reduction current on the PtNPs was proportional to the logarithm of PSA concentration between 1 pg mL^−1^ and 60 ng mL^−1^, with a LoD of 0.3 pg mL^−1^. As for other infinite coordination polymer-based immunosensors, this one demonstrated an excellent LoD.

Also using AuNPs for their catalytic properties toward H_2_O_2_ reduction, Li et al. [143] very recently (2017) described an immunosensor for PSA detection with amino-functionalized Cu_2_O@CeO_2_ core-shell NPs able to bind AuNPs through Au-N bonds. These NPs brought a better electrocatalytic activity towards the reduction of H_2_O_2_ (added as redox probe in solution) than single Cu_2_O or AuNPs alone. The immunosensor exhibited a linear range from 0.1 pg mL^−1^ to 100 ng mL^−1^ with a LoD of 0.03 pg mL^−1^. Also in 2017, Duangkaew et al. [144] described spiky AuNPs for PSA detection. Such spiky AuNPs were used for silver deposition. After immunodetection following a sandwich format, with a capture antibody attached on the electrode surface and a second antibody attached on the signaling NPs, they proceeded to use Linear Scan Voltammetry (LSV) to dissolve Ag^0^. From their own comparison measurements, spiky AuNPs led to over 260-fold of signal increase in comparison with silver enhancement on conventional AuNPs. Two linear relationships between stripping current and PSA concentration were established, in the range of 2–125 pg mL^−1^ and 0.1–10 ng mL^−1^, with a LoD of ca. 1 pg mL^−1^. At last, still in 2017, using the same metal dissolution strategy, Zhao et al. [145] described a PSA sandwich assay with a double signal amplification using copper nanoclusters onto which antibodies were immobilized. The double amplification came from dissolution of Cu at +0.06 V, the resulting Cu^2+^ ions being used to catalyze the oxidation of ascorbic acid (added in solution as redox marker) (Figure 25C). The immunosensor gave a linear range from 0.5 pg mL^−1^ to 100 ng mL^−1^ and a LoD of 150 fg mL^−1^. Figures of merit discussed in this section are summarized in Table 8.

### 3.5. Magnetic Nanoparticles

As shown above, in terms of sensitivity, the main objective now is to increase the ratio between the active surface of the sensor and the volume of analyzed solution. This could be adressed by making highly porous materials; this could also be adressed by using a strategy where the probe molecules, attached on small magnetic NPs, are diffusing in solution for reaction. These magnetic nanoparticles, dispersed in the analytical solution are able to “fish” the analyte before to be magnetically collected on the working electrode for transduction. This has been reported in the literature whether coupled with enzymatic amplification, or not.

#### 3.5.1. Enzyme-Based Immunosensors

**Figure 26:**
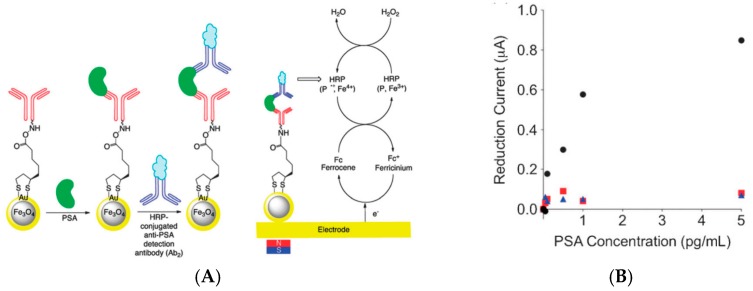

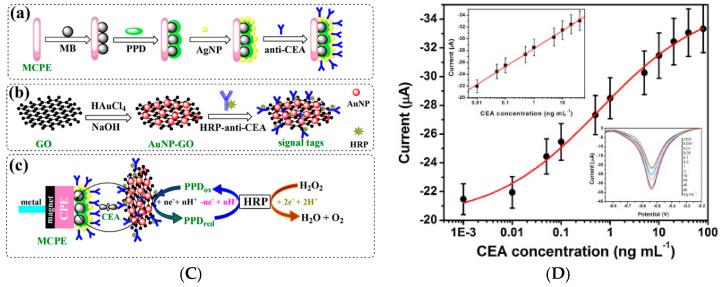
(**A**) Sandwich immunodetection of PSA using Ab1-Au@MNPs, magnetic collection and enzymatic transduction; (**B**) Calibration plots of reduction current versus PSA concentration using planar Ab_1_-modified gold electrode (black dots) and Ab_1_-Au@MNPs (black dots), anti-biotin immobilised on thioctic acid modified Au@MNPs (red squares) and thioctic acid modified Au@MNPs exposed to Ab_1_ without EDC/NHS activation (blue triangles). Reproduced from [146] with permission from The Royal Society of Chemistry. (**C**) Steps required for (**a**) the magnetic capture of PSA using Ab_1_-Au@MNPs; (**b**) construction of the GO-based label; and (**c**) sandwich immunodetection of CEA; (**D**) Calibration plots of the magnetic immunoassay toward CEA standards in pH 5.3 acetic acid salt-buffered saline buffer + 3.0 mM H_2_O_2_. Inset: corresponding linear and DPV curves. Reprinted from [147], Copyright 2012, with permission from Elsevier.

In 2012, Chuah et al. [146] reported electrochemical immunodetection of PSA using gold-coated magnetic Fe_3_O_4_ nanoparticles (Au/Fe_3_O_4_) dispersed in the analytical medium. These nanoparticles were decorated with an alkythiol-modified PSA antibody and incubated with a medium containing PSA. After completion of this step, an HRP-labeled secondary antibody was added, then the Ab_2_-PSA-Ab_1_-NPs were magnetically collected on a gold electrode. After addition of H_2_O_2_ as substrate and ferrocenemethanol as redox mediator for HRP, an amperometric current was measured at +0.15 V vs. Ag/AgCl (Figure 26A). The LoD was found to be 100 fg mL^−1^, much lower than the LoD obtained by the same authors for planar electrodes (ca. 1 pg mL^−1^). Also in 2012, Chen et al. [147] reported a method for detection of CEA using a combination of magnetic NPs covered by an electropolymerized poly(*o*-phenylenediamine) film and silver nanoparticles onto which capture antibodies were immobilized. On the other side, HRP-labelled signaling antibodies were immobilized on AuNP-modified GO sheets, onto which magnetic NPs were also immobilized. The sandwich complexation was initiated by magnetic recollection of the GO sheets (Figure 26C). Upon addition of H_2_O_2_, the authors found a LoD of 1.0 pg mL^−1^ and linearity between 10 pg mL^−1^ and 10 ng mL^−1^ by probing the electroactivity of the poly(*o*-phenylenediamine) film with DPV at pH 5.

**Figure 27:**
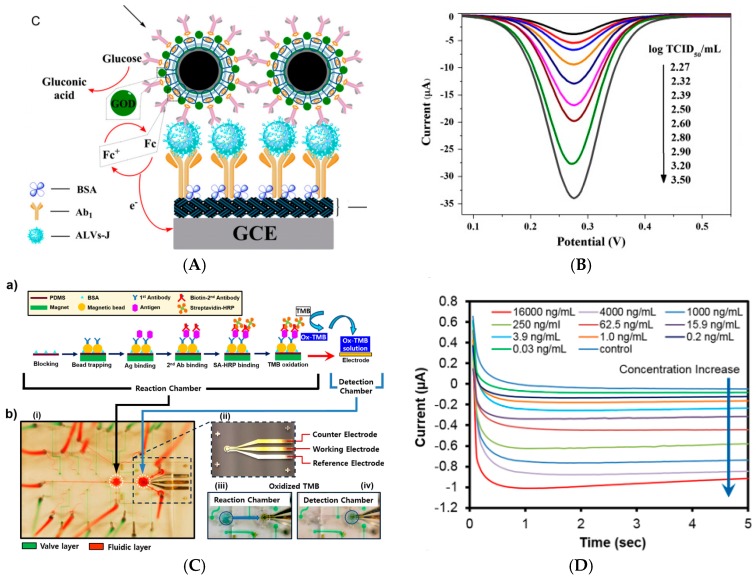
(**A**) Architecture of the magnetic Fe_3_O_4_-based immunosensor. MBs were decorated with GOx, signaling Abs and Fc-labeled *β*-CD. Capture Abs were grafted on GR sheets; (**B**) DPV at pH 7.0 Dependency of the DPV response towards different avian leukosis virus concentrations. Reprinted from [149], Copyright 2013, with permission from Elsevier; (**C**) Design, fabrication, and detection principle of the MB-based microfluidic electrochemical system; (**a**) Immunosensing principle with MB collection and sandwich capture of transferrin; (**b**) The microfluidic sensing chip, (i) photograph of the chip with an integrated microelectrode; (ii) photograph of the microelectrode; (iii) photograph of the reaction chamber with oxidized TMB, (iv) transfer of oxidized TMB to the detection chamber; (**D**) Amperometry measurement at −100 mV for different standard transferrin concentrations. Adapted from [151], licensed under a Creative Commons Attribution 4.0 International License.

Vidal et al. [148] reported a direct competitive immunosensor for ochratoxin A (OCA) using magnetic beads (MBs) of ca. 1 μm in diameter functionalized with OCA antibodies. Using a competitive assay with incubation in a solution containing HRP-conjugated ochratoxin A antibodies and free OCA, MBs were magnetic collected on the electrode, HQ and H_2_O_2_ were added and the HRP activity was measured by DPV. The LoD was ca. 0.10 ng L^−1^. In 2013, Shang et al. [149] reported a sandwich-type electrochemical immunosensor for detection of avian leukosis virus (ALVs-J) using *β*-cyclodextrin-ferrocene (*β*-CD-Fc) host–guest complex on Fe_3_O_4_ MBs. Capture antibodies were coupled to graphene sheets immobilized on the working electrode whereas signaling antibodies and GOx were immobilized on the MBs along with (*β*-CD-Fc). Fc was used as redox mediator for GOx recycling (Figure 27A). Upon addition of glucose in the medium, the detection range was from 200–3000 TCID_50_ mL^−1^ (TCID_50_: 50% tissue culture infective dose), with a low LoD of 150 TCID_50_ mL^−1^.

More recently in 2016, Xu et al. [150] described an impedimetric immunosensor based on the use of MBs for separation and a screen-printed interdigitated microelectrode for detection of Escherichia coli and *Salmonella* Typhimurium. MBs were functionalized with the corresponding capture antibodies, then added in a bacteria suspension for capturing. Then, GOx–Ab conjugate was employed to label the MBs–Ab–cell complexes, which was then magnetically collected on the electrode. Glucose added in solution led to local production of gluconic acid, which was monitored by measuring the impedance of the solution (related to the local pH decrease and ionic strength increase). The LoD was ca. 100 CFU mL^−1^ and the detection range between 1000 and 2000 CFU mL^−1^. Also in 2016, demonstrating that this electrochemical immunosensing is viable in integrated devices for practical applications, Riahi et al. [151] reported a fully-integrated microfluidic platform using MB-labeled capture antibodies and HRP-labeled signaling antibodies. The MB-labeled antibodies were first magnetically collected on the electrode, then the sandwich assay was performed (Figure 27C). Upon addition of H_2_O_2_ and TMB in solution, TMB_Ox_ was amperometrically reduced on the working electrode. Transferrin was detected with a LoD of 0.03 ng mL^−1^. At last, very recently in 2017, Sánchez-Tirado et al. [152] proposed an amperometric immunosensor for the quantification of TGF-β1 using a sandwich-type architecture with MBs functionalized with capture anti-TGF and signaling antibodies labelled with a poly-HRP commercial polymer. Conversely to the previous reference of Riahi et al. they magnetically collected the immunocomplexes on MBs after the sandwich assay. Amperometric measurements were carried out at −0.20 V by adding H_2_O_2_ onto the electrode surface in the presence of HQ as redox mediator. Calibration showed linearity between 15 and 3000 pg mL^−1^ and a LoD of 10 pg mL^−1^.

#### 3.5.2. Enzyme-Free Immunosensors

**Figure 28:**
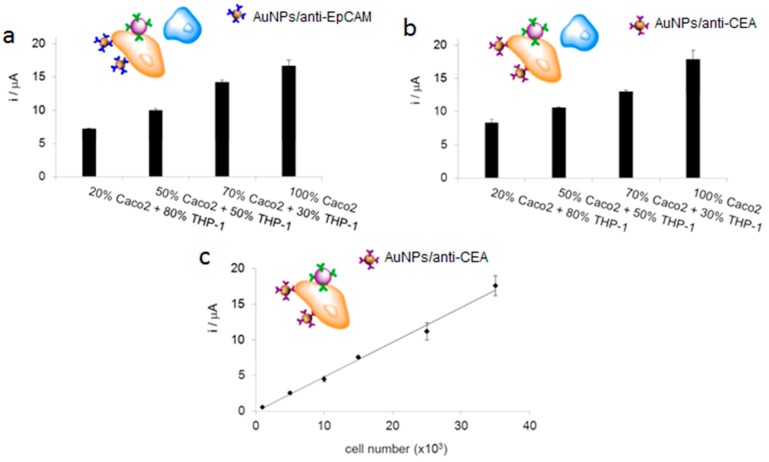
Electrochemical detection of Caco2 cells using MBs/antiEpCAM for selective capture. (**a**,**b**) Effect of the number of cells on the analytical signal for different target (Caco2) and control cells (THP-1) proportions for (**a**) simultaneous labeling with AuNPs/antiEpCAM and (**b**) simultaneous labeling with AuNPs/anti-CEA. 100% corresponds to a cell amount of 5 × 104; (**c**) Calibration plot for an increasing number of Caco2 cells (in the absence of THP-1) with simultaneous labeling with AuNPs/anti-CEA. Reprinted with permission from [153]. Copyright 2012 American Chemical Society.

In 2012, Maltez-da Costa et al. reported a simple way to monitor cancer circulating cells (CTCs) using nanoparticles [153]. They combine capturing antibody-functionalized magnetic beads (MBs) and labeling through antibody-modified AuNPs (antibody: anti-EpCAM, Epithelial Cell Adhesion Molecule) able to electrocatalyze the hydrogen evolution reaction (HER). A LoD of 2.2 × 10^2^ cells was achieved for human colon adenocarcinoma cells (CaCo2) (Figure 28), without the need for a reactant to be added in solution.

**Figure 29:**
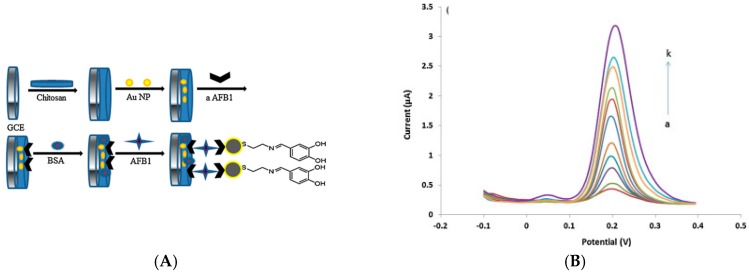
(**A**) GCE/chitosan/AuNP/anti-AFB1/AFB1/anti-AFB1/MB/catechol immunoensor; (**B**) DPV recorded for GCE/chitosan/AuNP/anti-AFB_1_/BSA/AFB_1_/anti-AFB_1_/catechol–Au–Fe_3_O_4_ immunoelectrode as a function of AFB_1_ concentration from 0 ng mL^−1^ to 110 ng mL^−1^ in pH 7.0 (scan rate 50 mV s^−1^). Reprinted from [154], Copyright 2013, with permission from Elsevier;

Masoomi et al. [154] reported in 2013 a non-enzymatic sandwich-type magnetic electrochemical immunosensor for determination of aflatoxin B1. They used gold-coated Fe_3_O_4_ magnetic MBs functionalized with both 3-((2-mercaptoethylimino)methyl)benzene-1,2-diol and the specific aflatoxin antibody. The GC working electrode was also modified with the capture anti-aflatoxin B_1_. Binding of the antigen brought the modified MBs on the electrode surface. Oxidation of the immobilized benzene-1,2-diol (catechol moiety) was used as redox indicator using DPV (Figure 29). Aflatoxin B_1_ was detected within the range 0.6–110 ng mL^−1^, with a LoD of 0.2 ng mL^−1^.

**Figure 30:**
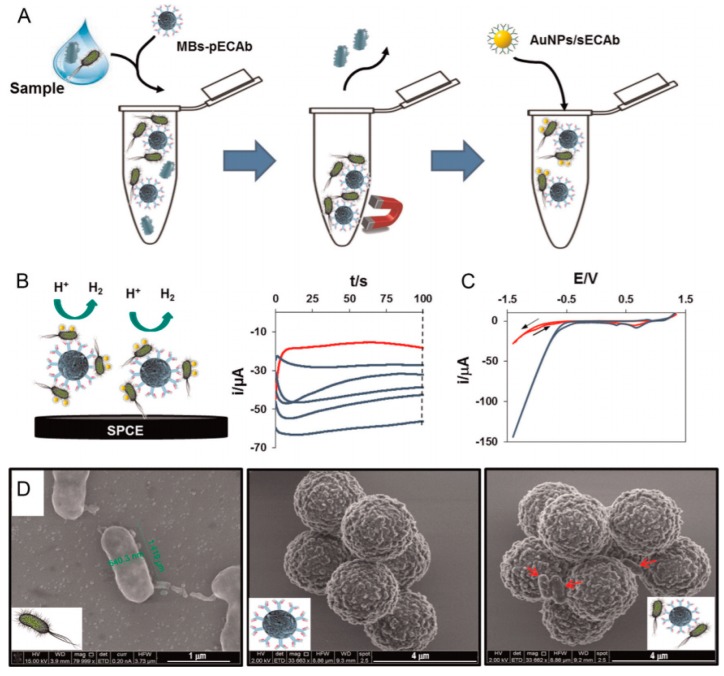
Schematic experimental design. (**A**) *E. coli* was captured by MBs modified with primary antibodies, and subsequently labelled with AuNPs carrying secondary antibodies; (**B**) Left: detection of labeled *E. coli* through the hydrogen evolution reaction electrocatalyzed by the AuNP labels. Right: Chronoamperograms registered in 1 M HCl, during the HER applying a constant voltage of 1.0 V for increasing *E. coli* concentrations (from top to bottom: 0, 102, 5 102, 103, 104 and 105 CFU mL^−1^); (**C**) Cyclic voltammograms in 1 M HCl for 0 (red curve) and 10^5^ (blue curve) CFU mL^−1^; scan rate: 50 mV/s. (**D**) SEM image for heat-killed *E. coli* (left) and antibody modified MBs before (center) and after (right) incubation with 10^5^ CFU mL^−1^ of *E. coli* (bacteria are pointed by red arrows). Reprinted from [156], Copyright 2015, with permission from Elsevier.

Also in 2013, Chan et al. [155] described a non-competitive *E. coli* immunosensor using MB-modified capture antibodies for concentration of *E. coli* from the solution bulk onto a nanoporous alumina membrane. Quantification of *E. coli* was made by monitoring the membrane impedance after collection. This method gave a LoD of 10 CFU mL^−1^. Still for *E. coli* detection, Hussein et al. [156] described an immunosensing assay based on the electrocatalytic properties of gold nanoparticles (AuNPs) towards hydrogen evolution, combined with superparamagnetic microbeads (MBs) as capture pre-concentration and purification platform. After capture and sandwiching with secondary antibody-modified AuNPs, transduction was made using chronoamperometry on SPCEs. LoDs of 148, 457 and 309 CFU mL^−1^ were obtained in buffer solution, minced beef and tap water samples, respectively (Figure 30).

More classically, Wu et al. [157] described Pt–Fe_3_O_4_ MBs for detection of squamous cell carcinoma antigen (SCC-Ag). Curiously, Pt/Fe_3_O_4_ MBs were not used for their magnetic properties but to enhance the catalytic electroreduction of H_2_O_2_ added in solution. Nitrogen-doped graphene sheets were used to immobilize anti-SCC capture antibodies whereas signaling antibodies were adsorbed onto the Pt/Fe_3_O_4_ NPs. Using a sandwich type immunoassay, the sensor gave a linear range between 0.05 and 18 ng mL^−1^, with a LoD of 15 pg mL^−1^. In 2014, Yang et al. [158] also reported a non-enzymatic electrochemical immunosensor for clenbuterol (CLB) detection using magnetic gold-coated Fe_3_O_4_ nanoparticles in a competitive immunoassay. The carbon SPE working electrode was modified with graphene and Nafion. Au/Fe_3_O_4_ MBs were modified with CLB then magnetically collected on the electrode. A competitive immunoassay was used with CLB and anti-CLB added simultaneously in solution. CV and DPV were used to evaluate antibody binding on the electrode, using diffusing Fe(CN)_6_^3−/4−^ as redox probe. The signal was linear with clenbuterol between 0.5 and 200.0 ng mL^−1^, with a LoD of 0.22 ng mL^−1^. As for the previous article, even if using magnetic MBs, it seems that they were not used to collect the analyte in solution, which is probably the cause of their relatively high detection limits. Ahmadi et al. [159] also reported an electrochemical immunosensor using Au/Fe_3_O_4_ MPs as labels, for detection of digoxin. Anti-digoxin antibodies were immobilized on a PVA-modified SPE and MBs were decorated with digoxin and capture anti-digoxin. In a competitive assay were digoxin and AuMB/digoxin/anti-digoxin complex were present in solution, AuCl_4_^−^ was electrochemically reduced into Au^0^ on the Au/MBs using DPV. The current depending on the surface concentration of Au/MBs, digoxin was detected in the range 0.5–5 ng mL^−1^, with a LoD of 50 pg mL^−1^.

Rivas et al. reported an alzheimer disease biomarker detection using IrO_2_NPs [160]. IrO_2_ were used as tags for their electrocatalytic activity towards water oxidation reaction (WOR). Citrate-capped IrO_2_NPs were modified with anti-Apolipoprotein E antibodies (αApoE) for the detection of the ApoE alzheimer disease biomarker, with a detection limit of 68 ng mL^−1^. Using IrO_2_ NPs allowed signal generation in the same medium where the immunoassay takes place (PBS, pH 7.4). de la Escosura-Muñiz et al. reported in 2015 also Alzheimer’s disease biomarkers detection but using porous magnetic microspheres and labelling with AuNPs [161]. The magnetic microparticles were functionalized by carboxylic groups for covalent grafting of antibodies, allowing an efficient capturing of the Alzheimer biomarkers directly from serum samples. AuNP were used to electrocatalyze the hydrogen evolution reaction. The LoD was estimated between 20 and 80 pg mL^−1^.

**Figure 31:**
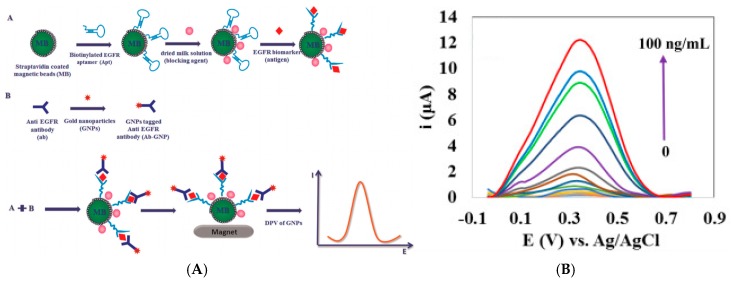
(**A**) Magnetic beads functionalized with capture aptamers, and transduction using AuNPs-labelled signaling antibodies in a sandwich assay; (**B**) Corresponding DPVs for different concentrations of EGFR in the range of 1–40 ng mL^−1^. Reprinted from [162], Copyright 2015, with permission from Elsevier.

In 2015, Ilkhani et al. [162] reported the detection of EGFR using an original electrochemical aptamer/antibody sandwich immunosensor. The capture aptamer was immobilized on streptavidin-coated MBs and the anti-human EGFR antibody was conjugated to AuNPs and, after magnetic collection on the electrode, was used as electrochemical signaling probe by dissolution of the AuNPs in HCl (Figure 31). The detection range was from 1 to 40 ng mL^−1^, with a LoD of 50 pg mL^−1^. At last, very recently, Wang et al. [163] described interferon-γ detection using antibody-functionalized MBs labeled with AuNPs. These MBs were conjugated with a second interferon-γ antibody and multiple CdS nanoparticles (CdSNPs), forming a sandwich MPs/interferon-γ/AuNPs/CdSNPs complex. After magnetic separation, SWASV was used on carbon SPE for redissolution of the CdSNPs. The response was linear 0.01 to 1 IU mL^−1^. Figures of merit discussed in this section are summarized in Table 9.

### 3.6. Dendrimers

Dendrimers, regularly branched spherical molecules, are generally used for encapsulation of functional molecules, or for their surface functionalities (active sites on the external dendrons). Dendrimers are classified by generation, i.e., the number of repeated branching cycles that are performed for their synthesis (3 repeated branching results in a third generation dendrimer, G3). For immunosensors, they mainly bring high densities of coupling groups, therefore high surface densities of probes of electroactive molecules. They participate to the research of the highest surface-to-volume ratio, which is the key factor of highly sensitive sensors.

#### 3.6.1. Enzyme-Based Immunosensors

**Figure 32:**
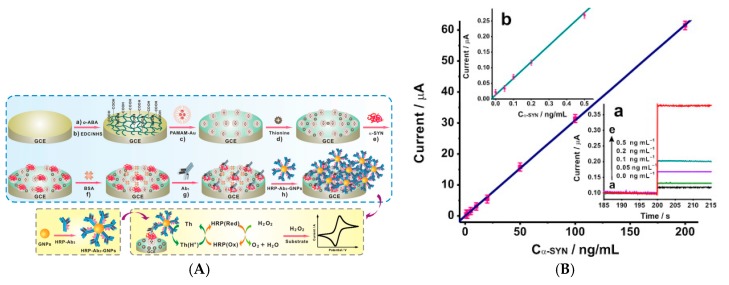

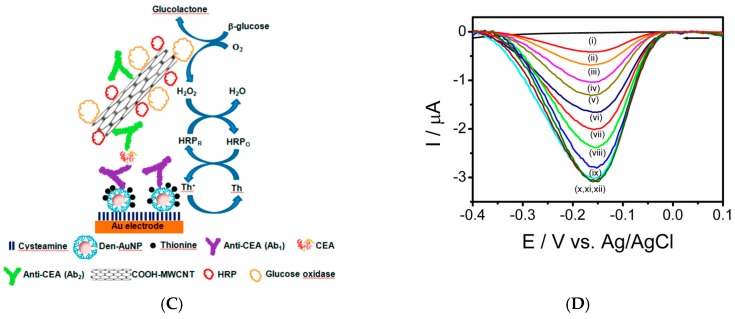
(**A**) PAMAM-based electrochemical immunosensor for detection of α-SYN. (**B**) Calibration curves obtained with {HRP-Ab_2_-GNPs}/Ab_1_/α-SYN/Th/PAMAM-Au/*o*-ABA/GCE immunosensor for different concentrations of α-SYN. Inset: (a) amperometric responses of the immunosensor in PBS (pH 6.5) to α-SYN at various concentrations of (a) 0, (b) 0.05, (c) 0.1, (d) 0.2, (e) 0.5 ng mL^−1^; (b) amplification of calibration curve in the low α-SYN concentration. Reprinted from [164], Copyright 2012, with permission from Elsevier. (**C**) Detection principle of a CEA immunosensor based on a sandwich capture and a HRP-GOx bienzymatic cascade. (**D**) SWV responses recorded for Au/Cys/Den/AuNP/Th-based CEA immunosensors at (i) blank noise and at various CEA concentrations: (ii) 10, 50, 100 pg mL^−1^, 0.5, 1.0, 5.0, 10, 50 ng mL^−1^, 0.1, 0.5 and 1.0 μg mL^−1^. Reprinted with permission from [165]. Copyright 2013 American Chemical Society.

An et al. [164] reported detection of α-synuclein (α-SYN), a neuronal protein, based on a dual signal amplification strategy. AuNPs decorated with G4 PAMAM (polyamidoamine) were covalently bound on poly-*o*-aminobenzoic acid electropolymerized on the electrode surface, onto which α-SYN was adsorbed as capture antibodies. Then, α-SYN/HRP/AuNPs were introduced as signaling antibodies. The electrocatalytic response upon oxidation of Thi (introduced in solution) was monitored by CV and gave a linear response between 20 pg mL^−1^ and 200 ng mL^−1^, with a LoD of 15 pg mL^−1^ (Figure 32A).

Jeong et al. [165] also described an electrochemical immunosensor based on an amine-terminated polyamidoamine (PAMAM) dendrimer for detection of CEA. Anti-CEA (as capture probe) and the mediator Thi were immobilized on AuNPs encapsulated in the dendrimers. In a sandwich format, signaling anti-CEA coupled to GOx+HRP-modified MWCNTs were used for transduction (Figure 32C). The detection range was between 10.0 pg mL^−1^ and 50.0 ng mL^−1^ with a LoD of ca. 4 pg mL^−1^. At last, Kavosi et al. [166] described a triple signal amplification strategy for immunosensing of PSA by modification of GC electrodes with GO/Chi onto which PSA antibody and Thi were covalently attached. Recognition was made through a sandwich-type immunoreaction between PSA, anti-PSA immobilized on the GO/Chi interface, and HRP-functionalized PSA aptamers immobilized on PAMAM/AuNPs. Using DPV, the LoD was 10 fg mL^−1^ and the detection range between 0.1 pg mL^−1^ and 90 ng mL^−1^.

#### 3.6.2. Enzyme-Less Immunosensors

Gao et al. [167] reported PAMAM/carbon dots modified by AuNPs for immunosensing of AFP. PAMAM/carbon dots/AuNPs were immobilized on the electrode surface, followed by anti-AFP immobilization. Upon complexation of AFP on these capture antibodies, the redox process of a diffusion probe Fe(CN)_6_^3−/4−^ was slowed down. Changes in current as well as in impedance were used to quantify AFP within a wide range from 100 fg mL^−1^ to 100 ng mL^−1^, with a LoD of 0.025 pg mL^−1^. Kavosi et al. [168] also described an electrochemical immunoassay for detection of AFP. This immunosensor was constructed by covalent immobilization of PAMAM dendrimer-encapsulated AuNPs on a Au electrode, followed by covalent immobilization of ethyleneamineviologen (Vio) as electrochemical redox marker and AFP monoclonal antibody. The Au/PAMAM nanocomposite was used to increase the electrode surface area and accelerate the electron transfer kinetics. Upon immunorecognition of the immobilized AFP to its antibody, the Vio peak current decreased due to the hindered electron transfer reaction on the electrode surface. Through DPV, AFP was detected over a wide linear range (0.001–45 ng mL^−1^) and a LoD of 130 fg mL^−1^. This result underline that dendrimers afford a much better sensitivity than planar electrodes when considering immobilized redox probes.

**Figure 33:**
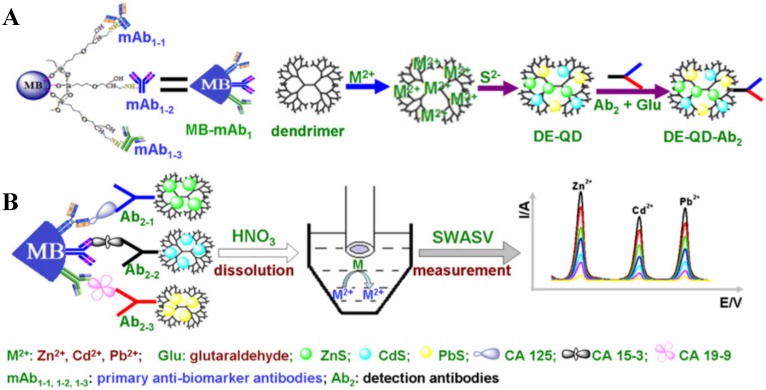
(**A**) Use of signaling antibodies coupled to dendrimers (PAMAN) containing metal sulfides, and (**B**) subsequent electroreduction by ASV for detection. This method allows detecting several antibody-antigen complexes. Reprinted from [169], Copyright 2013, with permission from Elsevier.

Using stripping instead of a diffusing redox probe, Tang et al. [169] reported detection of three protein biomarkers (CA 125, CA 15-3, and CA 19-9) in one single immunoassay. They used antibody-decorated magnetic beads as immunosensing probes, as reported in the previous section, and PAMAM dendrimer-metal sulfide quantum dot (QD) as nanolabels. The MBs were functionalized by co-immobilization of the three capture antibodies (anti-CA 125, anti-CA 15-3 and anti-CA 19-9) and PAMAM/QDs containing CdS, ZnS and PbS were used for their labeling (Figure 33). ASV of cadmium, zinc, and lead led to a dynamic range of 0.01–50 U mL^−1^ and a LoD of 5 mU mL^−1^.

Finally, Chandra et al. [170] reported an IgG electrochemical immunosensor using a redox-active ferrocenyl G2 dendrimer (G_2_Fc) as redox marker, onto which the capture antibody was immobilized. The presence of the captured IgG generates steric hindrance which was evidenced by a change in electroactivity of the immobilized Fc. The LoD was 2 ng mL^−1^.

As shown, dendrimers generally bring lower detection limits than conventionally grafted redox probes; one of the obvious drawbacks, however, looks to be the complexity of the sensor’s architecture, which impedes reproducibility and make these sensors hardly applicable. Figures of merit discussed in this section are summarized in Table 10.

### 3.7. Ionic Liquids

We reviewed here in this section some recent advances in the use of ionic liquids in the construction of electrochemical immunosensors. As discussed above, the use of metal nanoparticles or graphene, or a combination of the two, were used as building materials to improve the sensing performances. However, water dispersability of these materials is low and they present a strong tendency to agglomerate into multilayer graphite of bundles of NPs through π–π stacking or Van der Waals interaction, which limits their application. It was shown that ionic liquid, used for surface functionalization of such NPs, could be a solution.

**Figure 34:**
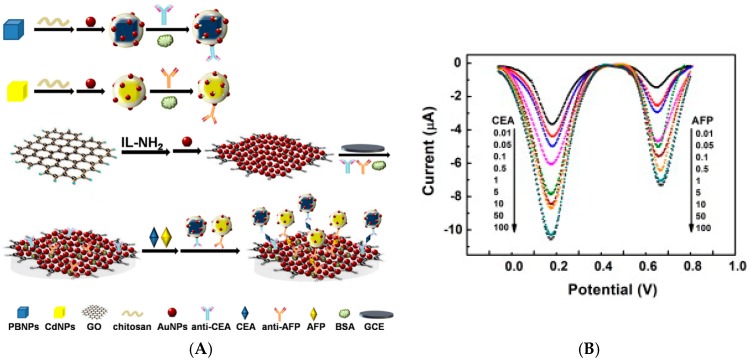
(**A**) CEA and AFP immunosensor based on metal NPs and Prussian blue NPs immobilized on IL-modified GO sheets; (**B**) DPV responses for different concentration of CEA and AFP in PBS, pH 6.5. Reproduced from [172], Copyright 2014, with permission from Elsevier.

In 2013, Liu et al. [171] reported ionic liquid-functionalized GR sheets loaded with AuNPs onto which antibodies were immobilized, for detection of CEA. Using DPV and Fe(CN)_6_^3−/4−^ as diffusing redox probe, this immunosensor showed a linear detection range from 1 fg mL^−1^ to 100 ng mL^−1^, and a LoD of 0.1 fg mL^−1^. Liu and Ma [172] described an IL-functionalized RGO material for CEA and AFP detection. Due to the modification with 1-aminopropyl-3-methylimidazolium chloride, GO sheets were easily dispersed in aqueous solution to form a homogeneous colloidal suspension. Signaling antibodies were coupled to Prussian blue NPs and CdNPs and used in a sandwich immunoassay (Figure 34). Using DPV, the linear ranges were from 0.01 to 100 ng mL^−1^ for both CEA and AFP. The LoD was 10 pg mL^−1^ for CEA and 6 pg mL^−1^ for AFP. 

Ruiyi et al. [173] reported an immunosensor for microcystin-LR, a family of water contaminant composed on a stable heptapeptide produced mainly by common cyanobacteria. GC electrodes were modified with GO and AuNPs, and 2,5-di-(2-thienyl)-1-pyrrole-1-(*p*-benzoic acid) was grafted to immobilize the microcystin-LR antibodies. The IL (1-isobutyl-3-methylimidazolium bis(trifluoro-methanesulfonyl)imide) was then dropped on the electrode surface and finally microcystin-LR antibody was covalently bound to the conducting polymer film. Electrochemical detection was made with DPV in the range 10^−16^–8 × 10^−15^ M using Fe(CN)_6_^3−/4−^ added in solution, and the authors reported an extremelly low LoD of 4 × 10^−17^ M (i.e., 4 × 10^−17^ g mL^−1^). This article was very similar to another one of the same authors published one year before [174], reporting an immunosensor for aflatoxin B_1_, for which the LoD was of 1 fM and the linear range from 3.2 fM to 0.32 pM, measuring the charge transfer resistance by EIS.

Roushani el al. [175] reported in 2016 an immunoassay for detection of hCG. First, PtNPs were immobilized on a composite layer of graphene, chitosan and an ionic liquid (1-methyl-3-octyl imidazolium tetrafluoroborate). The amine groups of the antibody were covalently attached to PtNPs, whereas rutin was used in solution as redox probe. Upon biorecognition of hCG on the antibody, peak current of rutin decreased. Through DVP measurements, it was found that hCG could be detect within a linear range between 2 and 350 mIU mL^−1^, with a LoD of 0.4 μIU mL^−1^. 

**Figure 35:**
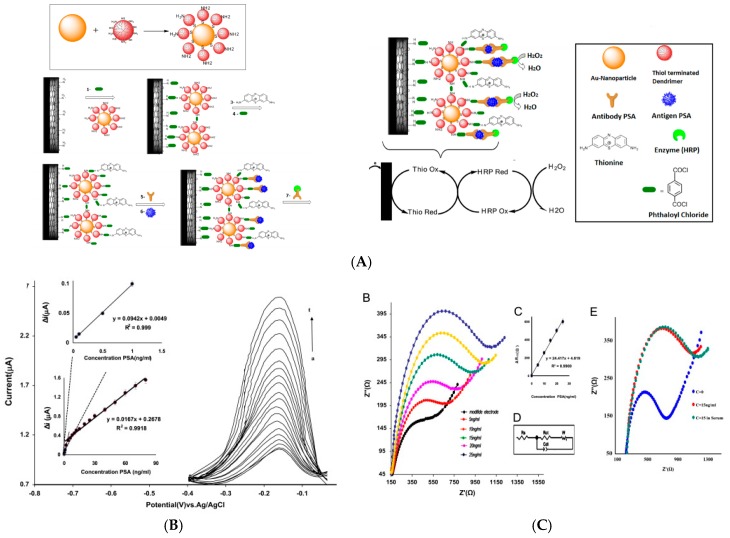
(**A**) PSA immunosensor using AuNPs–PAMAM for signaling and MWCNT/IL/CS as substrate, and transduction with HRP and thionine as immobilized mediator; (**B**) CVs of the immunosensor after incubation with PSA from O up to 80 ng mL^−1^ in PBS, pH 7 + 2.5 mM H_2_O_2_. Insets, plots of the immunosensor response vs. PSA concentrations; (**C**) Nyquist curves for 2.5 mM [Fe(CN)_6_]^3−/4−^ in 0.1 M KCl recorded at anti-PSA/AuNPs–PAMAM-dendrimer/MWCNTs/IL/Chit/GC after incubation with 5, 10, 15, 20 and 25 ng mL^−1^ PSA. Insets are plot of *R*_ct_ vs. PSA concentration and the equivalent circuit used to fit the experimental impedance data. On the right, Nyquist curves for the immunosensor in the presence of 15 ng mL^−1^ PSA and in serum spiked with 15 ng mL^−1^ PSA. Adapted from [176], Copyright 2014, with permission from Elsevier.

Finally, Kavosi et al. [176] reported detection of PSA where most of the above-reviewed strategies were cumulated in a single sensor. Anti-PSA and thionine (as redox mediator) were immobilized onto AuNPs–PAMAM and MWCNT/IL/CS nanocomposite as substrate (Figure 35). Capture and transduction was made by a sandwich assay between anti-PSA immobilized on the MWCNTs/IL/CS/AuNPs–PAMAM and anti-PSA labeled with HRP-labeled signaling antibodies. The electrocatalytic reduction of H_2_O_2_ by HRP was monitored by DPV. The calibration curve for PSA was linear up to 80 ng mL^−1^, with a LoD of 1 pg mL^−1^. Figures of merit discussed in this section are summarized in Table 11.

### 3.8. Unconventionnal Electrochemical Immunosensors Working in Organic Solvents

As reviewed above, the most targeted analytes are organic compounds which are poorly soluble in water. Consequently, it is of interest to develop electrochemical immunosensors able to work directly in organic solvent or at least in a mixture of organic and aqueous solvents. Immunoassays in organic phase have been reported since at least two decades [177], investigated in optical [178] and gravimetric [179] devices recently, but stayed scarce for electrochemical immunoassays.

In 2012, Tomassetti et al. [180] reported an immunosensor for the analysis of atrazine in olive oil, using HRP as antibody label, through a competitive immunoreaction. A LoD of about 50 nM was achieved. The immunorecognition was performed in oil, and the electrochemical detection in PBS. The same authors reported in 2015 detection of three kind of pesticides (triazinic, organophosphates and chlorurates) in sunflower oil, still through a competitive immunodetection process which took place in an *n*-hexane–chloroform mixture, followed by electrochemical detection (HRP-labelled Ab) performed in decane. The LoD was 10 nM. [181,182].

### 3.9. Unconventionnal Substrates: Nanochannels for Electrochemical Immunodetection

**Figure 36:**
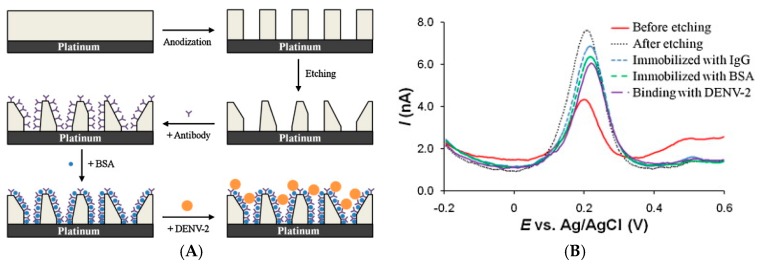
(**A**) Biosensor’s structure and detection procedure. DENV-2 corresponds to the dengue virus; (**B**) Differential pulse voltammograms of alumina-modified electrode obtained in 1.0 mM ferrocenemethanol, 0.1 M phosphate buffered saline, pH 7.4 after each step of the biosensor construction procedure, DENV-2 concentration = 10^2^ pfu mL^−1^; Adapted from [186], Copyright 2012, with permission from Elsevier.

**Figure 37:**
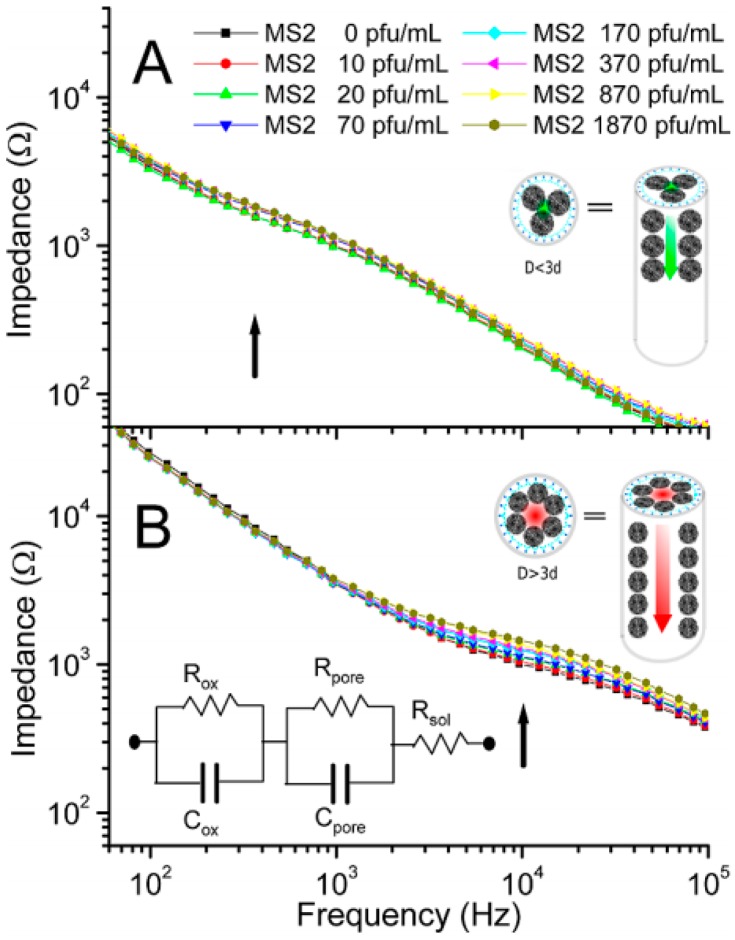
(**A**) Bode plots for different concentrations of MS2 bacteriophage. (**B**) It is shown that the smaller pores (73 nm) are less efficient due to compromised accessibility of the walls to viruses when the diameter of the pore is smaller than 3 times that of the virus, while larger pores (97 nm) are not affected. R_sol_ is the resistance of solution above the membrane, R_pore_ and C_pore_ are the resistance and capacitance of the pores in the membrane, and R_ox_ and C_ox_ are the resistance and capacitance of the oxide layer. Adapted with permission from [190]. Copyright 2016 American Chemical Society.

Even before the period considered in this review, unconventional porous substrates have been reported for electrochemical immunodetection [183,184,185]. These works were continued; in 2012 then 2013, Cheng et al. [186] and Peh et al. [187] developed an electrochemical sensor for detection of dengue virus, based on a porous alumina membrane on a platinum electrode, functionalized with antibodies specific for dengue. Upon recognition of the virus by the antibodies, blocking of the pores were characterized by DPV (example below) and impedance spectroscopy. The detection limit was found at ca. 0.5 PFU mL^−1^ (Figure 36). More recently, [188,189] Espinoza-Castañeda et al. and de la Escosura-Muñiz et al. described nanochannel arrays made from nanoparticles assembled on a solid surface, for immunodetection through a principle like that described above. The blocking in the nanochannel was characterized by a decrease in the voltammetric signal of a redox indicator, or an increase in impedance. Very recently, Chaturvedi et al. [190] described again the use of anodized alumina membranes, to measure ionic conductivity through pores decorated by capture probes. MS2 bacteriophage was chosen as an example. A LoD of ca. 7 pfu mL^−1^ was found. Examples of EIS spectra are given in Figure 37. These approaches have been recently reviewed [191].

## 4. Conclusions and Perspectives

**Figure 38:**
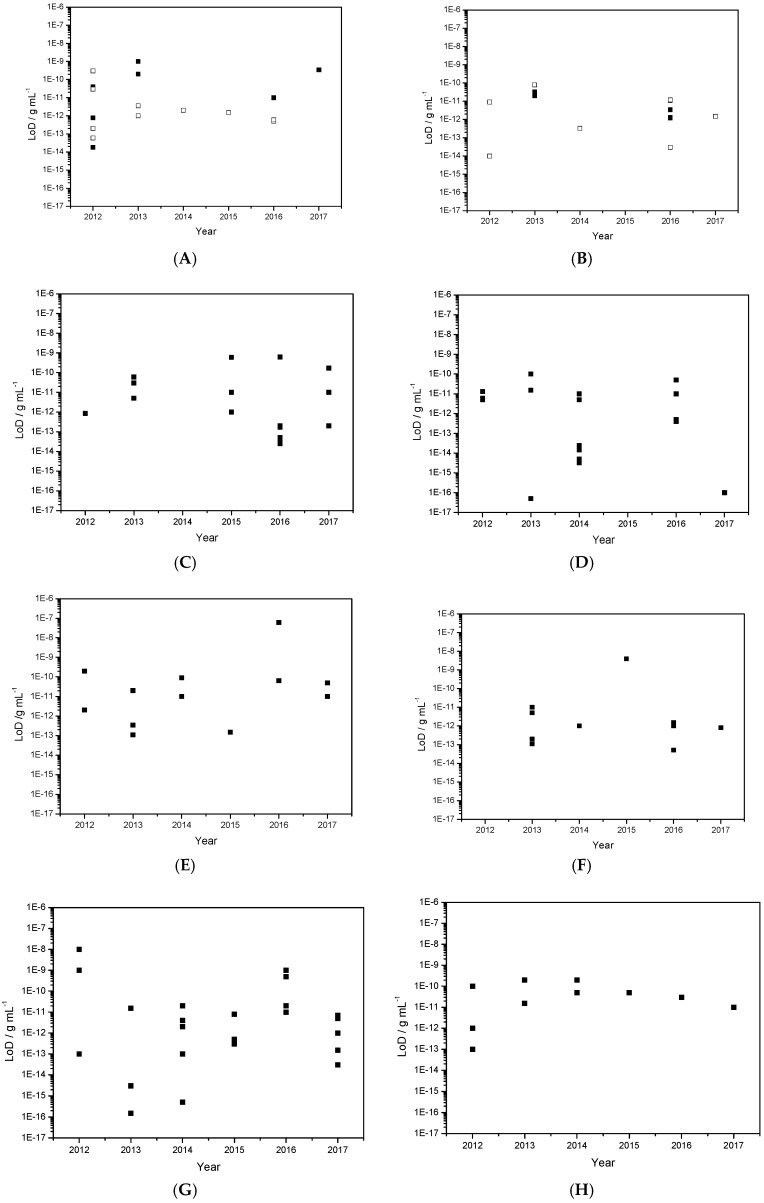

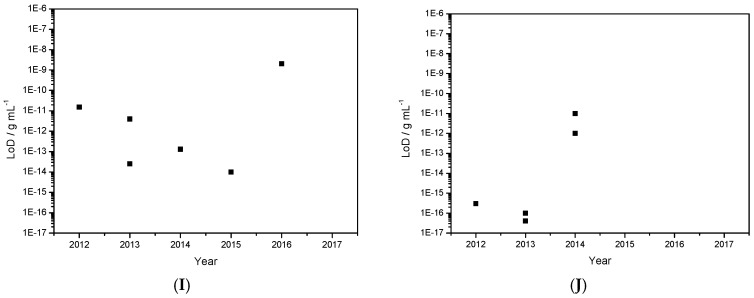
LoDs of electrochemical immunosensors, from 2012 to 2017, using (**A**) conventional electrode substrates (open squares, enzyme-based sensors; plain square, enzyme-free sensors); (**B**) carbon nanostructures (open squares, enzyme-based sensors; plain square, enzyme-free sensors); (**C**) enzyme-free immunosensors with graphene and diffusing redox probe; (**D**) enzyme-free immunosensors with graphene and immobilized redox probes; (**E**) enzymatic immunosensors using graphene; (**F**) enzymatic immunosensors with metal or metal oxide nanoparticles; (**G**) enzyme-free immunosensors with metal or metal oxide nanoparticles; (**H**) magnetic nanoparticles; (**I**) dendrimers; (**J**) ionic liquids.

As shown on Figure 38, conventional electrode substrates (Figure 38A) gave higher LoDs than those featuring carbon nanostructures (Figure 38B). For both, enzymes used as amplifiers allowed to lower the LoD, as expected. This behavior is not general, however. Indeed, for the most studied immunosensors during this period, those based on metal or metal oxide nanoparticles, the best sensitivities were obtained with enzyme-free architectures (Figure 38G). On the contrary, the less studied ones are those featuring ionic liquids (Figure 38J) which, in addition, were not reported these very last years, which tends to indicate that the initial promises of IL in terms of sensitivity were not kept. Dendrimers were also rarely reported (Figure 38I), even if LoDs achieved with these structures are significantly lower than those reported for other architectures. Enzyme-free immunosensors with graphene and immobilized redox probes (Figure 38D) gave better results compared to those with diffusing redox probes (Figure 38C), and one of the lowest LoDs in average. This approach must therefore be emphasized as one of the most promising ones for electrochemical immunosensors.

Also, enzymatic immunosensors with metal or metal oxide nanoparticles (Figure 38F) gave better results compared to graphene only (Figure 38E). It is also noteworthy that immunosensors based on magnetic nanoparticles (Figure 38H) did not allow to lower detection limits as it could have been expected.

All this allow to conclude that the way to improve the detection limits of immunosensors is to increase the molecular and redox probe densities, both immobilized on high surface-to-volume nanoparticles. Because the most general transduction pathway implies a sandwich architecture with enzymes or artificial catalytic sites coupled to the signaling antibody, which significantly amplifies currents, one could consider that improvement perspectives rely on catalytically (non-enzymatic) amplified sandwich immunoassays using substrates of specific area as high as possible.

The ratio between the number of available recongnition sites and the number of available target molecules in the analyzed solution must absolutely be considered. It is indeed not consistent to target very low concentrations in a small volume of solution (that is, very small amounts of targets) with large electrodes having so high densities of molecular probes that the probes/targets ratio tends to zero; in such case, the sensor should not be sensitive at all. Finally, even if this point is extremely scarsely pointed out in immunosensors’ publications, authors should also consider that the quality of the antibodies, and particularly Ab-Ag affinities, is of the highest importance and may be also a significant factor for improvement.

## Figures and Tables

**Table 1:** Glossary of acronyms used in this review.

Acronyms	Definitions	Acronyms	Definitions
Ab	Antibody	INF	interferon-γ
ACTH	Adrenocorticotropin	OEG	Oligoethyleneglycol
AFP	α-FetoProtein	o-PD	o-PhenyleneDiamine
Ag	Antigen	ITO	Indium Tin Oxide
Ag	Silver	LoD	Limit of Detection
AlkP	Alkaline Phosphatase	LSV	Linear Sweep Voltammetry
ALV	Avian Leukosis Virus	MB	Magnetic Bead
APN	AdiPoNectin	MCF	Mesoporous Carbon Foam
APTES	3-AminoPropylTriEthoxySilane	MEG	Multi-layer epitaxial graphene
Ara-h1	ARAchis Hypogaea antigen	MMP-9	Matrix MetalloPeptidase-9
ASV	Adsorptive Stripping Voltammetry	MPS	MesoPorous Silica
ATZ	Atrazine	MWCNT	MultiWalled Carbon NanoTube
Aβ (1–42)	Amyloid beta 1–42	NB	Nile Blue
BQ	Benzoquinone	NP	NanoParticle
CA	Cancer Antigen	OCA	OChratoxin A
CA	Carbohydrate Antigen	OEG	OligoEthyleneGlycol
C-dot	Carbon dot	PAMAM	PolyAMidoAMine
CEA	CarcinoEmbryonic Antigen (CEA)	PANi	Polyaniline
CFU	Colony Forming Unit	pAPP	p-AminoPhenyl Phosphate
Chi	Chitosan	PB	Prussian Blue
ChOx	Choline Oxidase	PBA	BisPhenol A
CLB	Clenbuterol	PDA	PolyDopAmine
CoPc	Cobalt PhtaloCyanine	PDDA	Poly(DiallylDimethylAmmonium)
CRP	C-reactive protein	PDMS	PolyDiMethylSiloxane
cTnT	Cardiac Troponin T	PE-CVD	Plasma Enhanced Chemical Vapor Deposition
CV	Cyclic voltammetry	PEI	PolyEthyleneImine
DPV	Differential Pulse Voltammetry	*Pf*HRP2	*Plasmodium falciparum* histidine-rich protein 2
DTSP	DiThiobisSuccinimidyl Propionate	PNIPAAm	poly(N-isopropylacrylamide)
*E. coli*	*Escherichia coli*	PPy	PolyPyrrole
ECP	Electrically Conducting Polymer	PSA	Prostate Specific Antigen
EE2	EthynylEstradiol	PVA	PolyVinylAlcohol
EGFR	Epidermal Growth Factor Receptor	RCA	Rolling Circle Amplification
EIS	Electrochemical Impedance Spectroscopy	RGO	Reduced Graphene Oxide
EpCAM	Epithelial Cell Adhesion Molecule	SA	Sulfonamide
Fc	Ferrocene	SAM	Self-Assembled Monolayer
GC	Glassy Carbon	SCCA	Squamous Cell Carcinoma Antigen
GCE	Glassy Carbon Electrode	scFv	Single Chain Variable Fragment
GNR	Gold NanoRod	SPE	Screen-Printed electrode
GNS	Graphene NanoSheets	SU-8	Epoxy-based negative photoresist
GO	Graphene Oxide	SWASV	Square Wave Adsorptive Stripping Voltammetry
GPP	*N*-glycosylated pentapeptide	SWCNT	Single-Walled Carbon NanoTube
GQD	Graphene Quantum Dots	SWV	Square Wave Voltammetry
GR	Graphene	TB	Toluidine Blue
HbA1c	Glycated Gaemoglobin	TC	TetraCycline
hCG	Human Chorionic Gonadotropin	TCID	Tissue Culture Infective Dose
Hb	Haemoglobin	TGF	Transforming Growth Factor
HE 4	Human Epididymis protein 4	Thi	Thionine
HIV	Human Immunodeficiency Virus	TMB	3,3′,5,5′-Tetramethylbenzidine
HQ	Hydroquinone	TNF-α	Tumor Necrosis Factor α
HRP	Horse Radish Peroxidase	TREM-1	Triggering Receptor Expressed on Myeloid cells 1
HSA	Human Serum Albumin	tTG	Tissue TransGlutaminase
HSL	Hormone-Sensitive Lipase	UME	UltraMicroElectrode
ICP	Infiniet Coordination Polymer	Vangl1	human Vang-like protein
Ide	InterDigitated Electrode	VH	Variable Heavy
IgA	ImmunoGlobulin A	Vio	Viologen
IgG	ImmunoGlobulin G	VL	Variable Light
IL	Ionic Liquid	α-SYN	α-SYNuclein
IL-6	InterLeukin-6	*β*CD	*Β*-CycloDextrin

**Table 2:** Figures of merit of enzyme-based and enzyme-free immunosensors using conventional electrode substrates.

Target	Probe	LoD	Method	Reference
AbHIV	Peptides	1 ng mL^−1^	AlkP/DPV	[12]
IgG	Ag	1.4 μg mL^−1^	HRP/CV—Sandwich	[13]
Estradiol	Ab	0.77 pg mL^−1^	HRP/CV—Competitive	[14]
CEA	Ab	40 pg mL^−1^	HRP—Sandwich	[15]
ACTH	Ab	18 fg mL^−1^	AlkP/DPV—Competitive	[16]
Sulfonamide	Ab	200–500 pg mL^−1^	HRP—Competitive	[17]
CA-125	Ab	0.1 U mL^−1^	GOx—Sandwich	[18]
TNF-α	Ab	10 pg mL^−1^	HRP/CV—Sandwich	[19]
Tyrosine kinase	Ab	337 pg mL^−1^	HRP—Sandwich	[20]
TREM-1	Ab	30 pg mL^−1^	FeCN_6_^3−/4−^/EIS	[21]
Atrazine	Hapten	0.2 ng L^−1^	ECP/SWV—Competitive	[22]
HbA1c	Peptide	-	Fc/CV—Competitive	[23]
Bisphenol A	Hapten	2 pg mL^−1^	ECP/SWV—Competitive	[24]
Acetaminophen	Hapten	1.5 pg mL^−1^	ECP/SWV—Competitive	[25]
HE4	Ag	0.06 ng L^−1^	DNA/DNA—Sandwich	[26]
Okadaic acid	Ag	0.3 ng mL^−1^	FeCN_6_^3−/4−^/EIS	[27]
EGFR	Ag	1 pg mL^−1^	FeCN_6_^3−/4−^/EIS	[28]
Cortisol	Ag	3.6 ng L^−1^	FeCN_6_^3−/4−^/CV	[29]
Porcine albumin	Ag	0.5 pg mL^−1^	FeCN_6_^3−/4−^/CV	[30]
Hemagglutinin	scFv	0.6 pg mL^−1^	FeCN_6_^3−/4−^/EIS	[31]

**Table 3:** Figures of merit of selected immunosensors using carbon nanostructures.

Target	Probe	LoD	Method	Reference
*E. coli*	Ab	5 × 10^3^ CFU mL^−1^	HRP/CV	[32]
Cardiac troponin T	Ab	0.033 ng mL^−1^	HRP/Fe(CN)_6_^3−/4−^/CV	[33]
Aflatoxin B_1_	Ab	3.5 pg mL^−1^	AlkP/a-NP/DPV	[34]
TGF protein	Ab	1.3 pg mL^−1^	HRP/HQ/Amperometry	[35]
PSA protein	Ab	20 pg mL^−1^	HRP/TH/DPV	[36]
α-Fetoprotein	Ab	0.011 ng mL^−1^	HRP/PB/DPV	[37]
Procalcitonin	Ab	1.23 pg mL^−1^	ChOx/H_2_O_2_-CoPC/DPV	[38]
Aflatoxin B_1_	Ab	0.08 ng mL^−1^	Fe(CN)_6_^3−/4−^/CV	[39]
Endosulphan	Hapten	0.01 pg mL^−1^	Fc/SWV	[40]
Gonadotrophin	Ab	3 μIU mL^−1^	HRP/CV	[41]
α-Transglutaminase	Ab	-	AlkP/Ag^0^-Ag^+^	[42]
Zearalenone	Ab	1.5 pg mL^−1^	Fe(CN)_6_^3−/4−^/CV	[43]
IgG	Ag	9 pg mL^−1^	AuNP/AuCl_4_^−^/DPV	[44]
8-Isoprostane	Ag	12 pg mL^−1^	HRP/HQ/Competition/CV	[45]
AFP protein	Ag	0.33 pg mL^−1^	GOx/HRP/Fe(CN)_6_^3−/4−^/CV	[46]
CRP protein	Ag	-	Fe(CN)_6_^3−/4−^/CV	[47]
Vangl1 protein	Ag	0.03 pg mL^−1^	AuNPs/H_2_O_2_/Amperometry	[48]

**Table 4:** Figures of merit of selected enzyme-less immunosensors using graphene and diffusing redox probe.

Target	Probe	LoD	Method	Reference
β-Lactoglobulin	Ab	0.85 pg mL^−1^	Fe(CN)_6_^3−/4−^/DPV	[52]
AFP protein	Ab	5 pg mL^−1^	H_2_O_2_/AuPdNPs/CV	[53]
AFP protein	Ab	0.03 ng mL^−1^	Fe(CN)_6_^3−/4−^/TiO_2_/GR/Chi/AuNPs/DPV	[54]
AFP protein	Ab	0.06 ng mL^−1^	Fc/DPV	[55]
AFP protein	Ab	0.01 ng mL^−1^	Gr/SnO_2_/Au Fe(CN)_6_^3−/4−^/DPV	[56]
AFP protein	Ab	12 mU mL^−1^	Fe(CN)_6_^3−/4−^/DPV	[57]
CA 15-3	Ab	0.033 pg mL^−1^	H_2_O_2_/CeO_2_NPs	[58]
hCG	Ab	0.62 ng mL^−1^	Fe(CN)_6_^3−/4−^/CV	[59]
PSA protein	Ab	0.6 ng mL^−1^	Fe(CN)_6_^3−/4−^/CV	[60]
PSA protein	Ab	1 pg mL^−1^	H_2_O_2_/ZnO/AgNPs/CV	[61]
PSA protein	Ab	0.05 pg mL^−1^	H_2_O_2_/Fc/DPV	[62]
PSA protein	Ab	0.01 ng mL^−1^	Fe(CN)_6_^3−/4−^/AgNPs/CV	[63]
*E. Coli*	Ab	3.8 CFU mL^−1^	Fe(CN)_6_^3−/4−^/CuONPs/EIS	[64]
SCCA protein	Ab	25 fg mL^−1^	H_2_O_2_/PtPdCuNPs	[65]
CEA protein	Ab	0.17 pg mL^−1^	H_2_O_2_/PtV_2_O_5_	[66]
CEA protein	Ab	0.2 pg mL^−1^	H_2_O_2_/Fe_3_ONPs	[67]
CEA protein	Ab	0.2 pg mL^−1^	H_2_O_2_/IrNPs	[68]
CRP protein	Ab	0.17 ng mL^−1^	H_2_O_2_/AgPtNPs	[69]

**Table 5:** Figures of merit of selected enzyme-free immunosensors using graphene and immobilized redox probe.

Target	Probe	LoD	Method	Reference
PSA	Ab	13 pg mL^−1^	Graphene/MB/CV	[70]
Kanamycin	Ab	6 pg mL^−1^	GR/PtNPs/Thi/CV	[71]
Kanamycin	Ab	15 pg mL^−1^	Ag/Fe_3_O_4_ NPs/Thi/SWV	[72]
CEA protein	Ab	5 pg mL^−1^	PdCu-GR/Thi/CV	[73]
CEA protein	Ab	10 pg mL^−1^	Carbon CMK-3/Thi/DPV	[74]
CEA protein	Ab	0.05 fg mL^−1^	AuNPs/Thi	[75]
CEA protein	Ab	0.024 pg mL^−1^	AgNPs/ASV	[76]
CEA protein	Ab	0.1 ng mL^−1^	GR/Toluidine blue	[77]
CEA protein	Ab	3.3 fg mL^−1^	AuNPs/Toluidine blue/DPV	[78]
CEA protein	Ab	0.4 pg mL^−1^	GR/CHI/Fc	[79]
CEA protein	Ab	0.5 pg mL^−1^	GO/AuNPs/NB	[80]
AFP protein	Ab	5 pg mL^−1^	PdNPs/RGO/Amperometry	[81]
AFP protein	Ab	0.1 fg mL^−1^	Cu_2_O/GO/TB/SWV	[82]
Avian leukosis virus	Ab	115 TCID_50_ mL^−1^	Fe_3_O_4_/CuNPs/ASV	[83]
Tumor cells	Ab	10 cells mL^−1^	CdTe/SWV	[84]
Interleukin-6	Ab	5 fg mL^−1^	GR nanoribbon/AgNPs	[85]
Estradiol	Ab	0.015 pg mL^−1^	Fe_3_O_4_/Pb^2+^/ASV	[86]
Cardiac troponin-I	Ab	0.05 ng mL^−1^	GO/AuNPs/Fc/SWV	[87]
Myoglobin	Ab	0.01 ng mL^−1^	Graphene QDs/EIS	[88]
miRNA	Ab	5 fmol L^−1^	RGO/PCE/SWV	[89]

**Table 6:** Figures of merit of selected enzyme-based immunosensors using graphene.

Target	Probe	LoD	Method	Reference
IgG	Ab	0.2 ng mL^−1^	RGO/HRP/HQ/Sandwich	[90]
IgG	Ab	10 pg mL^−1^	RGO/AuNPs/HRP/Anilin	[91]
PSA	Ab	2 pg mL^−1^	GR/AuNPs/HRP	[92]
CEA	Ab	0.35 pg mL^−1^	GR/AuNPs/Thi/HRP/DPV	[93]
CEA	Ab	90 pg mL^−1^	GR/Lectin/Fe(CN)_6_^3−/4−^/DPV	[94]
CEA	Ab	0.01 ng mL^−1^	N-doped GR/Thi/HRP/DPV	[95]
17β-Estradiol	Ab	0.02 ng mL^−1^	GO/HQ/HRP/DPV	[96]
Ethynylestradiol	Ab	65 pg mL^−1^	AgNPs/SiO_2/_GO/HQ/HRP	[97]
Cortisol	Ab	0.05 ng mL^−1^	AuNPs/GO/o-PD/HRP/DPV	[98]
MMP-2	Ab	0.11 pg mL^−1^	AuNPs/GR/GO/Thi/DPV	[99]
HIV-p24 protein	Ab	0.15 pg mL^−1^	GO/Thi/HRP	[100]
Adiponectin	Ab	60 ng mL^−1^.	RGO/Thi/HRP/	[101]
CA19-9 carbohydrate	Ab	6 mU mL^−1^	Au@PdNPs/GR/Thi/HRP	[102]
miRNA	Ab	10 fmol L^−1^	RGO/MWCNT/HQ/HRP	[103]

**Table 7:** Figures of merit of selected enzymatic immunosensors using metal or metal oxide nanoparticles.

Target	Probe	LoD	Method	Reference
IgG	Ag	0.01 ng mL^−1^	AuNPs/HRP/H_2_O_2_	[104]
CEA protein	Ag	0.11 pg mL^−1^	AuNPs/HRP/H_2_O_2_/PB	[105]
CEA protein	Ag	1.3 pg mL^−1^	AuNPs/GOx/Fe(CN)_6_^3−/4−^/EIS	[106]
CEA, PSA proteins	Ag	1 pg mL^−1^	PPy NPs/HRP	[107]
CEA protein	Ag	1 pg mL^−1^	AuNPs/MnO_2_/GOx/TMB	[108]
AFP protein	Ag	0.2 pg mL^−1^	AuNPS/HRP	[109]
AFP protein	Ag	1.5 pg mL^−1^	AuNPs/HRP/polyaniline	[110]
TNF-α protein	Ag	5 pg mL^−1^	Au nanorods/GOx/Fc/SWV	[111]
Interferon-γ	Ag	0.05 pg mL^−1^	AuNPs/HRP/H_2_O_2_/HQ	[112]
Influenza virus	Ag	1 pg mL^−1^	ZnO nanorods/HRP/TMB	[113]
EpCAM protein	Ag	0.8 pg L^−1^	AgNPs/HRP/H_2_O_2_	[114]
Ara-H1 protein	Ag	4 ng mL^−1^	AuNPs/AlkP/Ag^0^/ASV	[115]

**Table 8:** Figures of merit of selected non-enzymatic immunosensors using metal or metal oxide nanoparticles.

Target	Probe	LoD	Method	Reference
IgG	Ab	0.02 ng mL^−1^	AuNPs/PPy/Fe(CN)_6_^3−/4−^/EIS	[116]
Cancer cells	Ab	4 × 10^3^ cells	AuNPs/CV	[117]
HbA1c protein	Peptide	10 ng mL^−1^	AuNPs/Ru(NH_3_)_6_^2+/3+/^EIS	[118]
Dengue NS1 protein	Ab	0.5 ng mL^−1^	Fc-labelled Ab/EIS	[119]
Mycotoxins	Ab	1 ng mL^−1^	AuNP/PPy/Fe(CN)_6_^3−/4−^	[120]
Amyloid beta 1–42	Ab	5 pg mL^−1^	AuNPs/Fe(CN)_6_^3−/4−^	[121]
*Pf*HRP2	Ab	70 ng L^−1^	PtNPs/MB/Hydrazine	[122]
*E. coli*	Ab	400 cells mL^−1^	MWCNT/PbS/SWASV	[123]
CA72-4 carbohydrate	Ab	0.10 U mL^−1^	AuNPs/PANi/H_2_O_2_	[124]
Atrazine	Ab	0.02 ng mL^−1^	Fe(CN)_6_^3−/4−/^DPV	[125]
Cortisol	Ab	0.4 ng L^−1^	ZnO nanorods/Fe(CN)_6_^3−/4−/^EIS	[126]
CEA protein	Ab	0.1 pg mL^−1^	AuNPs/Ag enhancement/ASV	[127]
CEA protein	Ab	8 U mL^−1^	AuNPs/Ag enhancement/ASV	[128]
CEA protein	Ab	0.015 fg mL^−1^.	AuNPs/Fe(CN)_6_^3−/4−/^DPV	[129]
CEA protein	Ab	3 fg mL^−1^	AuNPs/Fe(CN)_6_^3−/4−/^CV	[130]
CEA protein	Ab	2 pg mL^−1^	PtNPs-Cd^2+/^DPV	[131]
AFP protein	Ab	4 pg mL^−1^	Pd nanoplates/Fe(CN)_6_^3−/4−^	[132]
CEA protein	Ab	0.02 ng mL^−1^	PAA NPs/Cd^2+^/SWV	[133]
CEA protein	Ab	0.5 fg mL^−1^	AgNPs/THI/Fe(CN)_6_^3−/4^/DPV	[134]
CEA protein	Ab	0.1 pg mL^−1^	AuNPs/Fe(CN)_6_^3−/4−/^EIS	[135]
CEA protein	Ab	10 pg mL^−1^	GR/Thi/AuNPs	[136]
CEA protein	Ab	7 pg mL^−1^	PANi-AuNPs/EIS/conductivity	[137]
CEA protein	Ab	0.5 pg mL^−1^	AuNPs/Thi/MoS_2_	[138]
CEA protein	Ab	8 pg mL^−1^	Ag/AuNPs/Ag^0^/Ag_2_O/CV	[139]
HSA protein	Ab	1 ng mL^−1^	SiO_2_NPs/AuNPs oxidation/DPV	[140]
PSA protein	Ab	15 pg mL^−1^	SiO_2_NPs/HQ/CV	[141]
PSA protein	Ab	0.3 pg mL^−1^	Fc-PtNPs/H_2_O_2_	[142]
PSA protein	Ab	0.03 pg mL^−1^	Cu_2_O@CeO_2_NPs/AuNPs/H_2_O_2_	[143]
PSA protein	Ab	1 pg mL^−1^	AuNPs/Ag enhancement/LSV	[144]
PSA protein	Ab	150 fg mL^−1^	Cu nanoclusters/ascorbic acid	[145]

**Table 9:** Figures of merit of selected immunosensors using magnetic nanoparticles.

Target	Probe	LoD	Method	Reference
PSA protein	Ab	100 fg mL^−1^	Au/Fe_3_O_4/_HRP/Fc/H_2_O_2_	[146]
CEA protein	Ab	1.0 pg mL^−1^	AgNPs/HRP/H_2_O_2_/o-PD/CV	[147]
Ochratoxin A	Ab	0.10 ng L^−1^	MBs/HRP/H_2_O_2_/HQ/DPV	[148]
ALVs-J	Ab	150 TCID^50^ mL^−1^	*β*CD-Fc/Fe_3_O_4_/GOx	[149]
*E. coli*	Ab	1000 CFU mL^−1^	MBs/GOx/Glucose/EIS	[150]
Transferrin	Ab	0.03 ng mL^−1^	MBs/HRP/H_2_O_2_/TMB	[151]
TGF-β1	Ab	10 pg mL^−1^	MBs/polyHRP/HQ/H_2_O_2_	[152]
CTC	Ab	2.2 × 10^2^ cells	AuNPs/HER	[153]
Aflatoxin B1	Ab	0.2 ng mL^−1^	Au/Fe_3_O_4_ MBs/Catechol/DPV	[154]
*E. coli*	Ab	10 CFU mL^−1^	MBs/Nanoporous Al_2_O_3/_EIS	[155]
*E. coli*	Ab	300 CFU mL^−1^	AuNPs/HER	[156]
SCC-Ag	Ab	15 pg mL^−1^	Pt/Fe_3_O_4_ MBs/H_2_O_2_	[157]
Clenbuterol	Ab	0.2 ng mL^−1^	Au/Fe_3_O_4_ MBs/Fe(CN)_6_^3−/4−^/DPV	[158]
Digoxin	Ab	50 pg mL^−1^	Au/Fe_3_O_4_ MPs/AuCl_4_^−^/DPV	[159]
Apolipoprotein	Ab	68 ng mL^−1^	IrO_2_ NPs/Amperometry	[160]
Apolipoprotein	Ab	20–80 pg mL^−1^	MBs/AuNPs/Amperometry	[161]
EGFR	Aptamer	50 pg mL^−1^	MBs/AuNPs/ASV	[162]
Interferon-γ	Ab	0.01 IU mL^−1^	MPs/AuNPs/CdSNPs/SWASV	[163]

**Table 10:** Figures of merit of selected immunosensors using dendrimers.

Target	Probe	LoD	Method	Reference
α-Synuclein protein	Ab	15 pg mL^−1^	PAMAM/AuNPs/HRP/Thi/CV	[164]
CEA protein	Ab	4 pg mL^−1^	PAMAN/AuNPs/GOx-HRP/Thi	[165]
PSA protein	Ab	10 fg mL^−1^	PAMAM/AuNPs/HRP	[166]
AFP protein	Ab	0.025 pg mL^−1^	PAMAM/C-dots/AuNPs/Fe(CN)_6_^3−/4−^	[167]
AFP protein	Ab	130 fg mL^−1^	PAMAM/AuNPs/Viologen	[168]
CA 125 protein	Ab	5 mU mL^−1^	MBs/PAMAM/QDs/CdS/ASV	[169]
IgG	Ab	2 ng mL^−1^	anti-IgG/G_2_Fc/Fc	[170]

**Table 11:** Figures of merit of selected immunosensors using ionic liquids.

Target	Probe	LoD	Method	Reference
CEA protein	Ab	0.1 fg mL^−1^	IL/GR/AuNPs/Fe(CN)_6_^3−/4−/^DPV	[171]
CEA protein	Ab	10 pg mL^−1^	IL/RGO/PB/DPV	[172]
Microcystin-LR	Ab	0.04 fg mL^−1^	IL/GO/AuNPs/PPy/Fe(CN)_6_^3−/4−/^DPV	[173]
Aflatoxin B1	Ab	0.3 fg mL^−1^	IL/GO/AuNPs/PPy/Fe(CN)_6_^3−/4−/^EIS	[174]
hCG hormone	Ab	0.4 μIU mL^−1^	IL/PtNPs/Rutin/DPV	[175]
PSA protein	Ab	1 pg mL^−1^	MWCNT/IL/CS/AuNPs/PAMAM/HRP	[176]
